# Discovery of Dual Aβ/Tau Inhibitors and Evaluation of Their Therapeutic Effect on a *Drosophila* Model of Alzheimer’s Disease

**DOI:** 10.1021/acschemneuro.2c00357

**Published:** 2022-11-29

**Authors:** Annachiara Gandini, Ana Elisa Gonçalves, Silvia Strocchi, Claudia Albertini, Jana Janočková, Anna Tramarin, Daniela Grifoni, Eleonora Poeta, Ondrej Soukup, Diego Muñoz-Torrero, Barbara Monti, Raimon Sabaté, Manuela Bartolini, Giuseppe Legname, Maria Laura Bolognesi

**Affiliations:** † Department of Pharmacy and Biotechnology, Alma Mater Studiorum - University of Bologna, Via Belmeloro 6, I-40126Bologna, Italy; ‡ Department of Neuroscience, Laboratory of Prion Biology, Scuola Internazionale Superiore di Studi Avanzati (SISSA), Via Bonomea 265, I-34136Trieste, Italy; § Pharmaceutical Sciences Postgraduate Program, Center of Health Sciences, Universidade do Vale do Itajaí, Rua Uruguai 458, 88302-202Itajaí, Santa Catarina, Brazil; ∥ Biomedical Research Center, University Hospital Hradec Kralove, 500 00Hradec Kralove, Czech Republic; ⊥ Department of Life, Health and Environmental Sciences, University of L’Aquila, Via Vetoio, Coppito II, 67100L’Aquila, Italy; # Laboratory of Medicinal Chemistry (CSIC Associated Unit), Faculty of Pharmacy and Food Sciences, and Institute of Biomedicine (IBUB), University of Barcelona (UB), Av. Joan XXIII 27-31, E-08028Barcelona, Spain; ∇ Department of Pharmacy and Pharmaceutical Technology and Physical Chemistry, Faculty of Pharmacy and Food Science, University of Barcelona, Av Joan XXIII 27-31, E-08028Barcelona, Spain

**Keywords:** aggregation inhibitors, multitarget-directed ligands, bivalent ligands, β-amyloid, tau protein, protein aggregates

## Abstract

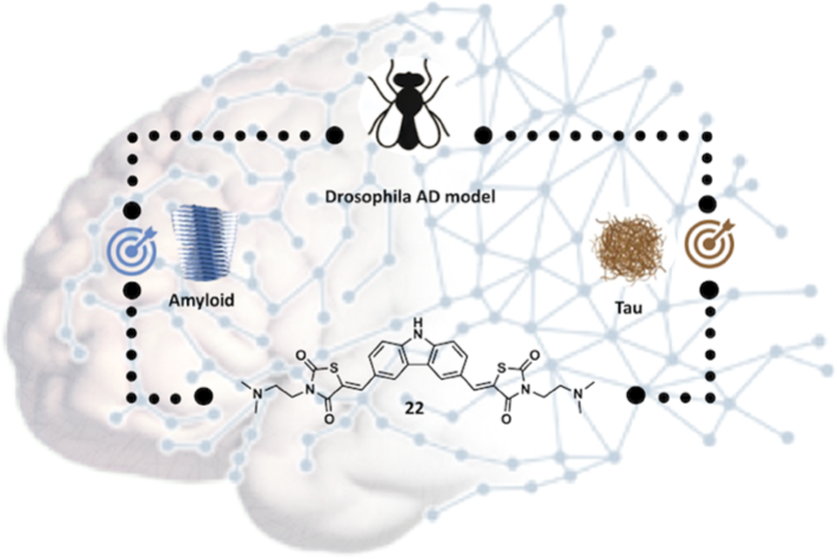

Alzheimer’s disease (AD), the most common type of dementia, currently represents an extremely challenging and unmet medical need worldwide. Amyloid-β (Aβ) and Tau proteins are prototypical AD hallmarks, as well as validated drug targets. Accumulating evidence now suggests that they synergistically contribute to disease pathogenesis. This could not only help explain negative results from anti-Aβ clinical trials but also indicate that therapies solely directed at one of them may have to be reconsidered. Based on this, herein, we describe the development of a focused library of 2,4-thiazolidinedione (TZD)-based bivalent derivatives as dual Aβ and Tau aggregation inhibitors. The aggregating activity of the 24 synthesized derivatives was tested in intact *Escherichia coli* cells overexpressing Aβ_42_ and Tau proteins. We then evaluated their neuronal toxicity and ability to cross the blood–brain barrier (BBB), together with the *in vitro* interaction with the two isolated proteins. Finally, the most promising (most active, nontoxic, and BBB-permeable) compounds **22** and **23** were tested *in vivo*, in a *Drosophila melanogaster* model of AD. The carbazole derivative **22** (20 μM) showed extremely encouraging results, being able to improve both the lifespan and the climbing abilities of Aβ_42_ expressing flies and generating a better outcome than doxycycline (50 μM). Moreover, **22** proved to be able to decrease Aβ_42_ aggregates in the brains of the flies. We conclude that bivalent small molecules based on **22** deserve further attention as hits for dual Aβ/Tau aggregation inhibition in AD.

## Introduction

Alzheimer’s disease (AD) is the most common cause of dementia and one of the most important unmet medical needs worldwide. In 2020, over 50 million people were living with dementia globally, a figure set to increase to 152 million by 2050.^1^ Importantly, the most alarming data is the lack of truly disease-modifying treatments in the clinic. Actually, the year 2021 set a milestone for the field, with the approval of aducanumab by the Food and Drug Administration (FDA)—the first new medication in 18 years. Aducanumab, which is a monoclonal antibody (mAb) targeting amyloid-β (Aβ), has been approved for the treatment of patients with mild to moderate AD. However, its approval remains controversial, due to ambiguous clinical results on its efficacy and the refusal of marketing authorization by the European Medicines Agency (EMA) in December 2021. Thus, even though its approval has raised hopes for AD patients, postapproval trials will be key to truly understand its clinical benefits.^2^ Notably, two further anti-amyloid mAbs, lecanemab and donanemab, have been granted breakthrough therapy designation by the FDA.^3^

The aducanumab story, unfortunately, indicates that it is still difficult to translate promising preclinical data into clinical efficacy.^4^ Probably, the removal of plaques *per se* is neither sufficient to clearly improve brain function and to boost cognition nor to delay AD progression in AD patients.^5^ A possible explanation is that a greater insight into the interrelationship between Aβ and Tau is needed to understand AD pathogenesis and explain the failure of previous therapeutic strategies.^5^ Aβ and Tau aggregates are well-defined pathological hallmarks and validated targets in AD, but how they interact has been largely unknown. There is accumulating evidence that Aβ and Tau proteins may act synergistically to cause synaptic dysfunction, neurofibrillary tangle-mediated neuronal loss, and behavioral deficits. A recent publication has shed light on how Aβ and Tau might cooperate in causing AD phenotypes.^6^ Aβ seems to initiate the neuroinflammation process, making synapses vulnerable to Tau-associated molecular changes, such as the loss of synaptic proteins. Thus, although Tau may be sufficient to induce neuroinflammation, Aβ plaques could induce inflammatory changes that exacerbate degeneration when Tau is present.^6^ Another recent seminal study has revealed how Aβ and Tau synergize to damage the functional integrity of neural circuits and provided a likely explanation for the disappointing results from anti-Aβ clinical trials.^7^

Based on this perspective, the development of multitarget-directed ligands (MTDLs)^8^ able to target both Aβ and Tau aggregation processes, might be an option to pursue.^9^

In recent years, few AD drug discovery programs have focused on dual Aβ/Tau inhibitors, able to interfere with protein–protein interactions (PPIs), to avoid propagation, or to prevent fibril formation. However, not many of these inhibitors were purposely designed to target Aβ and Tau aggregation simultaneously; most of the reports focused on acetylcholinesterase (AChE) inhibitors where Aβ and Tau antiaggregation was an activity designed-in by random screening. Indeed, the rationale modulation of PPIs by small molecules has been considered quite challenging for several reasons: lack of druggable active sites or pockets; paucity of high-resolution structural information on amyloid aggregates; and multiple electrostatic, polar, and hydrophobic interactions across a large interface.^10^ To overcome these issues, knowledge-centric ligand-based strategies may be of help.

**Figure 1:**
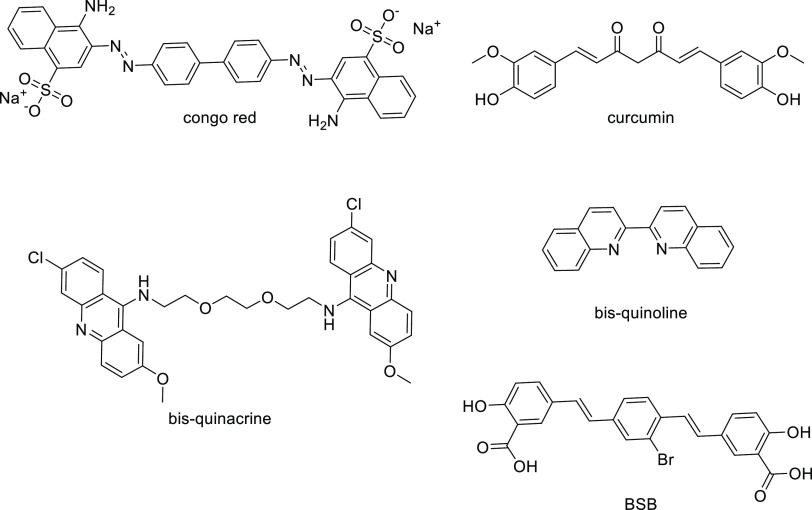
Examples of known palindromic antiaggregating compounds.

We noticed that several antiaggregating compounds share a symmetrical bifunctional structure, consisting of two identical amyloid protein recognition motifs (PRMs) joined by an appropriate spacer (Figure 1).^11^ These symmetrical compounds have been described as bivalent compounds or “palindromic compounds”,^12^ as their structure can be read in the same way in the forward or reverse direction. Considering the oligomeric and repetitive structure of fibrillar aggregates, bivalent compounds have been suggested to interact simultaneously with the two binding surfaces. Thus, bivalent ligands should cross-link fibrils and perturb the aggregation process.

**Figure 2:**
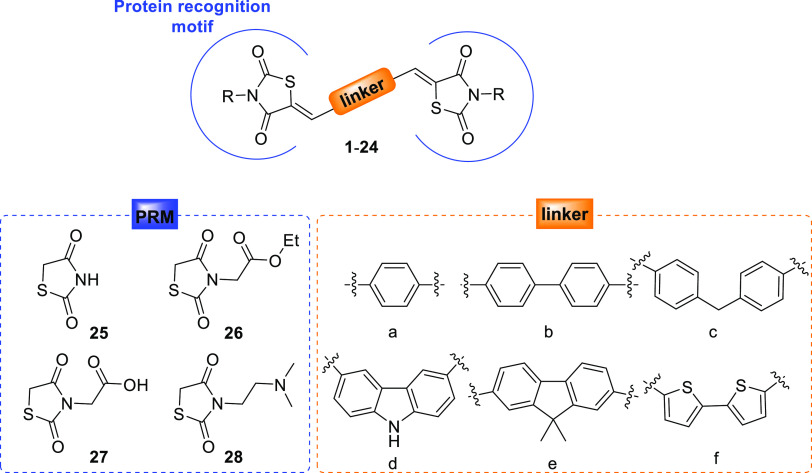
Design strategy and general structure of bivalent derivatives **1**–**24**. Two 2,4-thiazolidinedione protein recognition motifs (PRMs) (**25**–**28**) were connected via an aromatic linker (a–f).

In light of this, we focused on a bivalent strategy to identify novel MTDLs able to inhibit both Aβ and Tau aggregation processes. Herein, we describe the design and synthesis of a library of bivalent 2,4-thiazolidinedione (TZD, **25** in Figure 2) derivatives (**1**–**24**, general structure in Figure 2), together with the *in vitro*, *in cellulo*, and *in vivo* evaluation of their inhibitory activity against the aggregation of Aβ and Tau proteins.

## Results and Discussion

### Design

The design strategy for bivalent compounds **1**–**24** is illustrated in Figure 2. The selection of the TZD moiety **25** as PRM was motivated by several observations. Thanks to the presence of two H-bond acceptor and one H-bond donor groups, TZD and rhodanine scaffolds (5-arylidene substituted) have been shown by us and others to selectively recognize amyloid fibrillar structures and display promising antiaggregating properties.^13,14^ Interestingly, molecular dynamics simulations revealed critical interactions, which account for the tight binding of TZD to the Tau hexapeptide core fragment and allow disruption of the ordered structure of oligomers.^15^ Considering the role of electrostatic interactions within the architecture of amyloid fibrils,^16^ we decorated the TZD scaffold with different ionizable groups. Thus, a carboxylic acid and a secondary amine were selected as substituents for the nitrogen of the TZD scaffold (**27** and **28**). To study the impact of this functionalization on the antiaggregating activity, the corresponding nonionizable ester derivative **26** was also selected.

**Scheme 1:**
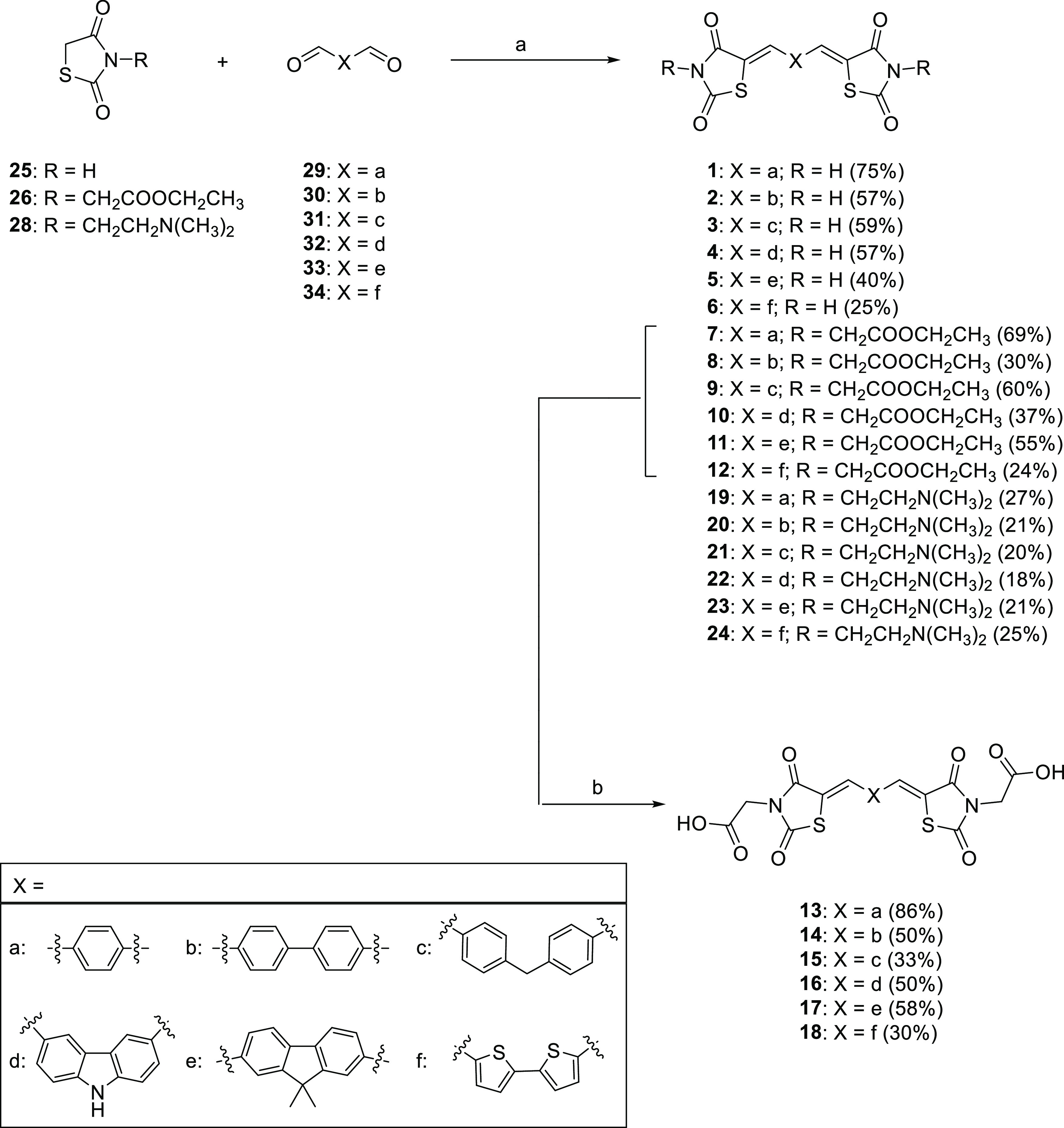
Synthetic Procedure for the Synthesis of Target Compounds **1**–**24** Reagents and Conditions: (a) EDDA, 100 °C, 45 min, MWI and (b) AcOH, HCl, 120 °C, 6 h.

After the selection of the proper PRMs, we turned our attention to suitable linkers. Several antiaggregating bivalent compounds share a common chemical structure consisting of planar, π-conjugated rings able to provide van der Waals and π–π stacking interactions.^17,18^ Thus, phenyl, biphenyl, diphenylmethane, carbazole, fluorene, and bisthiophene linkers were selected to evaluate the importance of the conjugation system for the antiaggregating activity of the bivalent compounds. Importantly, (bi)-phenyl,^19^ carbazole, and fluorene derivatives have been reported as inhibitors of Aβ aggregation,^20,21^ while bisthiophene and pentameric thiophene derivatives are well-known Aβ and Tau fluorescent probes.^22−24^ The combination of four different TZD-based PRMs with six different aromatic linkers, led to the development of a combinatorial library of 24 bivalent compounds (**1**–**24**, see Scheme 1 for individual structures).

### Chemistry

Target compounds **1**–**24** were assembled by condensing dialdehyde linkers with two PRMs via an optimized version of the Knoevenagel reaction (Scheme 1).^14^ The procedure is green, catalyzed by ethylenediamine diacetate (EDDA) under solvent-free conditions and microwave irradiation (MWI) at 100 °C, for 45 min. In detail, the unsubstituted-(**1**–**6**), ethyl ester-(**7**–**12**), and *N*-dimethylamino-derivatives (**19**–**24**) were synthesized through the condensation of TZD derivatives **25**, **26**, and **28** (3 equiv) with aromatic dialdehydes **29**–**34** (1 equiv), in the presence of EDDA (0.5 equiv). The bivalent derivatives **1**–**12** and **19**–**24** were obtained with yields varying from 18 to 75%. For the synthesis of carboxyl-derivatives (**13**–**18**), the Knoevenagel reaction was followed by acid-catalyzed hydrolysis of the corresponding ester derivatives (Scheme 1). Thus, esters **7**–**12** were refluxed in acetic acid and concentrated HCl overnight, yielding the carboxylic acids **13**–**18** in good yields (30–86%).

**Scheme 2:**
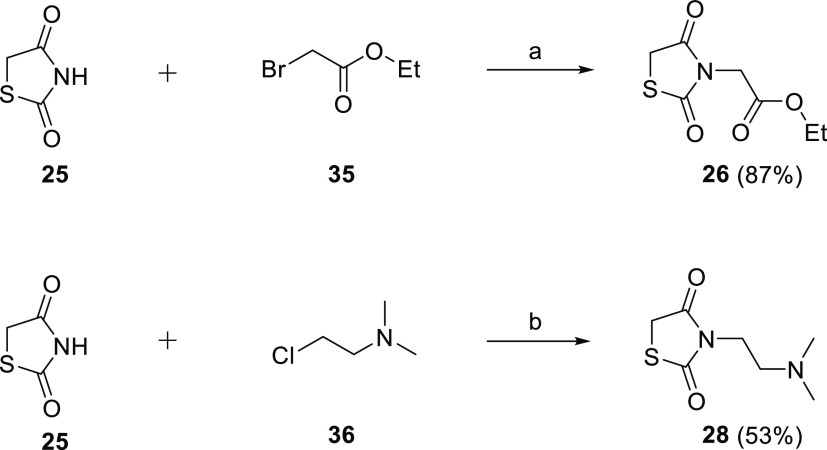
Synthetic Procedure for the Synthesis of *N*-Substituted TZD Derivatives **26** and **28** Reagents and Conditions: (a) K_2_CO_3_, acetone, 100 °C, 45 min MWI and (b) Cs_2_CO_3_, acetone, 100 °C, 45 min MWI.

The synthesis of *N*-substituted TZD intermediates was also developed. Compound **26** was obtained in good yield (87%, Scheme 2) upon *N*-alkylation of **25** with ethyl-2-bromoacetate (**35**), under MWI, in acetone at 100 °C for 45 min. The *N*-alkylation protocol for the synthesis of **28** required the use of a stronger base, i.e., Cs_2_CO_3_ instead of K_2_CO_3_ (53% yield, Scheme 2).

**Scheme 3:**
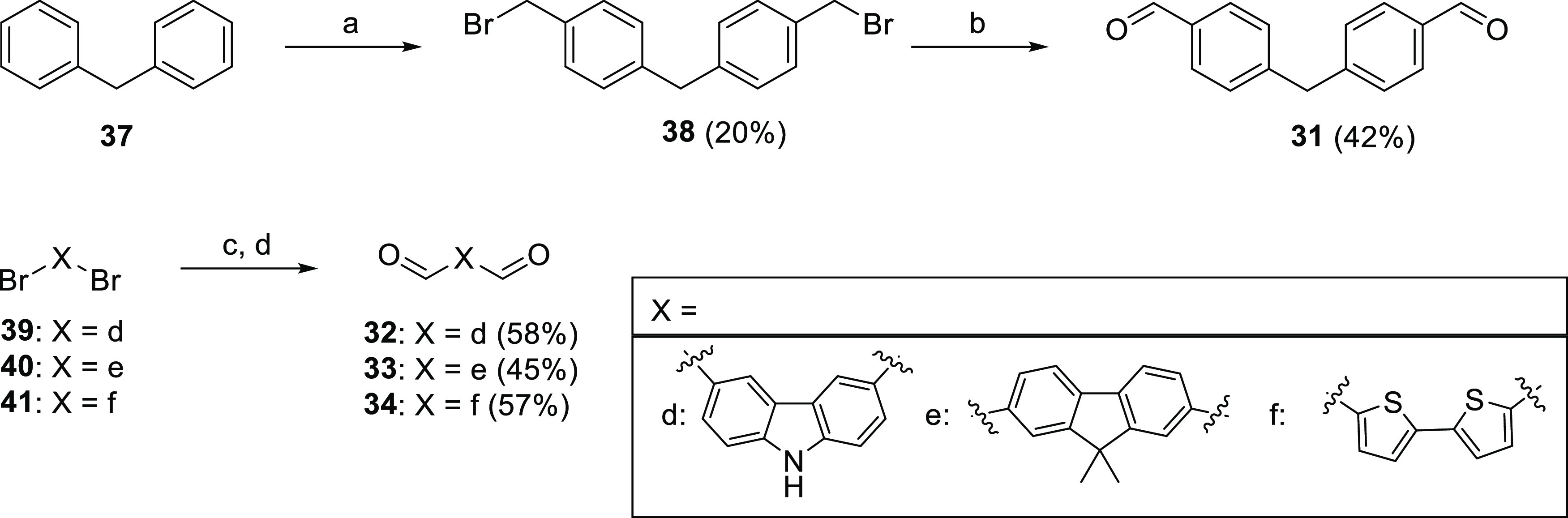
Synthetic Procedure for the Synthesis of Dialdehydes **31**–**34** Reagents and conditions: (a) formaldehyde, 33 wt % HBr in AcOH, reflux, 12 h; (b) HMTA, CHCl_3_, reflux, 6 h; and (c) AcOH, reflux, 6 h; (d) nBuLi, DMF, THF dry, −78 °C to rt, 3 h.

While dialdehyde intermediates **29** and **30** were commercially available, dialdehydes **31**–**34** were synthesized, as reported in Scheme 3. Synthesis of **31** was achieved through a two-step sequence (Scheme 3).^25^ In the first step, dibromomethylation of diphenylmethane **37** with formaldehyde and 33 wt % solution of HBr in acetic acid gave intermediate *bis*[4-(bromomethyl)phenyl]methane **38** (20%). The second step was a Sommelet reaction in which **38** was converted to **31**, using hexamethylenetetramine (HMTA), with a moderate yield (42%).^26^ The synthesis of dialdehydes **32**–**34** was achieved in moderate to good yields (45–58%) through direct lithiation of the corresponding dibromo derivatives **39**–**41** and subsequent formylation (Scheme 3).^27^ To the best of our knowledge, this is the first case in which this reaction is exploited for the synthesis of 9,9-dimethylfluorene and bisthiophene dialdehydes.

All of the final compounds **1**–**24** were characterized in terms of identity and purity (^1^H- and/or ^13^C NMR, LC-MS, HR-MS), and collected data are reported in the Experimental Section and SI.

### Aβ_42_ and Tau Antiaggregating Activity

As it would have been labor-intensive and expensive to screen such a number of compounds in two separate antiaggregating studies using recombinant Aβ and Tau, we turned our attention to a simple model of protein aggregation, namely, the intact *Escherichia coli* (*E. coli*) cells overexpressing Aβ_42_ and Tau proteins.^28,29^ Building on the fact that inclusion bodies produced by overexpression of these proteins in *E. coli* mainly consist of fibrillar aggregates, this simple screening method uses bacteria as an *in vivo* reservoir for tracking the inhibition of amyloid-type protein aggregation in real time, using the amyloid dye thioflavin S (ThS). Of note, this screening has already been used for the evaluation of libraries of antiaggregating compounds.^28−38^

All bivalent derivatives were tested at 10 μM concentration, and the obtained results are reported in Table 1. Synthetic huprine Y (a poor inhibitor of Aβ42 and Tau aggregation),^33^ HUP7TH (a dual Aβ42/Tau inhibitor),^39^ and natural anthraquinone rhein^33^ were used as control compounds (Figure S1). Generally, derivatives featuring a phenyl, biphenyl, or diphenylmethane linker showed poor antiaggregating activity, with percentages of inhibition lower than 30% for both proteins. Only the phenyl derivative **7** resulted moderately active, showing 40% inhibition for Aβ_42_ aggregation and 30% for Tau. On the other side, carbazole and 9,9-dimethylfluorene derivatives showed more promising results. Carbazoles **10** and **16**, carrying, respectively, an ester or a carboxylic acid appendage, showed moderate activity (% inhibition between 30 and 40%), while the *N,N*-dimethylaminoethyl derivative **22** resulted the most potent compound of the series. Compound **22** showed 74.0% inhibition of Aβ_42_ aggregation and 66.1% inhibition of Tau aggregation. 9,9-dimethylfluorene derivatives **17** (acetic acid) and **23** (*N,N*-dimethylaminoethyl) showed an interesting activity on both aggregation processes, with % of inhibition higher than 50 and 40%, respectively.

**Table 1:** Inhibitory Activity of Bivalent Derivatives **1**–**24** toward Aβ_42_ and Tau Aggregation, Together with Neurotoxicity Data on Differentiated CGNs (24 h Treatment)

cmp	linker	R	% inhibition Aβ_42_ aggregation @ 10 μM^a^	% inhibition Tau aggregation @ 10 μM^a^	% survival neurotoxicity @ 10 μM^b^
1	phenyl	-H	25.4 ± 1.9	19.4 ± 3.6	77.6 ± 6.1
2	biphenyl	-H	20.2 ± 2.1	16.5 ± 3.1	81.3 ± 6.5
3	diphenylmethane	-H	12.9 ± 3.4	5.2 ± 2.7	62.4 ± 9.6
4	carbazole	-H	24.7 ± 4.1	21.2 ± 2.6	73.4 ± 5.1
5	9,9-dimethylfluorene	-H	23.6 ± 2.6	14.8 ± 3.0	67.9 ± 5.4
6	bisthiophene	-H	42.4 ± 4.5	19.4 ± 3.9	89.9 ± 1.2
7	phenyl	-CH_2_COOCH_2_CH_3_	40.7 ± 4.4	30.6 ± 3.6	90.1 ± 2.1
8	biphenyl	-CH_2_COOCH_2_CH_3_	14.3 ± 3.5	18.1 ± 3.2	87.2 ± 3.1
9	diphenylmethane	-CH_2_COOCH_2_CH_3_	14.5 ± 4.1	19.9 ± 3.6	88.7 ± 4.1
10	carbazole	-CH_2_COOCH_2_CH_3_	31.9 ± 3.0	37.7 ± 4.1	84.2 ± 6.3
11	9,9-dimethylfluorene	-CH_2_COOCH_2_CH_3_	17.5 ± 4.5	33.1 ± 4.5	85.8 ± 6.9
12	bisthiophene	-CH_2_COOCH_2_CH_3_	7.0 ± 3.7	9.2 ± 5.0	90.2 ± 2.1
13	phenyl	-CH_2_COOH	16.1 ± 3.9	25.3 ± 4.2	82.3 ± 2.9
14	biphenyl	-CH_2_COOH	35.1 ± 1.9	27.1 ± 2.5	89.0 ± 2.2
15	diphenylmethane	-CH_2_COOH	26.2 ± 1.9	3.8 ± 5.2	90.9 ± 1.5
16	carbazole	-CH_2_COOH	32.1 ± 3.1	37.9 ± 3.5	90.0 ± 2.8
17	9,9-dimethylfluorene	-CH_2_COOH	51.4 ± 2.7	55.6 ± 3.2	89.3 ± 2.3
18	bisthiophene	-CH_2_COOH	65.6 ± 3.3	15.8 ± 4.7	90.9 ± 3.1
19	phenyl	-CH_2_CH_2_N(CH_3_)_2_	8.1 ± 4.4	19.4 ± 3.6	89.4 ± 2.0
20	biphenyl	-CH_2_CH_2_N(CH_3_)_2_	26.4 ± 4.2	34.9 ± 1.9	91.1 ± 0.9
21	diphenylmethane	-CH_2_CH_2_N(CH_3_)_2_	16.1 ± 3.8	38.7 ± 3.3	91.1 ± 1.5
22	carbazole	-CH_2_CH_2_N(CH_3_)_2_	74.0 ± 4.3	66.1 ± 3.9	94.3 ± 1.8
23	9,9-dimethylfluorene	-CH_2_CH_2_N(CH_3_)_2_	44.8 ± 4.7	41.9 ± 3.9	87.9 ± 3.4
24	bisthiophene	-CH_2_CH_2_N(CH_3_)_2_	20.3 ± 4.8	17.3 ± 4.5	88.8 ± 2.3
Huprine Y			4.6 ± 2.3	*n.d.*	*n.d.*
HUP7TH			77.6 ± 1.5	69.5 ± 1.6	*n.d.*
Rhein			*n.d.*	39.2 ± 2.1	*n.d.*

a Inhibition of Aβ_42_ and Tau aggregation in intact *E. coli* cells upon treatment with a compound concentration of 10 μM. Values are expressed as the mean ± SEM of four independent experiments.

b Viability of cerebellar granule neurons (CGNs) after 24 h of incubation with the test compound at a 10 μM concentration. The results are expressed as a percentage of healthy nuclei on the total nuclei counting number, after Hoechst staining, and are the mean ± SEM of three different experiments; n.d. not determined.

Interestingly, a different behavior was observed for the bisthiophene derivatives. Indeed, while all the other analogues showed almost comparable activity in inhibiting the aggregation of both Aβ_42_ and Tau proteins, bisthiophene derivatives showed selectivity toward Aβ_42_ compared to Tau. This selectivity is exemplified by compounds **6** and **18**. Indeed, both resulted poor inhibitors of Tau aggregation (inhibition < 20%), while showing interesting potency toward Aβ_42_, with a remarkable 65.6% inhibition shown by **18**.

Looking at the most active compounds (**7**, **10**, **16**–**18**, **22**, and **23**), it appears that the decoration of the PRM had an impact on the antiaggregating profile of these compounds. Indeed, the addition of both ionizable (compare **16**–**18***vs***4–6**) and nonionizable (**7***vs***1**, **10***vs***4**) moieties on the TZD fragment led to more potent compounds. The most striking effect was for carbazole **22**, where the introduction of a protonatable group increased the activity from 24.7 and 21.2% (for unsubstituted **4)** to 74.0 and 66.1%, respectively. Of note, although the most potent compounds have two groups that should be mostly ionized at physiological pH, they are effectively internalized within *E. coli* cells. Thus, it might be anticipated that permeation across the two-membrane bacterial cell envelope, which might have reduced their effective concentrations in *E. coli* and, hence, their activities, should not be an issue for these compounds.

### Toxicity in Cerebellar Granule Neurons

In parallel with the evaluation of the antiaggregating potential, we assessed the neurotoxicity of all bivalent derivatives (**1**–**24**) on rat primary cultures of cerebellar granule neurons (CGNs), by healthy *vs* total nuclei counting after Hoechst staining. In this way, we aimed to remove potentially toxic compounds from consideration early in the screening process. CGNs were established a few decades ago, and since then have become one of the most useful *in vitro* models to study neuronal death.^40^ The compounds were tested at 10 μM concentration after 24 h treatment, and results are reported in Table 1.

Generally, all of the compounds were well tolerated, with 17 analogues out of 24 showing cell viability higher than 85%. Interestingly, the only four compounds (**1, 3**–**5**) showing some neurotoxic effect (cell viability < 80%), shared the unsubstituted TZD moiety. Thus, the addition of the appendage moiety on TZD seems favorable also in terms of potential neurotoxic effects.

Both the collected Aβ and Tau antiaggregating activities and neurotoxicity data were considered for compound progression. Particularly, requirements for further evaluation were (i) lack of neurotoxicity and (ii) inhibition of both Aβ and Tau aggregation higher than 30%. Thus, compounds **7**, **10**, **16**, **17**, **22**, and **23** progressed to the next assay.

### Blood–Brain Barrier Permeability Prediction

One of the main problems in developing a CNS-active compound relies on the ability of such a compound to permeate the blood–brain barrier (BBB) at its therapeutic concentration. Thus, to reduce attrition in the development process, the evaluation of BBB penetration at a very early drug discovery stage is of crucial importance. In light of this, the ability of the previously selected compounds to cross the BBB was predicted by a parallel artificial membrane permeability assay (PAMPA)-BBB,^41^ a high-throughput technique widely used as an indicator of a molecule’s passive diffusion through the BBB. Six commercially available drugs were used to validate the assay. A good correlation between reported and experimentally described values were obtained (see Supporting Information, Table S1). Thus, in accordance with data from the literature, compounds presenting an effective permeability (Pe) > 4.0 × 10^-6^ cm/s were classified as CNS permeable (CNS+). Based on this, the acetic acid derivatives **16** and **17** were classified as CNS-. However, as a very encouraging result, the ethyl esters **7** and **10**, and the dimethylamino analogues **22** and **23** were predicted to passively diffuse across the BBB. Notably, 9,9-dimethylfluorene **23** showed a Pe value similar to those of standard AD drugs donepezil and rivastigmine (Table S1).

### Interaction with Aβ- and Tau-Isolated Proteins

After assessing the potential of the compounds to cross the BBB, we wanted to investigate the ability of the two most active and BBB-permeable compounds, **22** and **23**, to interfere with the Aβ_42_ and Tau aggregation process *in vitro*, using isolated peptides and previously developed/validated aggregation assays.^42^ These assays allow us to indirectly monitor fibril formation, thanks to changes in the emission intensity of the fluorescent dye ThT when aggregates are formed.^42^ The achievement of reproducible and trustable data requires the final amount of organic solvents, and solubility issues are carefully prechecked.^43^ Hence, to assess antiaggregation properties toward Aβ_42_, these aspects were investigated in dedicated solubility assays. The results showed that compound solubility in the assay condition was too limited to grant trustable results, thus preventing their evaluation on the commonly used ThT-based assay.

The interaction of a fluorescent compound with three-dimensionally organized Aβ_42_ fibrils may lead to a change in its fluorescent properties as a consequence of either a reduction of flexibility due to multiple-interaction points and/or of a change in compound–solvent interactions because of the insertion of the compound within fibril helices. Hence, a change in the spectral properties of a fluorescent compound potentially able to bind Aβ_42_ protofibrils/fibrils can be used as indirect proof that the two entities (amyloid and ligand) interact. This assay setup allows the use of a slightly higher amount of DMSO, which is sufficient to grant compound solubility, because addition of the tested compound to Aβ_42_ samples is performed when fibrillization has already occurred.

**Figure 3:**
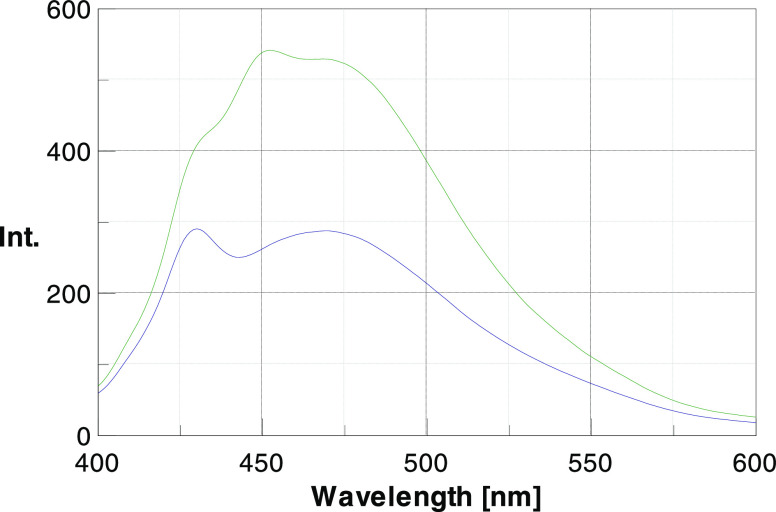
Fluorescence emission spectra of **23** (0.375 μM) in Gly–NaOH buffer (50 mM, pH 8.5) in the absence (blue line) and presence (green line) of preaggregated Aβ_42_ (0.75 μM). [Aβ_42_]/[**23**] = 2/1. λ_exc_ = 374 nm.

Thus, we recorded the emission spectra of compound **23** in the absence and presence of preaggregated Aβ_42_. While the excitation/emission maxima for the bound and unbound **23** did not significantly differ (i.e., λ_exc_ = 374 nm; λ_em_ maximum = 473 nm), a strong hyperchromic effect upon binding was observed, thus providing a proof of the interaction between the compound and the aggregated protein (Figure 3).

**Figure 4:**
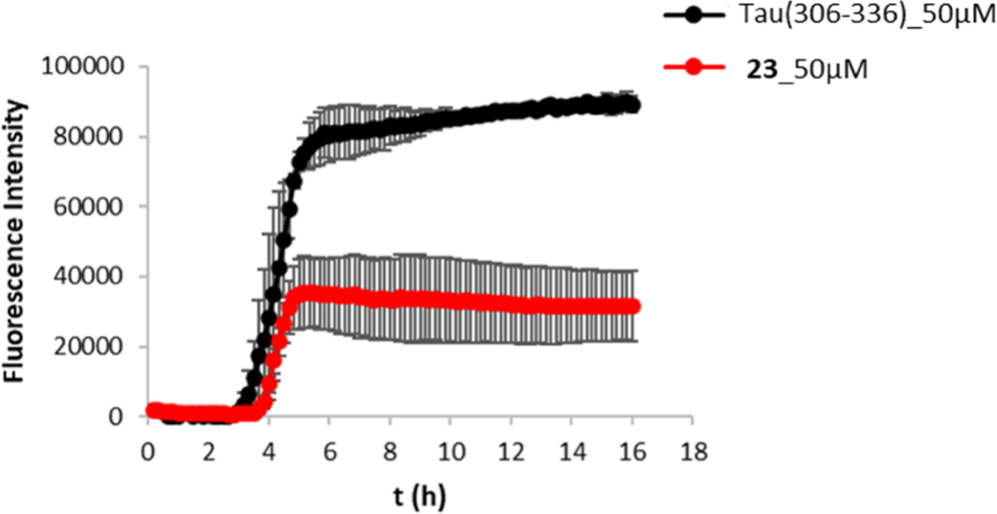
Inhibition of Tau peptide_(306–336)_ aggregation (50 μM) by compound **23** (50 μM). Overlaid trends of the fluorescence intensity (λ_exc_ = 446 nm, λ_em_ = 490 nm) over 16-h incubation for the Tau peptide_(306–336)_ alone (black line) and in the presence of **23** (red line) in a 1/1 ratio. ThT was used as a fluorescent dye. Data are the mean of at least two independent experiments, each performed in duplicate.

Secondly, we investigated the ability of the compounds to interfere with Tau aggregation, using the third repeat domain Tau fragment (Tau_(306-336)_ peptide).^44^ The aggregation profiles of the Tau_(306–336)_ peptide (50 μM) in the absence and presence of equimolar concentrations of either **22** or **23** were monitored in phosphate buffer 50 mM, pH 7.4, using Thioflavin T (ThT) as the detection dye. Unfortunately, under this experimental setup, solubility issues arose for derivative **22**, which prevented us to obtain reliable trends. Conversely, compound **23** showed a strong antiaggregating activity (51.8 ± 11.7% inhibition), resulting only slightly less potent than the known Tau inhibitor doxycycline (61.5 ± 0.8% inhibition) (Figure 4 and Table S2).^45^ Though, it was not able to significantly delay the oligomerization phase (lag phases in the absence and presence of the inhibitor only slightly differ) and some dye displacement might contribute to some extent to a lower fluorescent signal.^45^

Taken collectively, the data from *E. coli*, PAMPA, neurotoxicity, and aggregation assays were considered encouraging enough to further explore the potential of our bivalent compounds **22** and **23** in an *in vivo* AD model.

### Activity Profile in a *Drosophila melanogaster* Model of AD

*Drosophila melanogaster* is a powerful *in vivo* model for the initial screening of AD drug candidates.^46−49^ In the last years, several transgenic flies expressing human Aβ_42_ and Tau proteins have been developed, and they are now providing new insights into disease mechanisms, as well as assisting in the identification of novel AD drugs.^50−55^ Specifically, expression of the Arctic mutant (Glu22Gly) Aβ_42_ in *Drosophila* neural tissue favors oligomer formation and results in different symptoms reminiscent of AD, including defective locomotion and memory, as well as markedly reduced longevity. Moreover, fly brains display characteristic amyloid plaques and amyloid pathology.^56−58^ Based on the good correlation between Aβ aggregation and the severity of the various AD phenotypes, we used Arctic Aβ_42_*Drosophila* flies to preliminarily test the effect of our bivalent derivatives **22** and **23**.

**Figure 5:**
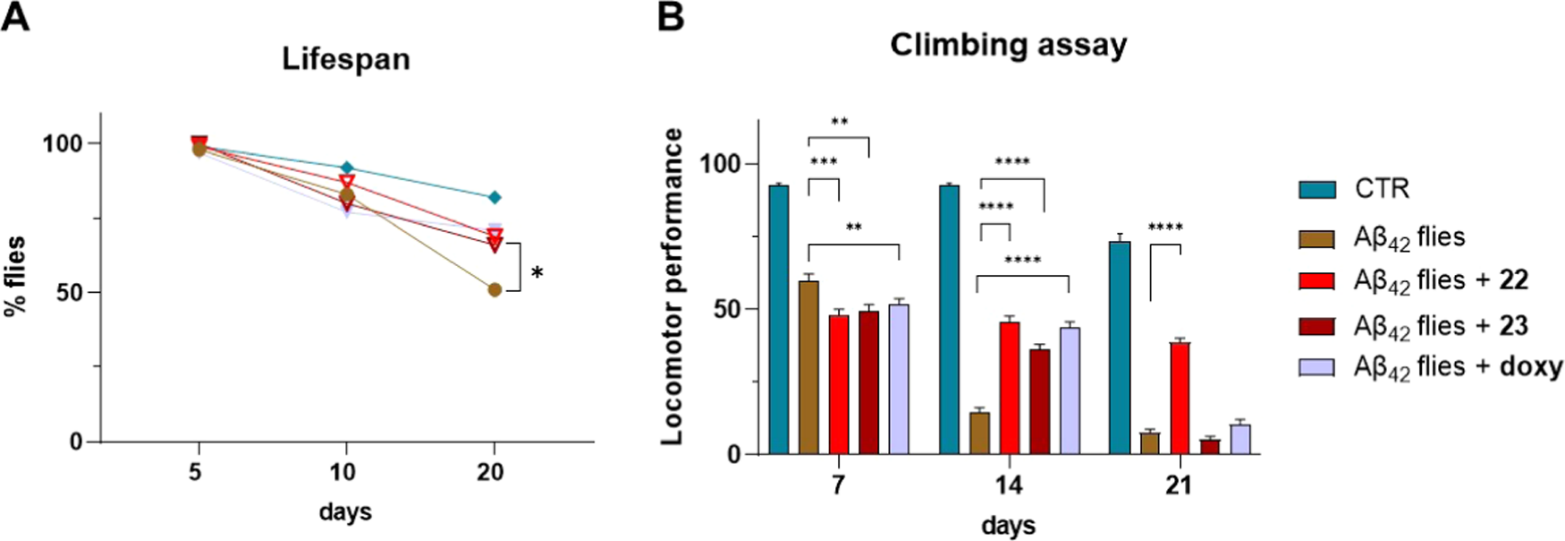
(A) Lifespan analysis comparing treated and untreated Aβ_42_*Drosophila* flies, together with w^1118^ flies as control (CTR flies), on days 5, 10, and 20 post-eclosion (*p-value < 0.05). (B) Behavioral test measuring the climbing abilities of treated and untreated Aβ_42_*Drosophila* flies, together with w^1118^ flies as control (CTR flies), on days 7, 14, and 21 post-eclosion. Values are expressed as the mean ± SEM. Unpaired t-test resulted statistically different (^**^p-value < 0.005; ^***^p-value < 0.001; and ^****^p-value < 0.0001 compared to Aβ_42_ flies).

As longevity is a phenotype that can be rapidly measured in this *in vivo* AD model, we first assessed changes in *Drosophila’*s lifespan with and without treatment with **22** and **23** (20 μM). We screened different concentrations, from 10 to 100 μM, to assess the most effective dose. At the concentration of 20 μM, we obtained the best results in combination with a low toxicity rate. We again used as control the antibiotic doxycycline, which is capable of halting amyloid aggregation of several disease-associated proteins (including Aβ and Tau).^45^ Doxycycline was tested at 50 μM, a concentration used in similar previous experiments.^59^ As shown in Figure 5A, after 20 days, only 50% of Aβ_42_ flies were still alive. Importantly, treatment with both **22** and **23** resulted in an increase in longevity, being comparable to that of the reference compound doxycycline (at a higher concentration).

Locomotor effects in Aβ_42_*Drosophila* flies are clearly associated with Aβ_42_ overexpression and in particular, climbing is a strong and reproducible behavior.^56^ Thus, we performed behavioral tests to assess the climbing abilities of Aβ_42_*Drosophila* flies with and without treatment with **22** (20 μM), **23** (20 μM), and doxycycline (50 μM), compared to control flies. As shown in Figure 5B, Aβ_42_*Drosophila* flies showed an important decline in their climbing ability compared to control flies, being almost immobile after day 21. Compound **23** showed a promising effect, with a partially recovered phenotype that lasted until day 14. Unfortunately, the effect was not sustained further, as there was no difference between the treated and untreated flies on day 21. On the other hand, treatment with **22** showed extremely promising results. Indeed, the positive effect of **22** was sustained for all of the different time points, greatly improving the climbing performances of Aβ_42_*Drosophila* flies. Importantly, **22** resulted to be even more active than doxycycline. On day 21, good climbing performances of flies treated with **22** persisted, while they strongly declined in doxycycline-treated flies. We should again remark that the testing concentration for **22** was lower (20 μM) compared to doxycycline (50 μM). Finally, data collected on *Drosophila* nicely reflected the antiaggregating results obtained in *E. coli*, with carbazole **22** being more active than 9,9-dimethylfluorene **23**.

**Figure 6:**
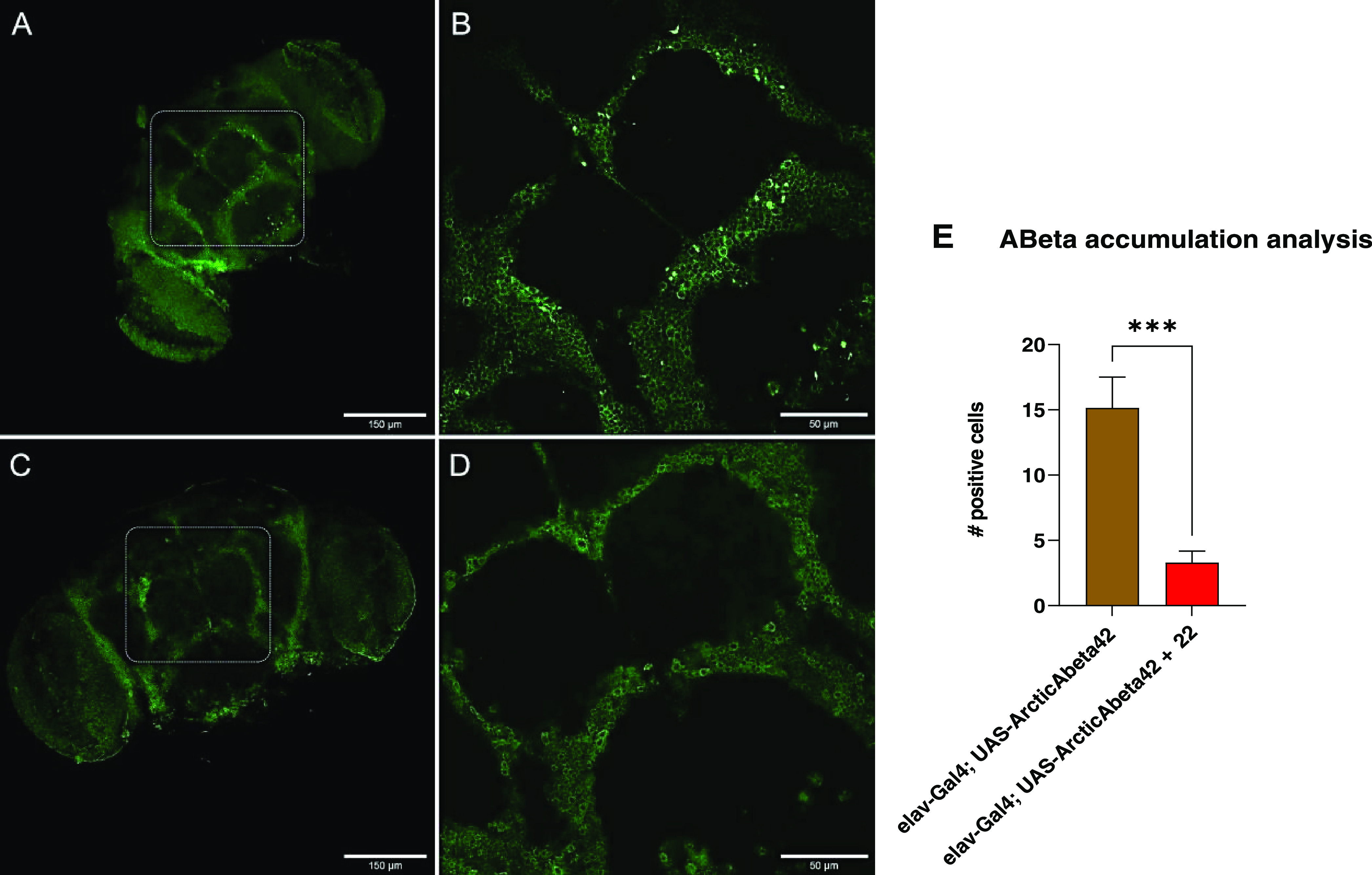
Immunofluorescence staining of Aβ_42_ aggregates within flies’ brains. (A) Untreated Aβ_42_*Drosophila* flies, showing Aβ_42_ aggregates visible as green deposits. (B) Magnification of the selected area from A, showing Aβ_42_ aggregates. (C) Aβ_42_*Drosophila* flies treated with **22** showed almost the absence of Aβ_42_ aggregates. (D) Magnification of the selected area from C. Scale bar: 150 μm in panels (A) and (C); 50 μm in panels (B) and (D). (E) Quantification analysis of Aβ_42_ accumulation in Aβ_42_*Drosophila* flies’ brains (n=8 each genotype). Unpaired *t*-test comparing untreated and treated flies resulted statistically different (^***^p < 0.001).

Motivated by these positive results, we decided to study the ability of the most promising **22** to reduce the presence of amyloid plaques and aggregates in the brain of Aβ_42_ flies. Indeed, these flies are characterized by intracellular Aβ_42_ accumulation, followed by non-amyloid aggregates that resemble diffuse plaques.^56^ Thus, the brains of treated and untreated flies, together with the control, were dissected and analyzed at 15 days post-hatching. Confocal microscopy of untreated Aβ_42_*Drosophila* flies showed diffuse peptide aggregates distributed throughout the brain, especially within the *Drosophila* mushroom body (Figure 6A,B). Importantly, such deposits were nearly absent in the brains of Aβ_42_*Drosophila* flies treated with **22** (Figure 6C,D). Quantification analysis showed an 80% reduction of aggregates in Aβ_42_*Drosophila* adult brains treated with carbazole **22**, compared with untreated ones (Figure 6E). This result might confirm that the increased life span and locomotive ability were linked to a direct antiaggregating effect.

In addition, the observed *Drosophila* profile might be encouraging because after administration in the fly food, **22** is able to exert its effect on Aβ_42_ expressed in the CNS of a whole organism.

## Conclusions

The complex nature of AD, together with a still poor understanding of its pathophysiological mechanisms, seems to be responsible for the lack of effective treatments in the clinic. However, several studies are now shedding light on the interconnected role of two pathognomonic proteins in AD: Aβ and Tau. Available evidence suggests these proteins can act synergistically, causing synaptic dysfunction, neuronal loss, and behavioral deficits. In light of this, we decided to apply an MTDL approach directed to both these protein aggregates in AD. We believe that the development of a dual Aβ/Tau aggregation inhibitor should be more effective, compared to existing single-target treatments (*e.g.*, aducanumab). Thus, we presented here the design and synthesis of a focused library of 24 bivalent TZD derivatives (**1**–**24**), aiming to inhibit both Aβ_42_ and Tau protein aggregation. The most promising compound from this series is the carbazole derivative **22**, which showed positive antiaggregating results in intact *E. coli* cells overexpressing Aβ_42_ and Tau proteins, no neurotoxicity in primary CGNs, and BBB permeability in a PAMPA–BBB assay. Moreover, compound **22** ameliorated AD-like phenotypes in a transgenic *Drosophila melanogaster* model of Alzheimer’s disease/Aβ toxicity, being even more effective than the dual inhibitor doxycycline. Carbazole **22** was able not only to improve the lifespan and climbing abilities of Aβ_42_ expressing flies but also to reduce the presence of amyloid aggregates in their brains. It is important to highlight, however, that the poor solubility of compound **22** prevented us from studying in more depth its *in vitro* interaction with the isolated proteins.

In this respect, we cannot exclude that the mechanism of amyloid aggregation modulation by **22** may occur by its self-assembly into aggregates and consequent interactions with the aggregating protein^60,61^ in a manner characteristic of colloidal inhibition. This is a potential mechanism of many compounds inhibiting aggregation of diverse amyloid-type proteins.^60^

In conclusion, this new class of dual antiaggregating compounds could represent a promising starting point for the design of the second generation of analogues, where improved solubility and elucidation of the molecular mechanism of action would be key needed steps to allow compounds to be progressed for further studies.

## Experimental Section

### Chemistry

All of the commercially available reagents and solvents were purchased from Sigma-Aldrich, Alpha Aesar, and VWR, and used without further purification. Reactions were followed by analytical thin-layer chromatography (TLC), on precoated TLC plates (layer 0.20 mm silica gel 60 with a fluorescent indicator UV254, from Sigma-Aldrich). Developed plates were air-dried and analyzed under a UV lamp (UV 254/365 nm). A CEM Discover SP-focused microwave reactor was used for microwave-mediated reactions. All melting points (m.p.) were determined in open glass capillary tubes using a BÜCHI Melting Point B-540 apparatus. Nuclear magnetic resonance (NMR) experiments were run on a Varian VXR 400 (400 MHz for ^1^H, 100 MHz for ^13^C). ^1^H and ^13^C NMR spectra were acquired at 300 K using deuterated dimethyl sulfoxide (DMSO-*d*_6_), chloroform (CDCl_3_), or trifluoroacetic acid (TFA-*d*_1_) as solvents. Chemical shifts (δ) are reported in parts per million (ppm) relative to tetramethylsilane (TMS) as the internal reference, and coupling constants (*J*) are reported in hertz (Hz). The spin multiplicities are reported as s (singlet), br s (broad singlet), d (doublet), t (triplet), q (quartet), and m (multiplet). Exchangeable NH or OH protons were not observed in the ^1^H spectra of **1**, **3**, **15**, **17**, and **18**. Low-resolution and high-resolution mass spectra were recorded on a VG707EH-F or a Xevo G2-XS QTof apparatus, and electrospray ionization (ESI) both in positive and negative modes was applied. All the final compounds showed ≥95% purity by analytical HPLC. Compounds were named following IUPAC rules as applied by ChemBioDraw Ultra (version 14.0). As already reported,^62^ final compounds **1**–**24** were obtained as single Z isomers. Indeed, their ^1^H NMR spectra show only one signal attributable to the resonance of the 5-methylidene proton in the range 7.50–8.70 ppm, while in their ^13^C NMR spectra the 5-methylidene carbon and the C5 of the TZD ring resonated in the ranges 130.5–140.7 and 117.5–128.0 ppm, respectively.

#### General Procedure for the Synthesis of Compounds **1–12** and **19–24**

The corresponding dialdehydes (1 mmol) reacted with the corresponding TZD derivative (3 mmol), using EDDA (0.5 mmol) under microwave irradiation at 100 °C for 45 min. The reaction mixture was diluted with water and the solid was collected by filtration. After washing the solid with water, all of the final compounds were purified through crystallization or column chromatography. For compounds **1**, **6**, and **18** only ^1^H NMR spectra were recorded, due to their extremely low solubility.

##### (5Z,5′Z)-5,5′-(1,4-Phenylenebis(methanylylidene))bis(thiazolidine-2,4-dione) (**1**)

The title compound **1** was obtained as a yellow solid, according to the general procedure using **25** and dialdehyde **29**. Yield 75%. m.p. 236 °C (dec.); ^1^H NMR (400 MHz, DMSO-*d*_6_): δ 7.79 (s, 2H); 7.72 (s, 4H). MS (ESI^–^) *m*/z for C_14_H_8_N_2_O_4_S_2_: 331 [M – H]^−^. HR-MS calcd for C_14_H_7_N_2_O_4_S_2_ 330.9853, found 330.9860 [M – H]^−^.

##### (5Z,5′Z)-5,5′-([1,1′-Biphenyl]-4,4′-diylbis(methanylylidene))bis(thiazolidine-2,4-dione) (**2**)

The title compound **2** was obtained as a yellow solid, according to the general procedure using **25** and dialdehyde **30**. Yield 57%. m.p. 249 °C (dec.); ^1^H NMR (400 MHz, DMSO-*d*_6_): δ 12.63 (br s, 2H); 7.93 (d, *J* = 8.1, 4H); 7.84 (s, 2H); 7.71 (d, *J* = 8.1, 4H). ^13^C NMR (100 MHz, DMSO-*d*_6_): δ 168.3; 168.0; 140.7; 133.3; 131.4; 131.2; 128.0; 124.5 (δ 162.73, 36.2, 31.2 residual DMF from recrystallization). MS (ESI^–^) *m*/z for C_20_H_12_N_2_O_4_S_2_: 407 [M – H]^−^.

##### (5Z,5′Z)-5,5′-((Methylenebis(4,1-phenylene))bis(methanylylidene))bis(thiazolidine-2,4-dione) (**3**)

The title compound **3** was obtained as a white solid, according to the general procedure using **25** and dialdehyde **31**. Yield 59%. m.p. 221 °C (dec.); ^1^H NMR (400 MHz, DMSO-*d*_6_): δ 7.71 (s, 2H); 7.53 (d, *J* = 8.1, 4H); 7.40 (d, *J* = 8.1, 4H); 4.06 (s, 2H). ^13^C NMR (100 MHz, DMSO-*d*_6_): δ 168.9; 168.8; 143.6; 131.7; 131.3; 130.7; 130.1; 41.1; one carbon missing from the aromatic region. MS (ESI^–^) *m*/z for C_21_H_14_N_2_O_4_S_2_: 421 [M – H]^−^.

##### (5Z,5′Z)-5,5′-((9H-Carbazole-3,6-diyl)bis(methanylylidene))bis(thiazolidine-2,4-dione) (**4**)

The title compound **4** was obtained as a dark yellow solid, according to the general procedure using **25** and dialdehyde **32**. Yield 57%. m.p. 227 °C (dec.); ^1^H NMR (400 MHz, DMSO-*d*_6_): δ 12.43 (br s, 2H); 12.07 (s, 1H); 8.48 (s, 2H); 7.97 (s, 2H); 7.68 (s, 4H). ^13^C NMR (100 MHz, DMSO-*d*_6_): δ 168.8; 168.2; 141.8; 133.5; 128.8; 125.1; 124.2; 123.3; 120.5; 112.9. MS (ESI^–^) *m*/z for C_20_H_11_N_3_O_4_S_2_: 420 [M – H]^−^.

##### (5Z,5′Z)-5,5′-((9,9-Dimethyl-9H-fluorene-2,7-diyl)bis(methanylylidene))bis(thiazolidine-2,4-dione) (**5**)

The title compound **5** was obtained as a dark yellow solid, according to the general procedure using **25** and dialdehyde **33**. Yield 40%. m.p. 231 °C (dec.); ^1^H NMR (400 MHz, DMSO-*d*_6_): δ 12.57 (br s, 2H); 8.06 (d, *J* = 8.0, 2H); 7.86 (s, 2H); 7.80 (s, 2H); 7.63 (dd, *J* = 8.0, 1.2, 2H); 1.51 (s, 6H). ^13^C NMR (100 MHz, DMSO-*d*_6_): δ 168.5; 168.2; 155.3; 140.0; 133.6; 132.1; 129.9; 125.0; 124.1; 122.2; 47.2; 26.9. MS (ESI^–^) *m*/z for C_23_H_16_N_2_O_4_S_2_: 447 [M – H]^−^.

##### (5Z,5′Z)-5,5′-([2,2′-Bithiophene]-5,5′-diylbis(methanylylidene))bis(thiazolidine-2,4-dione) (**6**)

The title compound **6** was obtained as a red solid, according to the general procedure using **25** and dialdehyde **34**. Yield 25%. m.p. 260 °C (dec.); ^1^H NMR (400 MHz, DMSO-*d*_6_): δ 12.62 (br s, 2H); 8.03 (s, 2H); 7.68 (s, 4H); (δ 7.95, 2.88, 2.73 residual DMF from recrystallization). MS (ESI^–^) *m*/z for C_16_H_8_N_2_O_4_S_4_: 419 [M – H]^−^. HR-MS calcd for C_16_H_7_N_2_O_4_S_4_ 418.9294, found 418.9288 [M – H]^−^.

##### Diethyl 2,2′-((5Z,5′Z)-(1,4-phenylenebis(methanylylidene))bis(2,4-dioxothiazolidin-3-yl-5-ylidene))diacetate (**7**)

The title compound **7** was obtained as a yellow solid, according to the general procedure using **26** and dialdehyde **29**. Yield 69%. m.p. 249 °C; ^1^H NMR (400 MHz, DMSO-*d*_6_): δ 8.05 (s, 2H); 7.83 (s, 4H); 4.52 (s, 4H); 4.19 (q, *J* = 7.1, 4H); 1.22 (t, *J* = 7.1, 6H). ^13^C NMR (100 MHz, CDCl_3_): δ 166.8; 166.0; 165.3; 134.9; 132.7; 130.8; 123.2; 62.2; 42.2; 14.1. MS (ESI^+^) *m*/z for C_22_H_20_N_2_O8S_2_: 527 [M + Na]^+^.

##### Diethyl 2,2′-((5Z,5′Z)-([1,1′-biphenyl]-4,4′-diylbis(methanylylidene))bis(2,4-dioxothiazolidin-3-yl-5-ylidene))diacetate (**8**)

The title compound **8** was obtained as a yellow solid, according to the general procedure using **26** and dialdehyde **30**. Yield 30%. m.p. 288 °C (dec.); ^1^H NMR (400 MHz, CDCl_3_): δ 7.97 (s, 2H); 7.76 (d, *J* = 8.3, 4H); 7.63 (d, *J* = 8.3, 4H); 4.50 (s, 4H); 4.26 (q, *J* = 7.1, 4H); 1.31 (t, *J* = 7.1, 6H). ^13^C NMR (100 MHz, CDCl_3_): δ 167.2; 166.2; 165.5; 141.6; 133.7; 132.8; 130.9; 127.8; 121.4; 62.2; 42.2; 14.1. MS (ESI^+^) *m*/z for C_28_H_24_N_2_O_8_S_2_: 603 [M + Na]^+^.

##### Diethyl 2,2′-((5Z,5′Z)-((methylenebis(4,1-phenylene))bis(methanylylidene))bis(2,4-dioxothiazolidin-3-yl-5-ylidene))diacetate (**9**)

The title compound **9** was obtained as a white solid, according to the general procedure using **26** and dialdehyde **31**. Yield 60%. m.p. 214 °C; ^1^H NMR (400 MHz, CDCl_3_): δ 7.91 (s, 2H); 7.47 (d, *J* = 8.1, 4H); 7.31 (d, *J* = 8.1, 4H); 4.47 (s, 4H); 4.24 (q, *J* = 7.1, 4H); 4.08 (s, 2H); 1.30 (t, *J* = 7.1, 6H). ^13^C NMR (100 MHz, CDCl_3_): δ 167.3; 166.2; 165.5; 143.2; 134.2; 131.4; 130.7; 129.8; 120.6; 62.1; 42.1; 41.7; 14.0. MS (ESI^+^) *m*/z for C_29_H_26_N_2_O_8_S_2_: 617 [M + Na]^+^.

##### Diethyl 2,2′-((5Z,5′Z)-((9H-carbazole-3,6-diyl)bis(methanylylidene))bis(2,4-dioxothiazolidin-3-yl-5-ylidene))diacetate (**10**)

The title compound **10** was obtained as a yellow solid, according to the general procedure using **26** and dialdehyde **32**. Yield 37%. m.p. 246 °C; ^1^H NMR (400 MHz, CDCl_3_): δ 9.08 (br s, 1H); 7.98 (s, 2H); 7.94 (s, 2H); 7.62-7.43 (m, 4H); 4.52 (s, 4H); 4.31 (q, *J* = 7.1, 4H); 1.35 (t, *J* = 7.1, 6H). ^13^C NMR (100 MHz, CDCl_3_): δ 167.5; 166.8; 165.6; 141.2; 135.4; 129.3; 125.5; 123.5; 123.2; 117.7; 112.0; 62.3; 42.1; 14.1. MS (ESI^+^) *m*/z for C_28_H_23_N_3_O_8_S_2_: 616 [M + Na]^+^.

##### Diethyl 2,2′-((5Z,5′Z)-((9,9-dimethyl-9H-fluorene-2,7-diyl)bis(methanylylidene))bis(2,4-dioxothiazolidin-3-yl-5-ylidene))diacetate (**11**)

The title compound **11** was obtained as a yellow solid, according to the general procedure using **26** and dialdehyde **33**. Yield 55%. m.p. 222 °C; ^1^H NMR (4001 MHz, CDCl_3_): δ 8.03 (s, 2H); 7.86 (d, *J* = 8.0, 2H); 7.60 (s, 2H); 7.55 (d, *J* = 8.0, 2H); 4.50 (s, 4H); 4.26 (q, *J* = 7.1, 4H); 1.56 (s, 6H); 1.31 (t, *J* = 7.1, 6H). ^13^C NMR (100 MHz, CDCl_3_): δ 167.3; 166.2; 165.5; 155.2; 140.5; 134.6; 133.2; 130.0; 124.6; 121.5; 120.7; 62.1; 47.1; 42.2; 26.9; 14.1. MS (ESI^+^) *m*/z for C_31_H_28_N_2_O_8_S_2_: 643 [M + Na]^+^.

##### Diethyl 2,2′-((5Z,5′Z)-([2,2′-bithiophene]-5,5′-diylbis(methanylylidene))bis(2,4-dioxothiazolidin-3-yl-5-ylidene))diacetate (**12**)

The title compound **12** was obtained as a red solid, according to the general procedure using **26** and dialdehyde **34**. Yield 24%. m.p. 271 °C (dec.); ^1^H NMR (400 MHz, CDCl_3_/TFA-*d*_1_): δ 8.12 (s, 2H), 7.43-7.40 (m, 4 H), 4.53 (s, 4H), 4.28 (q, *J*_1_ = 8 Hz, *J*_2_ = 16 Hz, 4H), 1.30 (t, *J*_1_ = 4 Hz, 4H).^13^C NMR (100 MHz, CDCl_3_/TFA-*d*_1_): δ 160.83, 160.40, 126.67, 118.41, 112.76, 109.93, 63.15, 42.49, 13.83. MS (ESI^+^) *m*/z for C_24_H_20_N_2_O_8_S_4_: 615 [M + Na]^+^.

##### (5Z,5′Z)-5,5′-(1,4-Phenylenebis(methanylylidene))bis(3-(2-(dimethylamino)ethyl) thiazolidine-2,4-dione) (**19**)

The title compound **19** was obtained as a pale yellow solid, according to the general procedure using **28** and dialdehyde **29**. Yield 27%. m.p. 218 °C (dec.); ^1^H NMR (400 MHz, CDCl_3_): δ 7.87 (s, 2H); 7.59 (s, 4H); 3.88 (t, *J* = 6.5, 4H); 2.58 (t, *J* = 6.5, 4H); 2.28 (s, 12H). ^13^C NMR (100 MHz, CDCl_3_): δ 167.3; 166.1; 134.9; 131.9; 130.7; 123.6; 56.2; 45.5; 39.9. MS (ESI^+^) *m*/z for C_22_H_26_N_4_O_4_S_2_: 497 [M + Na]^+^.

##### (5Z,5′Z)-5,5′-([1,1′-Biphenyl]-4,4′-diylbis(methanylylidene))bis(3-(2-(dimethylamino) ethyl)thiazolidine-2,4-dione) (**20**)

The title compound **20** was obtained as a yellow solid, according to the general procedure using **28** and dialdehyde **30**. Yield 21%. m.p. 231 °C (dec.); ^1^H NMR (400 MHz, CDCl_3_): δ 7.92 (s, 2H); 7.73 (d, *J* = 8.3, 4H); 7.60 (d, *J* = 8.4, 4H); 3.88 (t, *J* = 6.5, 4H); 2.59 (t, *J* = 6.5, 4H); 2.29 (s, 12H). ^13^C NMR (100 MHz, CDCl_3_): δ 167.7; 166.3; 141.4; 133.0; 132.8; 130.8; 127.7; 122.0; 56.3; 45.5; 39.9. MS (ESI^+^) *m*/z for C_28_H_30_N_4_O_4_S_2_: 573 [M + Na]^+^.

##### (5Z,5′Z)-5,5′-((Methylenebis(4,1-phenylene))bis(methanylylidene))bis(3-(2-(dimethylamino)ethyl)thiazolidine-2,4-dione) (**21**)

The title compound **21** was obtained as a pale yellow solid, according to the general procedure using **28** and dialdehyde **31**. Yield 20%. m.p. 168 °C; ^1^H NMR (400 MHz, CDCl_3_): δ 7.86 (s, 2H); 7.45 (d, *J* = 8.0, 4H); 7.28 (d, *J* = 8.0, 4H); 4.06 (s, 2H); 3.87 (t, *J* = 6.4, 4H); 2.59 (t, *J* = 6.4, 4H); 2.29 (s, 12H). ^13^C NMR (100 MHz, CDCl_3_): δ 167.8; 166.4; 142.9; 133.3; 131.6; 130.6; 129.8; 121.2; 56.2; 45.4; 41.7; 39.7. MS (ESI^+^) *m*/z for C_29_H_32_N_4_O_4_S_2_: 587 [M + Na]^+^.

##### (5Z,5′Z)-5,5′-((9H-Carbazole-3,6-diyl)bis(methanylylidene))bis(3-(2-(dimethylamino) ethyl)thiazolidine-2,4-dione) (**22**)

The title compound **22** was obtained as a yellow solid, according to the general procedure using **28** and dialdehyde **32**. Yield 18%. m.p. 220 °C (dec.); ^1^H NMR (400 MHz, CDCl_3_): δ 10.80 (s, 1H); 7.77 (s, 2H); 7.68 (s, 2H); 7.41 (m, 4H); 3.94 (s, 4H); 2.78 (s, 4H); 2.45 (s, 12H). ^13^C NMR (100 MHz, CDCl_3_): δ 168.0; 166.3; 141.8; 134.4; 129.7; 124.7; 123.4; 122.1; 116.9; 111.8; 56.4; 45.3; 39.2. MS (ESI^+^) *m*/z for C_28_H_29_N_5_O_4_S_2_: 586 [M + Na]^+^.

##### (5Z,5′Z)-5,5′-((9,9-Dimethyl-9H-fluorene-2,7-diyl)bis(methanylylidene))bis(3-(2-(dimethylamino) ethyl)thiazolidine-2,4-dione) (**23**)

The title compound **23** was obtained as a dark yellow solid, according to the general procedure using **28** and dialdehyde **33**. Yield 21%. m.p. 199 °C (dec.); ^1^H NMR (400 MHz, CDCl_3_): δ 7.96 (s, 2H); 7.81 (d, *J* = 8.0, 2H); 7.56 (s, 2H); 7.51 (d, *J* = 8.0, 2H); 3.87 (t, *J* = 6.4, 4H); 2.59 (t, *J* = 6.4, 4H); 2.28 (s, 12H); 1.52 (s, 6H). ^13^C NMR (100 MHz, CDCl_3_): δ 167.81, 166.34, 155.14, 140.32, 133.78, 133.35, 129.94, 124.46, 121.36, 56.19, 47.08, 45.39, 29.67, 26.90. MS (ESI^+^) *m*/z for C_31_H_34_N_4_O_4_S_2_: 613 [M + Na]^+^.

##### (5Z,5′Z)-5,5′-([2,2′-Bithiophene]-5,5′-diylbis(methanylylidene))bis(3-(2-(dimethylamino) ethyl)thiazolidine-2,4-dione) (**24**)

The title compound **24** was obtained as a red solid, according to the general procedure using **28** and dialdehyde **34**. Yield 25%. m.p. 247 °C (dec.); ^1^H NMR (400 MHz, DMSO-*d*_6_): δ 8.20 (s, 2H); 7.73 (d, *J* = 4.0, 2H); 7.68 (d, *J* = 4.0, 2H); 4.01 (t, *J* = 5.6, 4H); 3.40 (t, *J* = 5.6, 4H); 2.87 (s, 12H). ^13^C NMR (100 MHz, DMSO-*d*_6_/TFA-*d*_1_): δ 167.1; 165.8; 142.1; 137.7; 136.9; 127.6; 126.1; 119.9; 54.3; 42.7; 37.3. MS (ESI^+^) *m*/z for C_24_H_26_N_4_O_4_S_4_: 585 [M + Na]^+^.

#### General Procedure for the Synthesis of Compounds **13–18**

The corresponding ester derivatives (0.1 mmol) were refluxed overnight in acetic acid (4 mL) and concentrated HCl (1 mL). The reaction mixture was filtered and the solid was washed three times with water, methanol, and dichloromethane (DCM) to give the final compounds.

##### 2,2′-((5Z,5′Z)-(1,4-Phenylenebis(methanylylidene))bis(2,4-dioxothiazolidin-3-yl-5-ylidene))diacetic acid (**13**)

The title compound **13** was obtained as a pale yellow solid, according to the general procedure using **7**. Yield 86%. m.p. 251 °C (dec.); ^1^H NMR (400 MHz, DMSO-*d*_6_): δ 13.48 (br s, 2H); 8.04 (s, 2H); 7.83 (s, 4H); 4.40 (s, 4H). ^13^C NMR (100 MHz, DMSO-*d*_6_): δ 168.3; 167.1; 165.4; 135.0; 132.9; 131.34; 123.0; 42.9. MS (ESI^–^) *m*/z for C_18_H_12_N_2_O_8_S_2_: 447 [M – H]^−^.

##### 2,2′-((5Z,5′Z)-([1,1′-Biphenyl]-4,4′-diylbis(methanylylidene))bis(2,4-dioxothiazolidin-3-yl-5-ylidene))diacetic acid (**14**)

The title compound **14** was obtained as a yellow solid, according to the general procedure using **8**. Yield 50%. m.p. 302 °C (dec.); ^1^H NMR (400 MHz, DMSO-*d*_6_): δ 13.42 (br s, 2H); 8.06 (s, 2H); 7.98 (d, *J* = 8.1, 4H); 7.79 (d, *J* = 8.1, 4H); 4.40 (s, 4H). ^13^C NMR (100 MHz, DMSO-*d*_6_): δ 168.4; 167.2; 165.4; 141.1; 133.7; 133.0; 131.5; 128.1; 121.4; 42.8. MS (ESI^–^) *m*/z for C_24_H_16_N_2_O_8_S_2_: 523 [M – H]^−^.

##### 2,2′-((5Z,5′Z)-((Methylenebis(4,1-phenylene))bis(methanylylidene))bis(2,4-dioxothiazolidin-3-yl-5-ylidene))diacetic acid (**15**)

The title compound **15** was obtained as a white solid, according to the general procedure using **9**. Yield 33%. m.p. 277 °C (dec.); ^1^H NMR (400 MHz, DMSO-*d*_6_): δ 7.96 (s, 2H); 7.60 (d, *J* = 8.2, 4H); 7.45 (d, *J* = 8.2, 4H); 4.37 (s, 4H); 4.09 (s, 2H). ^13^C NMR (100 MHz, DMSO-*d*_6_): δ 168.4; 167.3; 165.5; 144.3; 134.1; 131.3; 131.0; 130.3; 120.5; 42.7; 41.1. MS (ESI^–^) *m*/z for C_25_H_18_N_2_O_8_S_2_: 537 [M – H]^−^.

##### 2,2′-((5Z,5′Z)-((9H^–^Carbazole-3,6-diyl)bis(methanylylidene))bis(2,4-dioxothiazolidin-3-yl-5-ylidene))diacetic acid (**16**)

The title compound **16** was obtained as a yellow solid, according to the general procedure using **10**. Yield 50%. m.p. 285 °C (dec.); ^1^H NMR (400 MHz, DMSO-*d*_6_): δ 13.43 (br s, 2H); 12.17 (s, 1H); 8.55 (s, 2H); 8.16 (s, 2H); 7.75–7.79 (m, 4H); 4.40 (s, 4H). ^13^C NMR (100 MHz, DMSO-*d*_6_): δ 168.5; 167.6; 165.7; 142.1; 135.6; 129.0; 124.9; 124.7; 123.4; 117.3; 113.1; 42.7. MS (ESI^-^) *m*/z for C_24_H_15_N_3_O_8_S_2_: 536 [M – H]^−^.

##### 2,2′-((5Z,5′Z)-((9,9-Dimethyl-9H-fluorene-2,7-diyl)bis(methanylylidene))bis(2,4-dioxothiazolidin-3-yl-5-ylidene))diacetic acid (**17**)

The title compound **17** was obtained as a yellow solid, according to the general procedure using **11**. Yield 58%. m.p. 306 °C (dec.); ^1^H NMR (400 MHz, DMSO-*d*_6_): δ 8.11 (d, *J* = 8.0, 2H); 8.08 (s, 2H); 7.88 (s, 2H); 7.69 (d, *J* = 8.0, 2H); 4.41 (s, 4H); 1.53 (s, 6H). ^13^C NMR (100 MHz, DMSO-*d*_6_): δ 168.4; 167.3; 165.5; 155.4; 140.4; 134.4; 133.4; 130.1; 125.4; 122.5; 120.9; 47.3; 42.8; 26.8. MS (ESI^-^) *m*/z for C_27_H_20_N_2_O_8_S_2_: 563 [M – H]^−^.

##### 2,2′-((5Z,5′Z)-([2,2′-Bithiophene]-5,5′-diylbis(methanylylidene))bis(2,4-dioxothiazolidin-3-yl-5-ylidene))diacetic acid (**18**)

The title compound **18** was obtained as a red solid, according to the general procedure using **12**. Yield 30%. m.p. 339 °C (dec.); ^1^H NMR (400 MHz, DMSO-*d*_6_/TFA-*d*_1_): δ 8.16 (s, 2H); 7.66 (d, *J* = 4.0, 2H); 7.61 (d, *J* = 4.0, 2H); 4.34 (s, 4H). MS (ESI^+^) *m*/z for C_20_H_12_N_2_O_8_S_4_: 559 [M + Na]^+^. HR-MS calcd for C_24_H_19_N_2_O_8_S_4_ 534.9404, found 534.9402 [M – H]^−^.

#### Ethyl 2-(2,4-dioxothiazolidin-3-yl)acetate (**26**)

A mixture of **25** (2 mmol), **35** (2 mmol), and anhydrous K_2_CO_3_ (3 mmol) in acetone (12 mL) was reacted under microwave irradiation at 100 °C for 45 min. After the reaction, the solid was removed by filtration, and the solvent was evaporated under reduced pressure. The crude product was purified by column chromatography eluting with DCM/ethyl acetate (9.6:0.4). Yield 87%. ^1^H NMR (401 MHz, CDCl_3_): δ 4.24 (s, 2H); 4.13 (q, *J* = 7.2, 2H); 3.97 (s, 2H); 1.20 (t, *J* = 7.2, 3H).

#### 3-(2-(Dimethylamino)ethyl)thiazolidine-2,4-dione (**28**)

A mixture of **25** (2 mmol), **36** (2 mmol), and Cs_2_CO_3_ (2 mmol) in acetone (12 mL) was reacted under microwave irradiation at 100 °C for 45 min. After the reaction, the solid was removed by filtration, and the solvent was evaporated under reduced pressure. The crude product was purified by column chromatography eluting with DCM/ethanol/aqueous NH_4_^+^OH^-^ (9.5:0.5:0.05). Yield 53%. ^1^H NMR (401 MHz, CDCl_3_): δ 4.87 (s, 2H); 3.89 (s, 2H); 3.65 (t, *J* = 6.5, 2H); 2.42 (t, *J* = 6.5, 2H); 2.17 (s, 6H).

#### 4,4′-Methylenedibenzaldehyde (**31**)

Compound **37** (12 mmol) and paraformaldehyde (42 mmol) were dissolved in 8 mL of glacial acetic acid containing 33 wt % HBr. After being refluxed for 12 h, the reaction flask was immersed in an ice bath. The white greasy solid was separated by decantation and washed three times with water. Recrystallization from toluene afforded bis(4-(bromomethyl)phenyl)methane **38** (yield 20%). ^1^H NMR (401 MHz, CDCl_3_): δ 7.32 (d, *J* = 8.0, 4H); 7.15 (d, *J* = 8.0, 4H); 4.48 (s, 4H); 3.96 (s, 2H). Hexamethylenetetramine (6 mmol) and bis(4-(bromomethyl)phenyl)methane (2 mmol) were separately dissolved into 4 mL of chloroform. The two solutions were mixed and refluxed for 6 h. The white precipitate was filtered and dried in vacuum overnight. Then, it was dissolved in 7 mL of 50% acetic acid and refluxed for 6 h; 1 mL of concentrated HCl was added and refluxed for another 2 h. The reaction solution was extracted with ethyl ether and then washed with 5N NaOH. The concentration of the ether portion and recrystallization from ethanol give **27** as a white solid. Yield 42%. ^1^H NMR (401 MHz, CDCl_3_): δ 9.99 (s, 2H); 7.83 (d, *J* = 8.0, 4H); 7.35 (d, *J* = 8.0, 4H); 4.14 (s, 2H).

#### General Procedure for the Synthesis of Dialdehydes **32–34**

The corresponding starting dibromo derivatives (3 mmol) were dissolved in anhydrous THF (20 mL) and the solution was stirred in a dry ice/acetone bath; 8.5 mL of BuLi (2.5 M in hexane) was slowly added. The cooling bath was removed for 1 h and then placed again. After 10 min, anhydrous DMF (2.5 mL) was added dropwise. The cooling bath was removed, and the reaction was stirred for 90 min. After this period, 1M HCl (20 mL) was added, and the reaction was extracted twice with ethyl acetate. The combined extract was washed with brine, dried over Na_2_SO_4_, and concentrated. The final compounds were purified through column chromatography.

##### 9H-Carbazole-3,6-dicarbaldehyde (**32**)

The title compound **32** was obtained as a yellow solid, according to the general procedure using **39**. Column chromatography eluted with DCM/ethyl acetate/toluene 6:3:1. Yield 58%. ^1^H NMR (401 MHz, DMSO-*d*_6_): δ 12.35 (br s, 1H); 10.09 (s, 2H); 8.88 (d, *J* = 1.2, 2H); 8.02 (dd, *J* = 8.5, 1.2, 2H); 7.72 (d, *J* = 8.5, 2H). ^13^C NMR (101 MHz, DMSO-*d*_6_): δ 192.4; 144.7; 129.6; 127.6; 125.2; 123.1; 112.6.

##### 9,9-Dimethyl-9H-fluorene-2,7-dicarbaldehyde (**33**)

The title compound **33** was obtained as a yellow solid, according to the general procedure using **40**. Column chromatography eluted with DCM. Yield 45%. ^1^H NMR (401 MHz, CDCl_3_): δ 10.06 (s, 2H); 7.99 (s, 2H); 7.96–7.82 (m, 4H); 1.53 (s, 6H). ^13^C NMR (101 MHz, CDCl_3_): δ 191.9; 155.5; 143.7; 136.6; 130.3; 123.3; 121.6; 47.1; 26.7.

##### [2,2′-Bithiophene]-5,5′-dicarbaldehyde (**34**)

The title compound **34** was obtained as a dark yellow solid, according to the general procedure using **41**. Column chromatography eluted with DCM/petroleum ether/toluene/ethyl acetate 5:3:1.5:0.5. Yield 57%. ^1^H NMR (401 MHz, CDCl_3_): δ 9.91 (s, 2H); 7.72 (d, *J* = 4.0, 2H); 7.42 (d, *J* = 4.0, 2H). ^13^C NMR (101 MHz, CDCl_3_): δ 182.5; 144.8; 143.8; 136.9; 126.4.

### Aβ_42_ and Tau Antiaggregating Activity

#### Cloning and Overexpression of the Aβ_42_ Peptide

*Escherichia coli* competent cells BL21 (DE3) were transformed with the pET28a vector (Novagen, Inc., Madison, WI) carrying the DNA sequence of Aβ_42_. Because of the addition of the initiation codon ATG in front of both genes, the overexpressed peptide contains an additional methionine residue at its N terminus. For overnight culture preparation, an amount of 10 mL of M9 minimal medium containing 50 μg·mL^–1^ kanamycin was inoculated with a colony of BL21 (DE3) bearing the plasmid to be expressed at 37 °C. For expression of the Aβ_42_ peptide, the required volume of overnight culture to obtain 1:500 dilution was added into fresh M9 minimal medium containing 50 μg·mL^–1^ kanamycin and 250 μM ThS. The bacterial culture was grown at 37 °C and 250 rpm. When the cell density reached ABS_600 nm_ = 0.6, an amount of 980 μL of culture was transferred into Eppendorf tubes of 1.5 mL with 10 μL of each compound to be tested in DMSO and 10 μL of isopropyl 1-thio-β-d-galactopyranoside (IPTG) at 100 mM. The final concentration of the drug was fixed at 10 μM. The samples were grown overnight at 37 °C and 1400 rpm using a thermomixer (Eppendorf, Hamburg, Germany). As the control of the amyloid presence (maximal amyloid presence), the same amount of DMSO without the drug was added to the sample. In parallel, noninduced samples (in the absence of IPTG) were also prepared and used as controls of the non-amyloid presence. In addition, the absorbance at 600 nm of the samples was checked to assess the potential intrinsic toxicity of the compounds and to confirm the correct bacterial growth.

#### Cloning and Overexpression of Tau Protein

*E. coli* BL21 (DE3) competent cells were transformed with pTARA containing the RNA-polymerase gen of the T7 phage (T7RP) under the control of the promoter PBAD. *E. coli* BL21 (DE3) with pTARA competent cells were transformed with the pRKT42 vector encoding four repeats of Tau protein in two inserts. For overnight culture preparation, 10 mL of M9 medium containing 0.5% of glucose, 100 μg·mL^–1^ ampicillin, and 12.5 μg·mL^–1^ chloramphenicol were inoculated with a colony of BL21 (DE3) bearing the plasmids to be expressed at 37 °C. For expression of Tau protein, the required volume of overnight culture to obtain 1:500 dilution was added to fresh M9 minimal medium containing 0.5% of glucose, 50 μg·mL^–1^ ampicillin, 12.5 μg·mL^–1^ chloramphenicol, and 25 μM ThS. The bacterial culture was grown at 37 °C and 250 rpm. When the cell density reached ABS_600 nm_= 0.6, an amount of 980 μL of culture was transferred into Eppendorf tubes of 1.5 mL with 10 μL of each compound to be tested in DMSO and 10 μL of arabinose at 25%. The final concentration of the drug was fixed at 10 μM. The samples were grown overnight at 37 °C and 1400 rpm using a thermomixer (Eppendorf, Hamburg, Germany). As the control of maximal amyloid presence, the same amount of DMSO without the drug was added to the sample. In parallel, noninduced samples (in the absence of arabinose) were also prepared and used as controls for the amyloid absence. In addition, the absorbance at 600 nm of the samples was checked to assess the potential intrinsic toxicity of the compounds and to confirm the correct bacterial growth.

#### ThS Steady-State Fluorescence

ThS (T1892) and other chemical reagents were purchased from Sigma (St. Louis, MO). ThS stock solution (25 mM) was prepared in double-distilled water purified through a Milli-Q system (Millipore). For the fluorescence assay, the ThS spectra were measured on an AMINCO-Bowman series 2 luminescence spectrophotometer (Aminco-Bowman AB2, SLM Aminco, Rochester, NY) from 460 to 600 nm at 25 °C using an excitation wavelength of 440 nm and slit widths of 4 nm. The emission at 485 nm (fluorescence peak of ThS in the presence of amyloids) was recorded. To normalize the ThS fluorescence as a function of the bacterial concentration, ABS_600nm_ was obtained using a Shimadzu UV-2401 PC UV–Vis spectrophotometer (Shimadzu, Japan). Note that the fluorescence normalization was carried out considering as 100% the ThS fluorescence of the bacterial cells expressing the peptide or protein in the absence of the drug and 0% the ThS fluorescence of the bacterial cells nonexpressing the peptide or protein.

#### Neurotoxicity Evaluation on Primary Cultures of Cerebellar Granule Neurons

Primary cultures of CGNs were prepared from 7-day-old pups of Wistar rats (*Rattus norvegicus*). All animal experiments were authorized by the University of Bologna Bioethical Committee (Protocol no. 1088; Code 2DBFE.N.BFY) and performed according to Italian and European Community laws on the use of animals for experimental purposes. For cerebellar granule cultures, cells were dissociated from cerebella and plated on 35 mm Ø dishes, previously coated with 10 μg/mL poly-l-lysine, at a density of 1.2 × 10^6^ cells/2 mL of medium BME supplemented with 100 mL/L heat-inactivated FBS (Life Technologies), 2 mmol/L glutamine, 100 μmol/L gentamicin sulfate, and 25 mmol/L KCl (all from Sigma-Aldrich); 16 h later, 10 μM cytosine arabinofuranoside (Sigma-Aldrich) was added to avoid glial proliferation. After 7 days in vitro, differentiated neurons were shifted to serum-free BME medium containing 25 mmol/L KCl without the serum and different treatments were performed. For neurotoxicity experiments, differentiated CGNs were exposed to 10 μM concentration of the studied compounds for 24 h in serum-free BME (25 mM KCl). The experiment was performed in triplicate. Since the native fluorescence of the library could interfere with the reading of the classical MTT cell viability assay, the Hoechst 33258 staining was performed to count healthy and apoptotic nuclei.

#### Nuclei Counting after Hoechst Staining

For nuclei counting after 24 h treatment, CGNs were fixed for 20 min with 4% PFA in phosphate buffer, washed in PBS, and incubated with 0.1 μg/mL of Hoechst 33258 (Sigma-Aldrich) for 5 min at room temperature. After Hoechst staining, four randomly selected fields were acquired from each condition with a fluorescence microscope (20× objective; Eclipse Hoechst staining TE 2000-S microscope, Nikon equipped with an AxioCam MRm (Zeiss, Oberkochen, Germany) digital camera. Neuronal survival was expressed as the percentage of healthy nuclei on the total nuclei number (mean ± SD) (Table 1).

#### PAMPA–BBB Assay

PAMPA (the parallel artificial membrane permeability assay) is a high-throughput screening tool applicable for the prediction of the passive transport of potential drugs across the BBB.^41^ In this study, it has been used as a non-cell-based *in vitro* assay carried out in a coated 96-well membrane filter. The filter membrane of the donor plate was coated with PBL (Polar Brain Lipid, Avanti) in dodecane (4 μL of 20 mg/mL PBL in dodecane) and the acceptor well was filled with 300 μL of phosphate buffer saline, (PBS pH 7.4; *V*_A_). The tested compounds were dissolved first in DMSO and then diluted with PBS pH 7.4 to reach the final concentrations of 50–500 μM in the donor well. The final concentration of DMSO did not exceed 0.5% (v/v) in the donor solution. Then, 300 μL of the donor solution (*V*_D_) was added to the donor wells and the donor filter plate was carefully put on the acceptor plate so that the coated membrane was “in touch” with both donor solution and acceptor buffer. In principle, the test compound diffuses from the donor well through the polar brain lipid membrane (area = 0.28 cm^2^) to the acceptor well. The concentrations of the tested compound in both donor and the acceptor wells were assessed after 3, 4, 5, and 6 h of incubation in quadruplicate using a UV plate reader, Synergy HT (Biotek) at the maximum absorption wavelength of each compound (n=3). In addition to that, solution of the theoretical compound concentration, simulating the equilibrium state established if the membrane were ideally permeable, was prepared and assessed as well. The concentrations of the compounds in the donor and acceptor wells and the equilibrium concentration were calculated from the standard curve and expressed as the permeability (Pe) according to the equation^41^
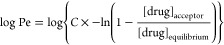
where
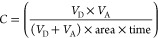


#### Evaluation of Optical Properties of **23** in the Presence of Aggregated Aβ42

As reported in a previously published protocol,^42^ 1,1,1,3,3,3,-hexafluoro-2-propanol (HFIP)-pretreated Aβ42 samples (Bachem AG, Switzerland) were solubilized with a CH_3_CN/0.3 mM Na_2_CO_3_/250 mM NaOH (48.4:48.4:3.2) mixture to obtain a 500 μM solution. Aggregation was achieved by diluting the peptide to a final concentration of 50 μM with 10 mM phosphate buffer (pH = 8.0) containing 10 mM NaCl and incubating the diluted solution at 30 °C for 24 h. This protocol was previously shown to give a reproducible aggregation kinetics.^42^

A 1.5 mM stock solution of **23** in methanol was prepared and diluted in 50 mM glycine–NaOH buffer (pH 8.5) to a final concentration of 0.375 μM. Excitation and emission fluorescence spectra in the absence and in the presence of preaggregated Aβ42 (0.75 μM) were recorded using the following instrument setup: λ_exc_ = 374 nm, emission range = 400–600 nm; λ_em_ = 473 nm; excitation range = 250–460 nm; bandwidth (Ex) = 5 nm; bandwidth (Em) = 5 nm; scanning speed = 500 nm.

#### Inhibition of Tau_(306-336)_ Peptide Aggregation

Briefly, 1 mg of Tau_(306-336)_ (Bachem AG, Germany) was dissolved in 1,1,1,3,3,3,-hexafluoro-2-propanol (HFIP), gently vortexed, sonicated, and kept overnight at room temperature. Subsequently, the sample was aliquoted, dried, and stored at −20 °C. Stock solutions of Tau_(306-336)_ peptide (500 μM) were prepared in ultrapure water and immediately used. Stock solution of Thioflavin T (ThT, 500 μM) was prepared in 56.3 mM phosphate buffer (PB, pH = 7.4), while stock solutions of inhibitors (20 mM) and of the reference compound doxycycline (10 mM) were prepared in DMSO/methanol 10/90. Tau_(306-336)_ aggregation was monitored at 30 °C in a black, clear bottom 96-well plate (Greiner) by the EnSpire multiplate reader (Perkin Elmer) using the ThT fluorimetric assay^14^ with some variations. The excitation and emission wavelengths were set at 446 and 490 nm, respectively. Assay samples were prepared by diluting Tau_(306-336)_ stock solution to 50 μM in the assay mixture which consisted of 20 μM ThT, 48.1 mM PB (final concentrations) in the final 100 μL volume (final DMSO and MeOH contents: 0.05 and 0.45%, respectively). Inhibition experiments were performed by incubating Tau_(306–336)_ peptide in the given conditions in the presence of tested inhibitors at 50 μM. Fluorescence data were recorded every 10 min overnight with 1 min shaking at 800 rpm prior to each reading. Each inhibitor was assayed in triplicate in at least two independent experiments. Estimation of the inhibitory potency (%) was carried out by comparing fluorescence values at the plateau (average fluorescence intensity value in the 12–16 h range). Inhibition % is expressed as the mean ± SEM. Quenching of ThT fluorescence was evaluated by preparing blank solutions containing the inhibitor/reference compound and preformed fibrils of Tau_(306–336)_ peptide.

#### Drosophila Melanogaster Model of AD

Flies w^1118^ (control flies indicated as CTR) and Elav > Gal4;UAS-ArcticAbeta42 (experimental population) were maintained at 25 °C and flipped into new vials every 2 days. The experimental flies take advantage of the UAS-Gal4 binary system, where these two are not found in the fly genome, and thus their introduction permits extremely specific control of the transgene expression.^63^ The transactivating protein GAL4 is placed under the control of a specific promoter, with its own spatial and temporal patterns, in this case, Elav, whereas the upstream activation sequence (UAS) localized upstream, the *locus* controlled by the UAS-Gal4; in our study, UAS expresses construct for the E22G variant of Aβ42 (Arctic Aβ42) (AlzArc2).^56,64^ Around 150 flies (half female/male) for each compound tested were collected and divided into groups of 25 flies. At the top of the media, 25 μL of doxycycline (50 μM), **22** (20 μM), or **23** (20 μM) were added fresh anytime the flies were flipped.

#### Climbing Assay and the Survival Rate

The behavioral assays follow the protocol illustrated in Albertini et al.^59^ Briefly, males and females 25 per vial were kept at 25 °C. The climbing test was performed on days 7, 14, and 21 after birth. Flies were placed inside a 50 mL transparent glass cylinder and, once acclimated, the cylinder was tapped down hard enough to knock all of the flies down to the bottom; after 10 s the number of flies able to reach three pre-established levels (below 5 cm–between 5 and 7.5 cm-above 10 cm) was counted. The protocol was repeated 10 times at 5-min intervals.

#### Immunofluorescence on Adult Brain

The dissection of each tissue analyzed was performed through the use of thin forceps in a dissection dish filled with 1% PBS solution, following a published protocol.^65^ After fixation in PFA 4% (paraformaldehyde) for 20 min, the samples were cleaned from fat bodies and tracheal tube residues, in 1% PBS solution. In the end, they were moved to a 1.5 mL Eppendorf tube for the subsequent steps. The permeabilization of the membranes used PBS Triton (PBST) 0.3% solution, which is a detergent used also in tissue culture analysis. The primary antibody anti-Aβ_42_ complexes (Alexa Fluor 594 #803018) were added to the blocking solution, at the concentration suggested 1:1000 and moved at 4 °C overnight. After washes in PBST for 20 min, each of the brains was stained with the secondary antibody (mouse FITC 1:250 Invitrogen #F2761). The samples were mounted in Fluoromount and the images were captured using a Leica confocal microscope, acquired with a 20× air objective, and a 60× mineral oil objective. The magnifications used the Nyquist theorem not to exceed the zoom and capture false signals. FluoView software was used for acquisition and Fiji (ImageJ) software for analysis.

## Supporting Information Available

Prediction of BBB penetration of compounds **7**, **10**, **16**, **17**, **22**, and **23**; chemical structures of reference compounds; inhibition of Tau_(306-336)_ self-aggregation by **22** and **23**; compounds’ purity and copies of representative chromatograms (**7**, **10**, **22** and **23**); and copies of ^1^H NMR and ^13^C NMR spectra for final compounds (**1**–**24**) (PDF)

## Abbreviations Used

Aβ: amyloid β
AD: Alzheimer’s disease
BBB: blood–brain barrier
CGNs: cerebellar granule neurons
DMF: dimethylformamide
*E. coli*: *Escherichia coli*
EDDA: ethylenediamine diacetate
EMA: European Medicines Agency
ESI: electrospray ionization
FDA: Food and Drug Administration
HMTA: hexamethylenetetramine
IPTG: isopropyl 1-thio-β-d-galactopyranoside
MTDL: multitarget-directed ligand
MWI: microwave irradiation
NMR: nuclear magnetic resonance
PB: phosphate buffer
Pe: effective permeability
PPIs: protein–protein interactions
PRMs: protein recognition motifs
ThS: thioflavin S
ThT: thioflavin T
TLC: thin-layer chromatography
TMS: tetramethylsilane
TZD: 2,4-thiazolidinedione
