# Supplementary material for: Discovery of Dual Aβ/Tau Inhibitors and Evaluation of Their Therapeutic Effect on a Drosophila Model of Alzheimer’s Disease
Source: ACS Chem Neurosci. 2022 Nov 29;13(23):3314–29. doi: 10.1021/acschemneuro.2c00357 (PMC9732823; doi:10.1021/acschemneuro.2c00357)

Supporting Information

Discovery of Dual Aβ/Tau Inhibitors and Evaluation of Their Therapeutic Effect on a *Drosophila* Model of Alzheimer's Disease

Annachiara Gandini,[a, b] *Ana Elisa Gonçalves,*[a, c] *Silvia Strocchi,*[a] *Claudia Albertini,*[a] *Jana Janočková,*[d] *Anna Tramarin,*[a] *Daniela Grifoni,*[a, e] *Eleonora Poeta,*[a] *Ondrej Soukup,*[d] *Diego Muñoz-Torrero,*[f] *Barbara Monti,*[c] *Raimon Sabaté,*[g] *Manuela Bartolini,*[a] *Giuseppe Legname,*[b] *Maria Laura Bolognesi**[a]

(a) Department of Pharmacy and Biotechnology, Alma Mater Studiorum - University of Bologna, Via Belmeloro 6, I-40126 Bologna, Italy; *Email: marialaura.bolognesi@unibo.it (b) Department of Neuroscience, Laboratory of Prion Biology, Scuola Internazionale Superiore di Studi Avanzati (SISSA), Via Bonomea 265, I-34136 Trieste, Italy; (c) Pharmaceutical Sciences Postgraduate Program, Center of Health Sciences, Universidade do Vale do Itajaí, Rua Uruguai 458, 88302-202, Itajaí, Santa Catarina, Brazil; (d) Biomedical Research Center, University Hospital Hradec Kralove, 500 00, Hradec Kralove, Czech Republic; (e) Department of Life, Health and Environmental Sciences, University of L'Aquila, Via Vetoio, Coppito II, 67100 L'Aquila, Italy; (f) Laboratory of Medicinal Chemistry (CSIC Associated Unit), Faculty of Pharmacy and Food Sciences, and Institute of Biomedicine (IBUB), University of Barcelona (UB), Av. Joan XXIII 27-31, E-08028 Barcelona, Spain; (g) Department of Pharmacy and Pharmaceutical Technology and Physical Chemistry, Faculty of Pharmacy and Food Sciences, University of Barcelona, Av Joan XXIII 27-31, E-08028 Barcelona, Spain.

Table of contents

1. Table S1. Prediction of BBB penetration of the studied compounds
2. Figure S1. Chemical structures of reference compounds

3. Table S2. Inhibition of $Tau_{(306\text{-}336)}$ peptide self-aggregation

4. Compounds' purity, copies of representative chromatograms

5. Copies of ^{1}H-NMR and ^{13}C-NMR spectra

Table S1. Prediction of BBB penetration of the studied compounds expressed as Pe ± SEM (n=3)

Compound	BBB Penetration Estimation	
	Pe ± SEM ($\times 10^{-6}$ cm s^{-1})	CNS (+/-)[a]
**7**	11.36 ± 0.49[b]	CNS +
**10**	5.79 ± 1.16[b]	CNS +
**16**	0.55 ± 0.12	CNS -
**17**	0.88 ± 0.20	CNS -
**22**	5.30 ± 1.17	CNS +
**23**	18.9 ± 5.39	CNS +
Furosemide	0.19 ± 0.07	CNS -
Chlorothiazide	1.14 ± 0.53	CNS -
Cefuroxime	0.62 ± 0.16	CNS -
Donepezil	21.49 ± 2.05	CNS +
Rivastigmine	20.00 ± 2.07	CNS +
Tacrine	5.96 ± 0.59	CNS +

[a] CNS + (high BBB permeation predicted): *Pe* ($\times 10^{-6}$ cm s^{-1}) > 4.0; CNS - (low BBB permeation predicted): *Pe* ($\times 10^{-6}$ cm s^{-1}) < 2.0; CNS +/- (uncertain BBB permeation): *Pe* ($\times 10^{-6}$ cm s^{-1}) from 4.0 to 2.0. [b] Study monitoring the compound stability in the PAMPA buffer solution showed a slow degradation over time (< 20% after 6h). However, the slow degradation was observed in both donor and acceptor wells, indicating that it has negligible relevance for the permeability results.

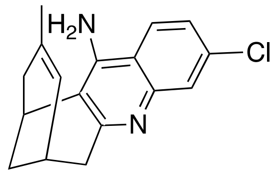

(±)-huprine Y

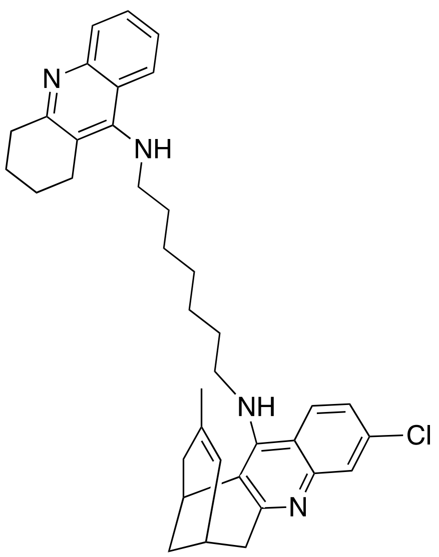

HUP7TH

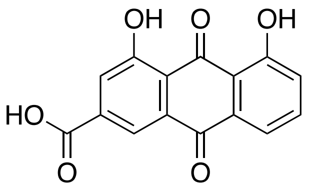

rhein

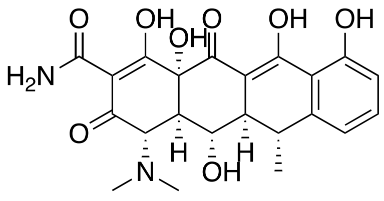

doxycycline

Figure S1. Chemical structures of reference compounds.

Table S2. Inhibition of $Tau_{(306-336)}$ peptide self-aggregation

Compound[1]	Inhibition of $Tau_{(306-336)}$ peptide self - aggregation (%) ± SEM [*]
**Doxycycline**	61.5 ± 0.8
**22**	NS
**23**	51.8 ± 11.7

[*] The results are the mean of at least two independent measurements each performed in duplicate.
[1] Compounds were measured at 50 μM.
NS = not soluble in the assay conditions

Compounds' purity.

Purity of final compounds **1-24** was determined using a Waters Spherisorb® ODS2 HPLC column (5μm, 250 × 4.6 mm) and a HPLC Jasco Corporation (Tokyo, Japan) instrument, model PU-1585 UV equipped with a 20 μL loop valve. HPLC parameters were the following: water with 0.05% trifluoroacetic acid (eluent A), and acetonitrile with 0.05% trifluoroacetic acid (eluent B); detection UV-Vis Abs at 254 nm. Two different elution conditions were used. Condition 1 (compounds **1-18**): flow rate 0.6 mL/min; elution type isocratic; 75% eluent A and 25% eluent B. Condition 2 (compounds **19-24)**: flow rate 0.4 mL/min; elution type isocratic 80% eluent A and 20% eluent B. All samples were dissolved in DMSO (10 μg/mL).

Table S3. Compounds' Purity by HPLC.

Compound	Purity (%)	Compound	Purity (%)	Compound	Purity (%)
**1**	98.27	**9**	97.75	**17**	99.66
**2**	99.53	**10**	99.62	**18**	96.32
**3**	96.82	**11**	98.92	**19**	96.58
**4**	96.41	**12**	95.88	**20**	98.14
**5**	97.03	**13**	100.00	**21**	98.47
**6**	98.36	**14**	96.90	**22**	100.00
**7**	98.00	**15**	96.37	**23**	99.55
**8**	99.74	**16**	98.98	**24**	97.84

Copies of representative chromatograms.

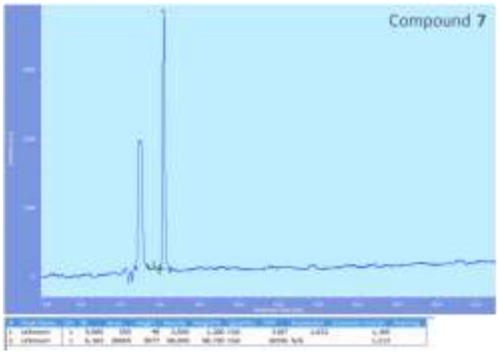

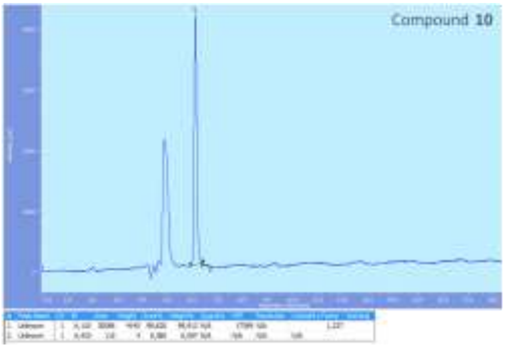

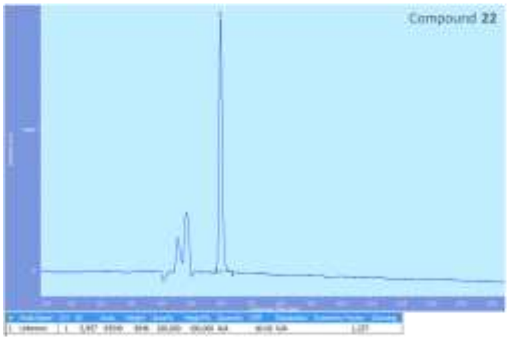

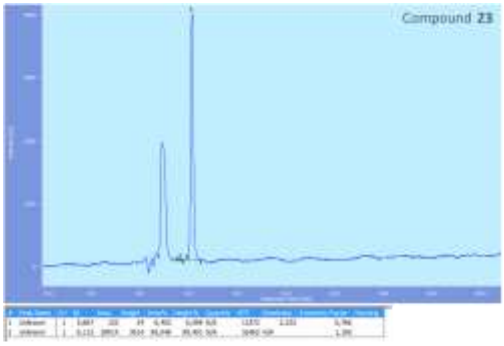

Copies of ^{1}H-NMR and ^{13}C-NMR spectra.

<u>Compound **1**</u>

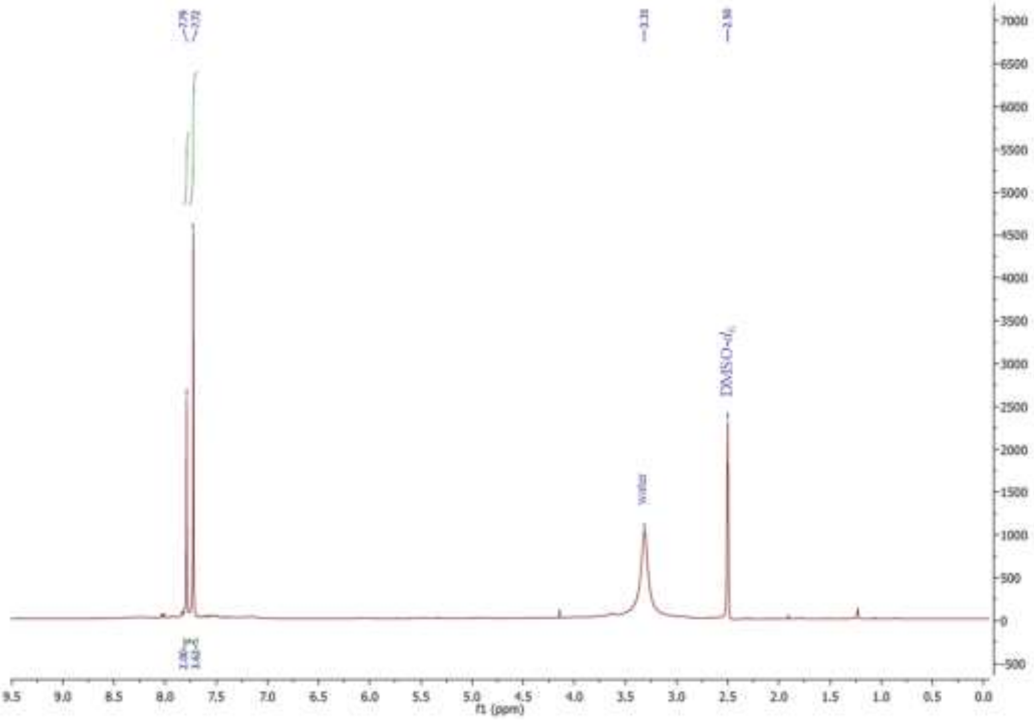

We were unable to acquire ^{13}C-NMR, due to the low solubility of the compound.

Compound **2**

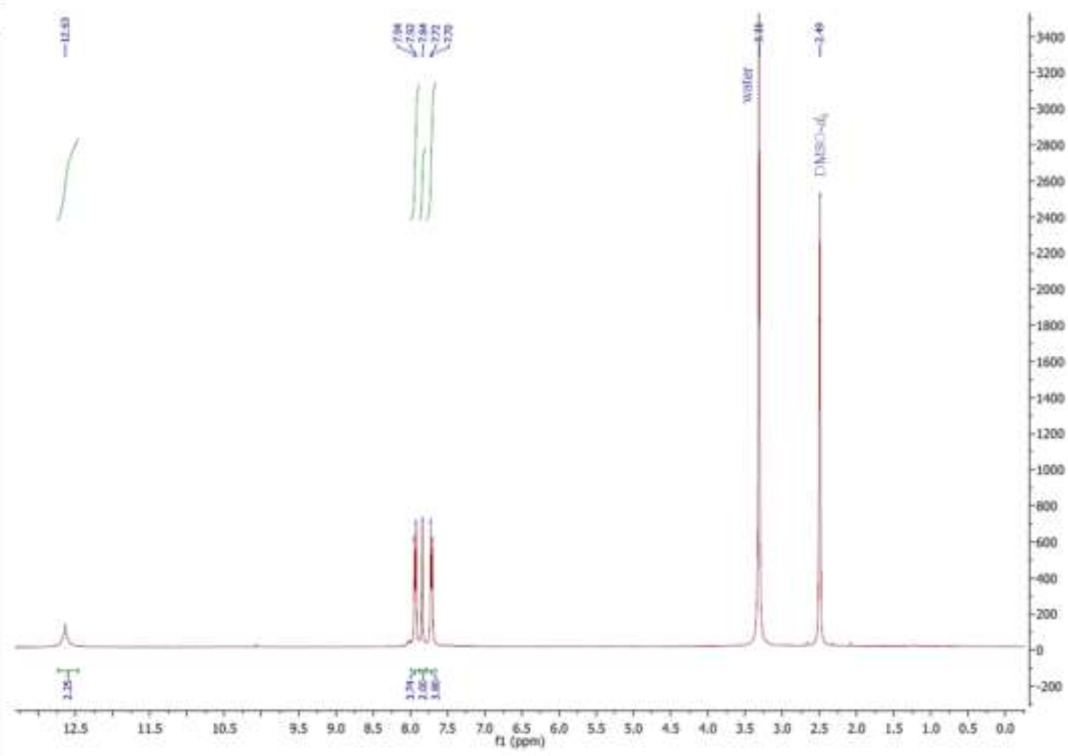

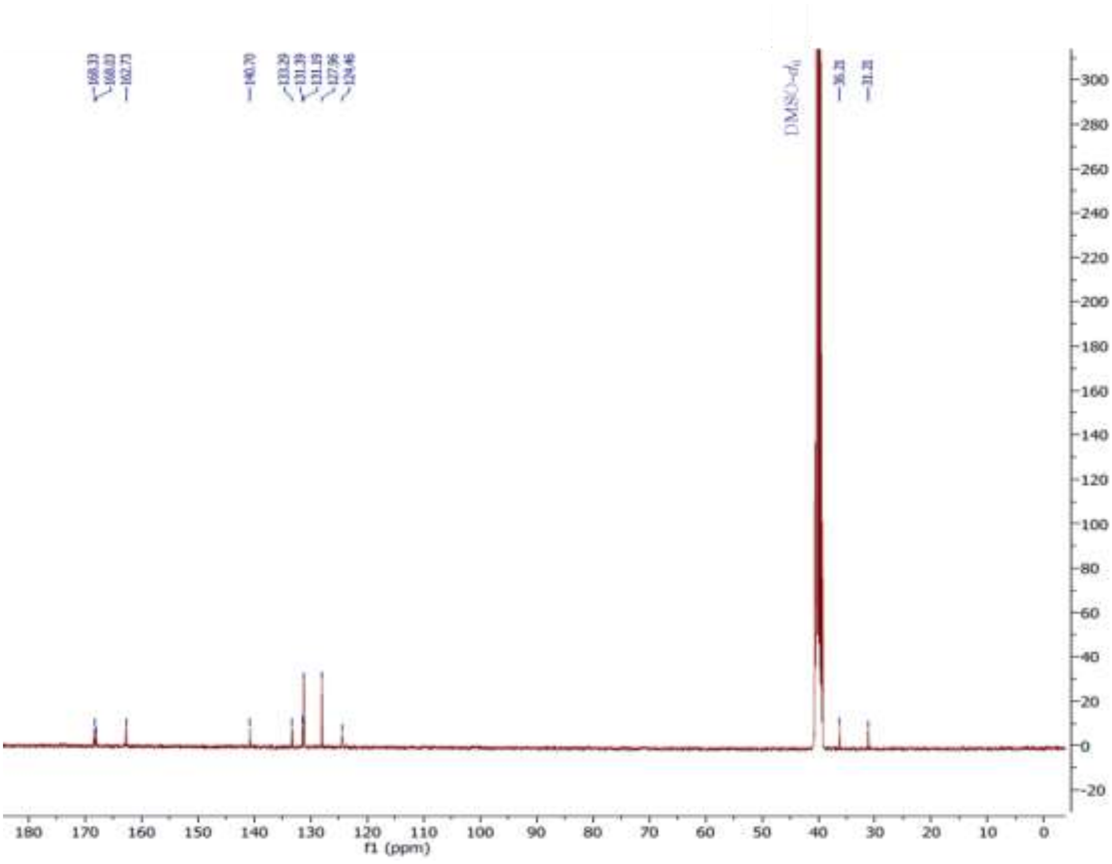

Compound **3**

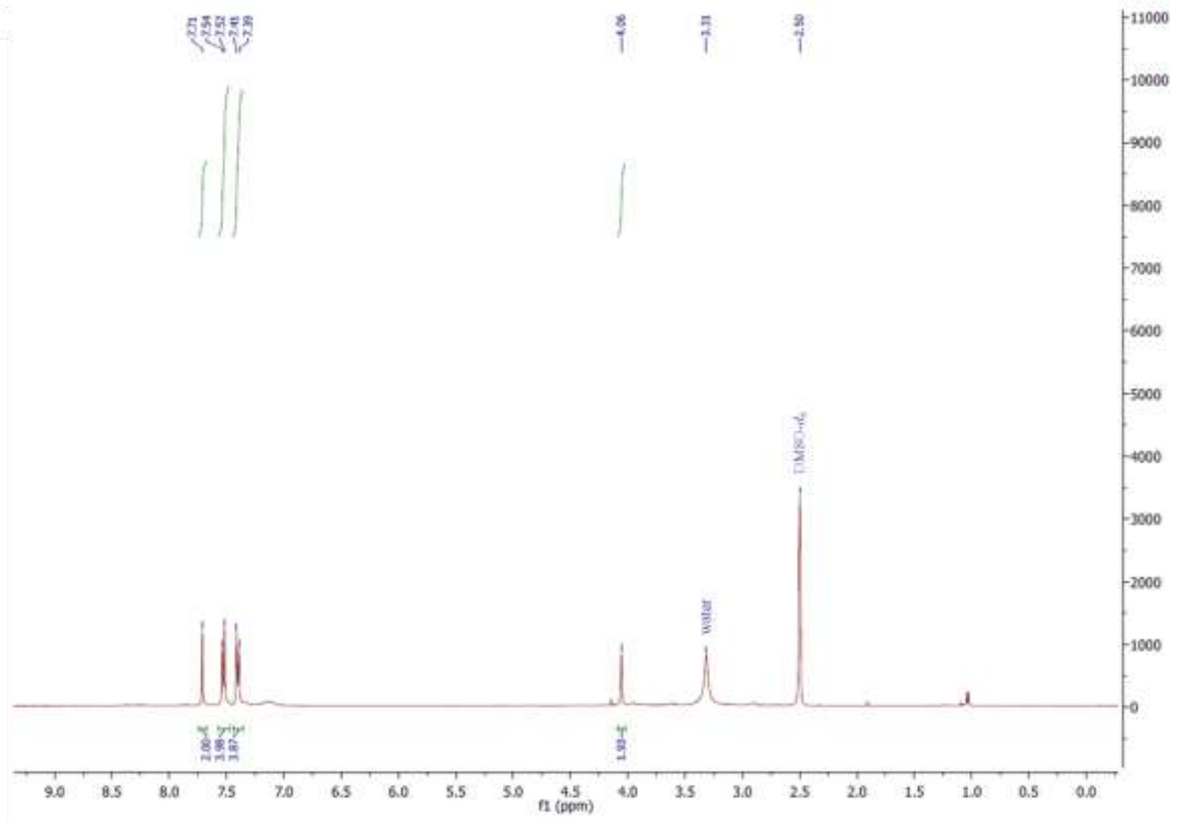
7.71
7.54
7.52
7.41
7.39
4.06
3.31
2.50
DMSO-d_6
water
2.00
3.98
3.87
1.93
f1 (ppm)

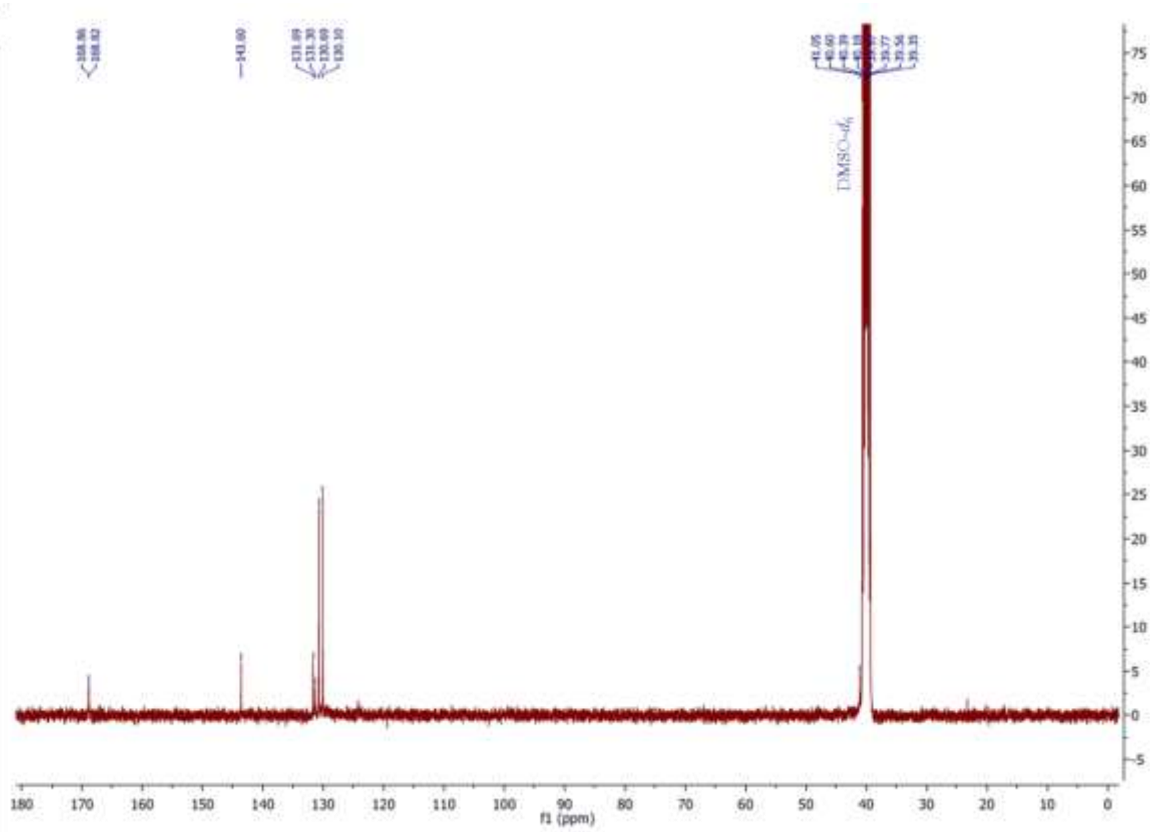
DMSO-d_6
f1 (ppm)

Compound **4**

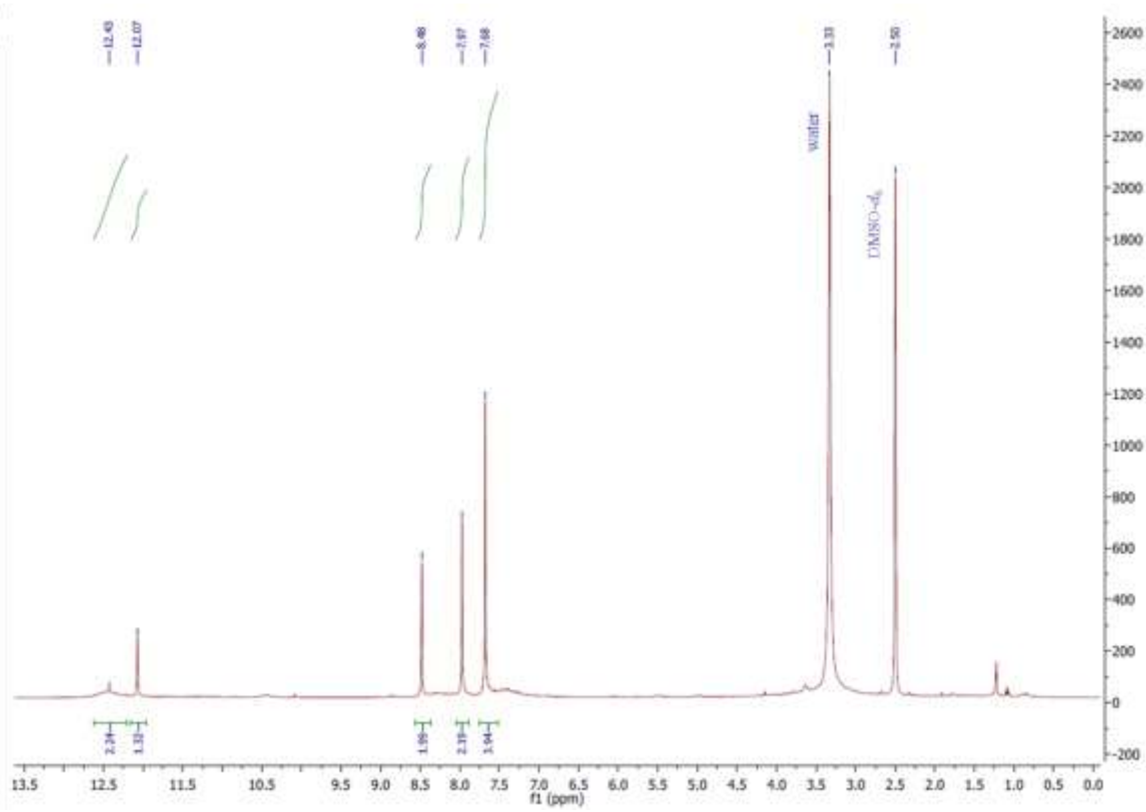

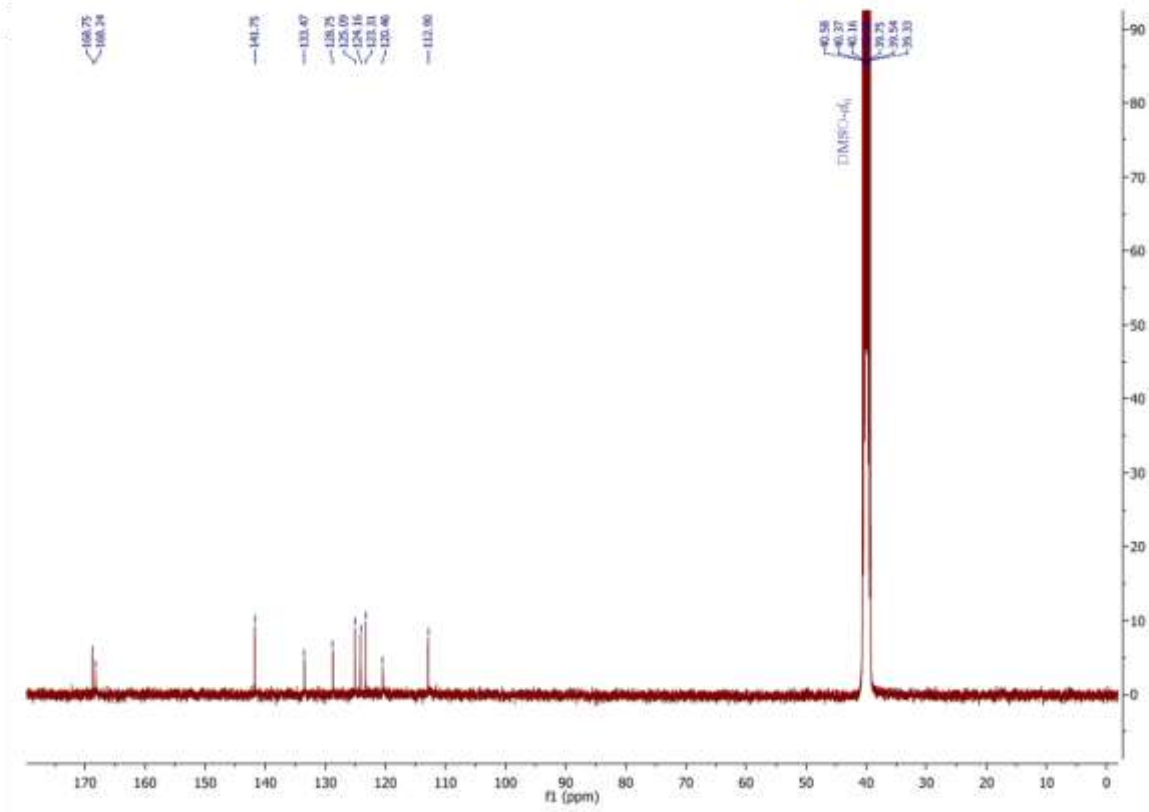

Compound **5**

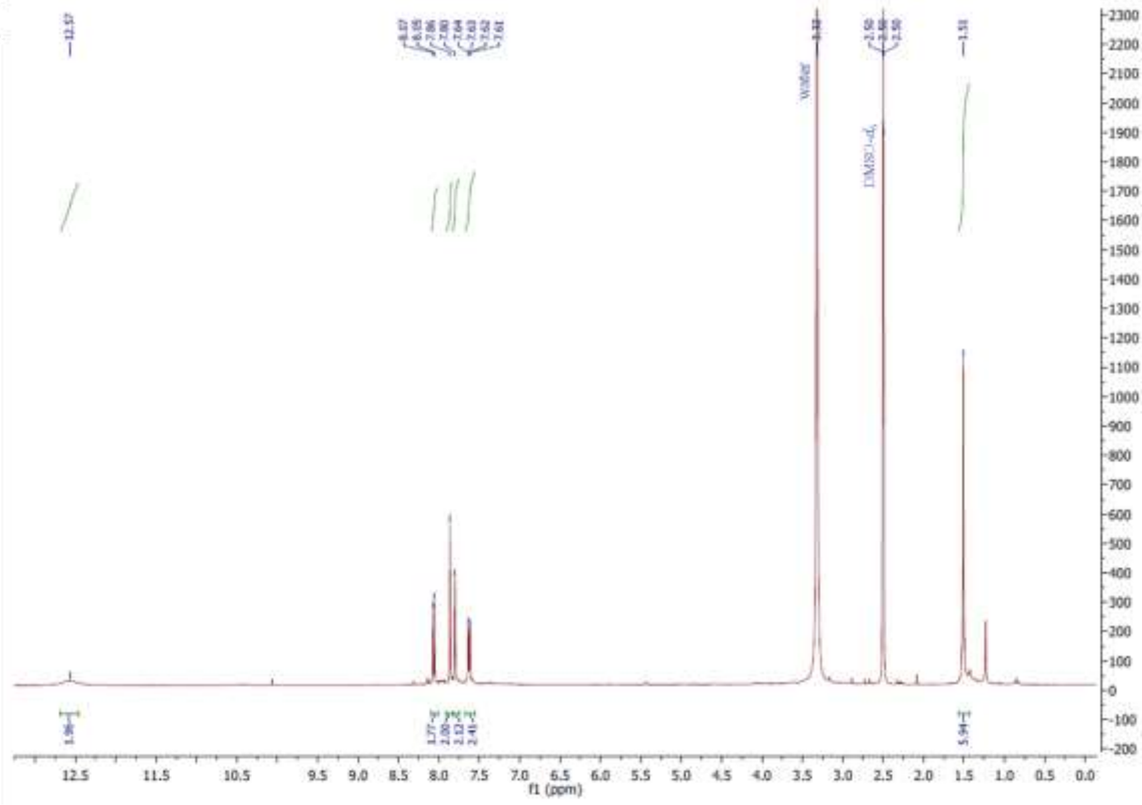

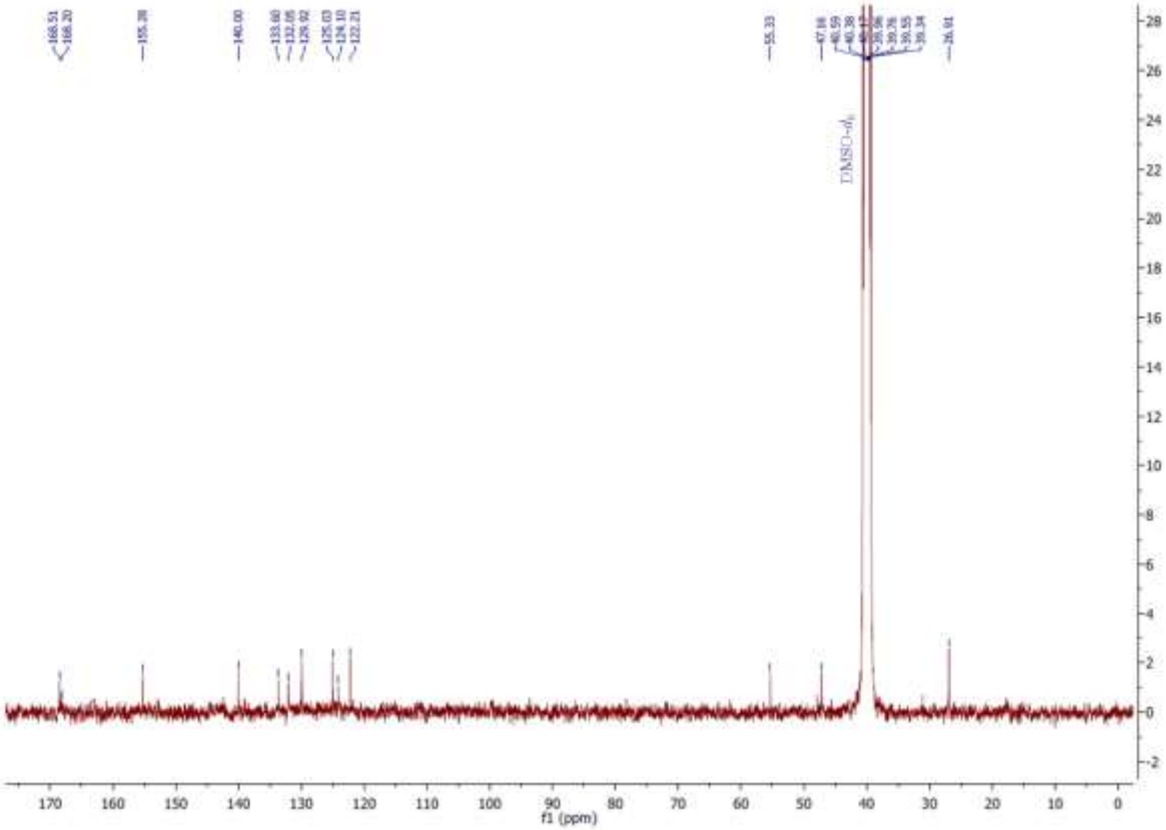

Compound **6**

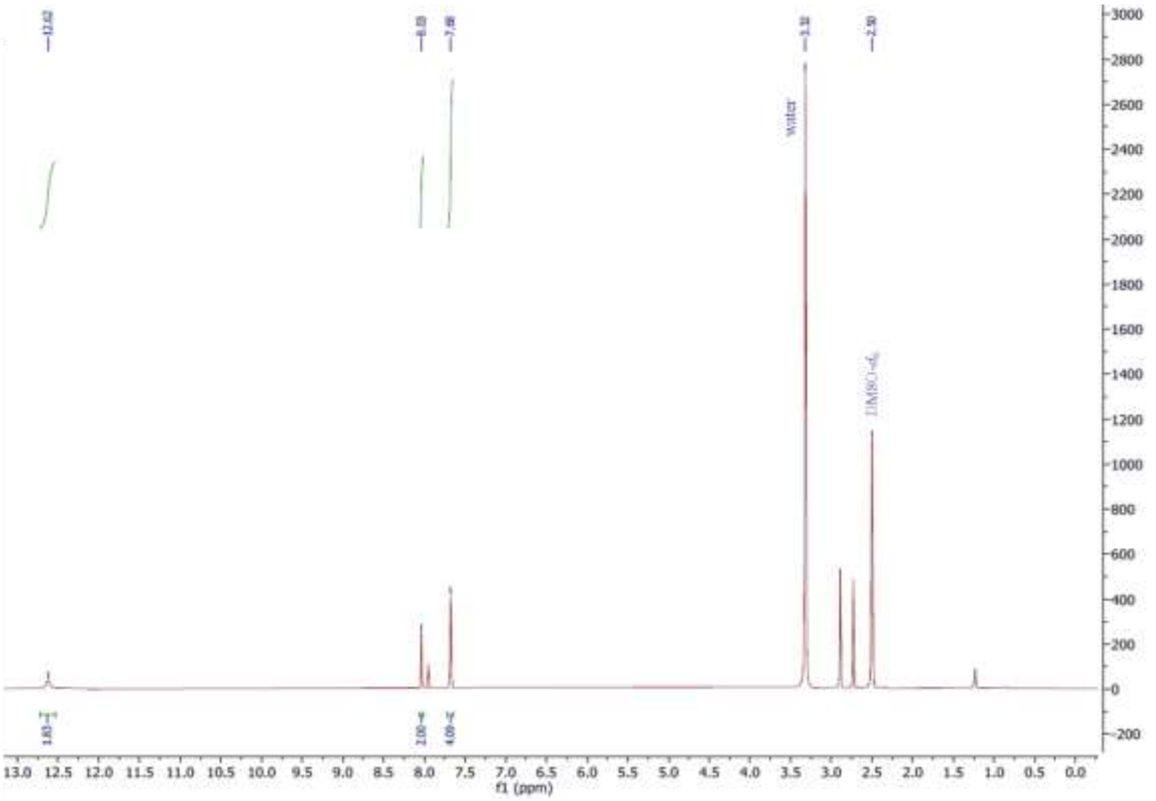

We were unable to acquire ^{13}C-NMR, due to the low solubility of the compound.

Compound **7**

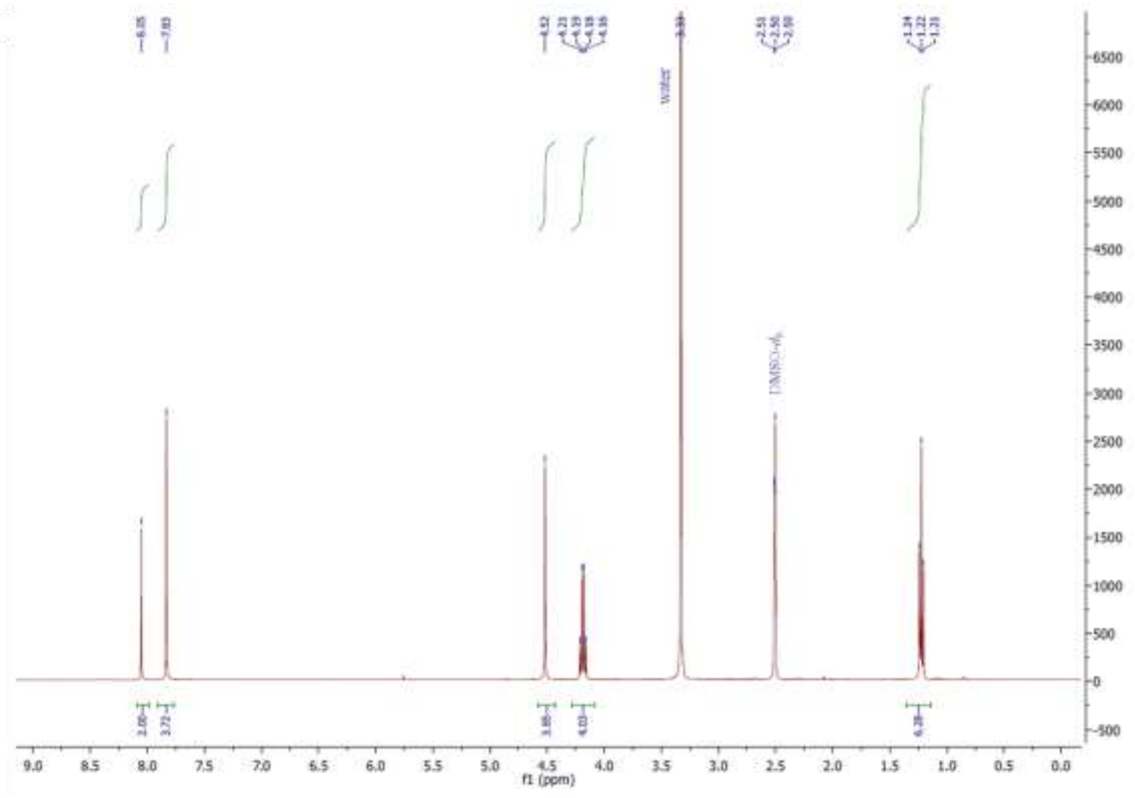

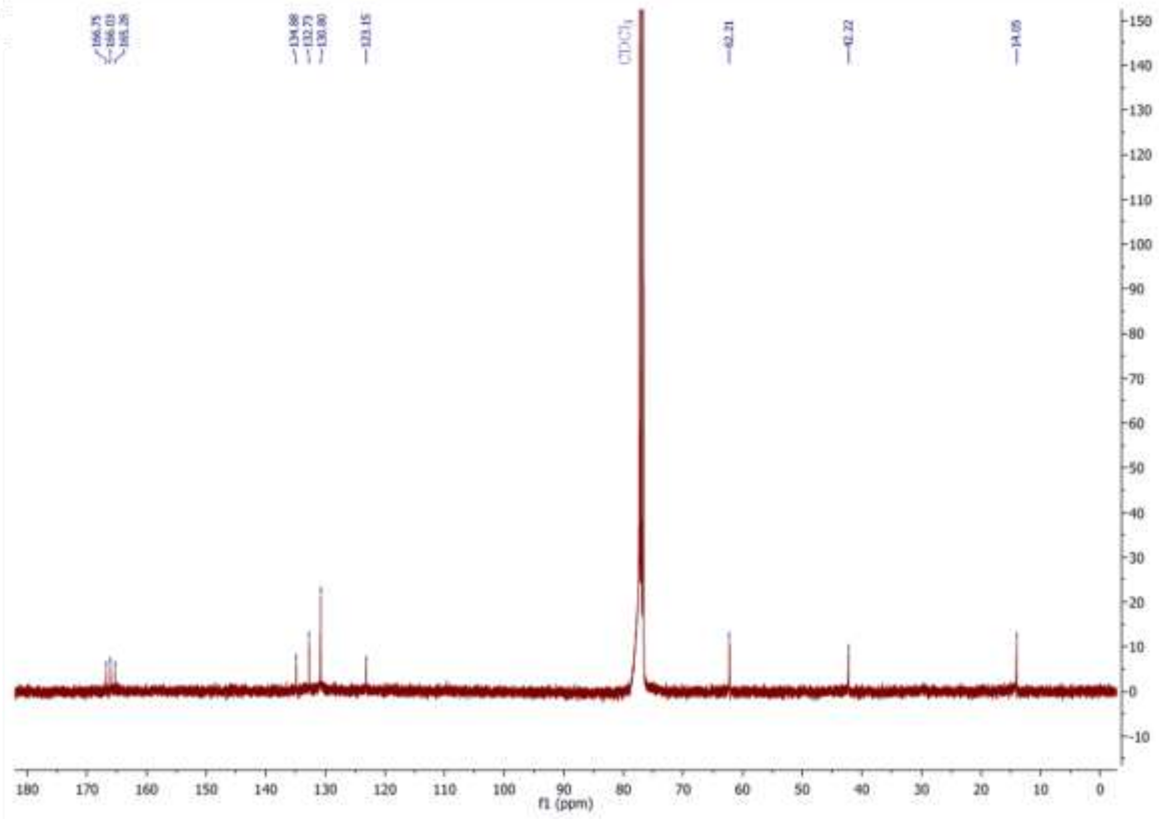

Compound **8**

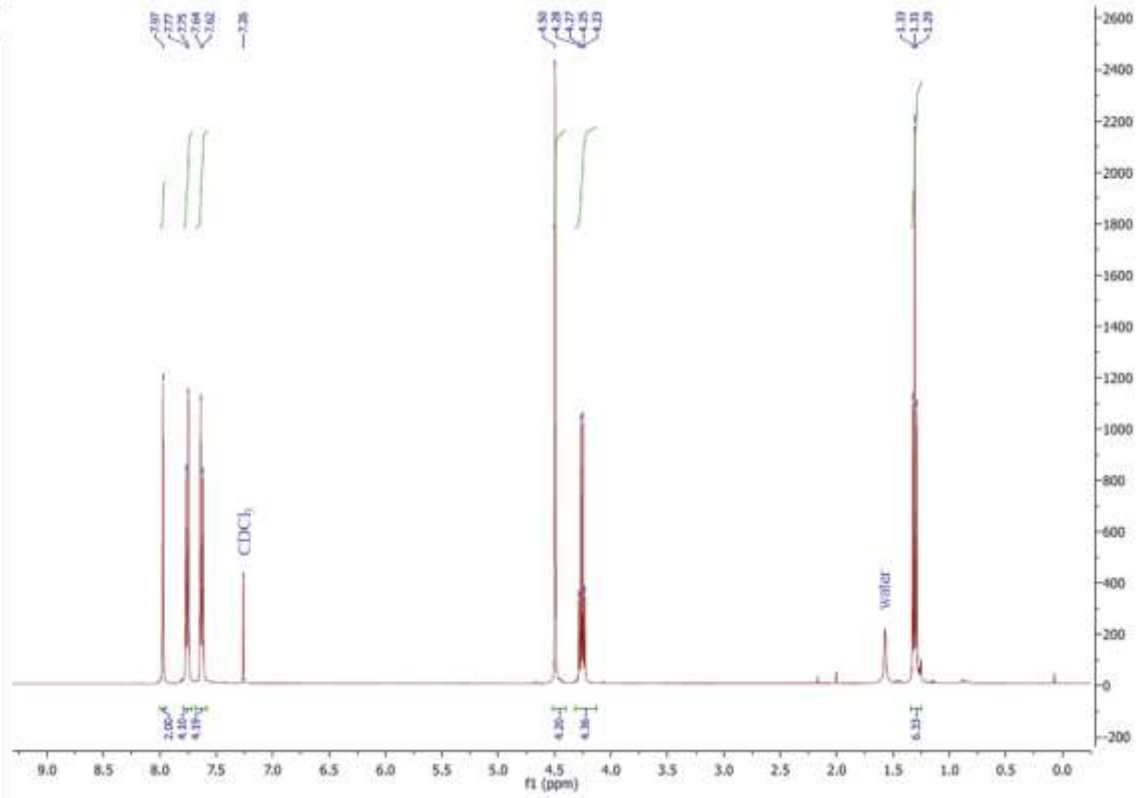

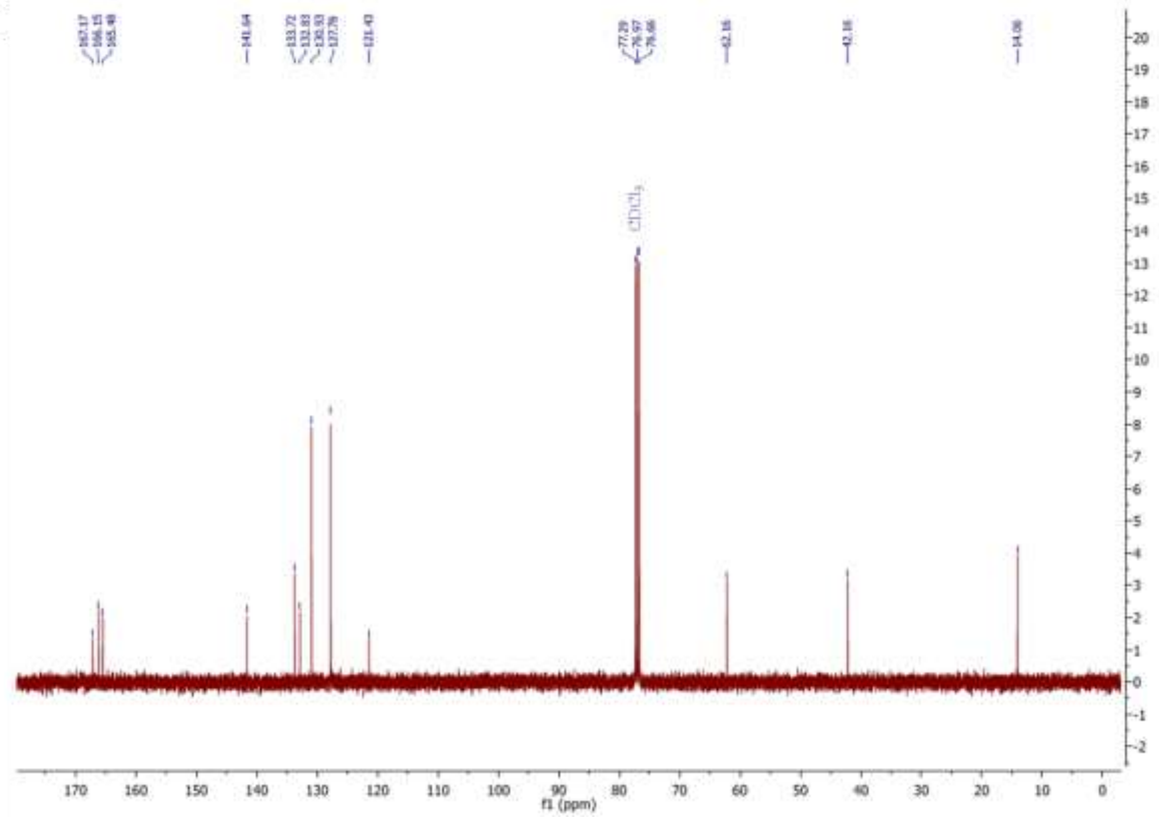

Compound **9**

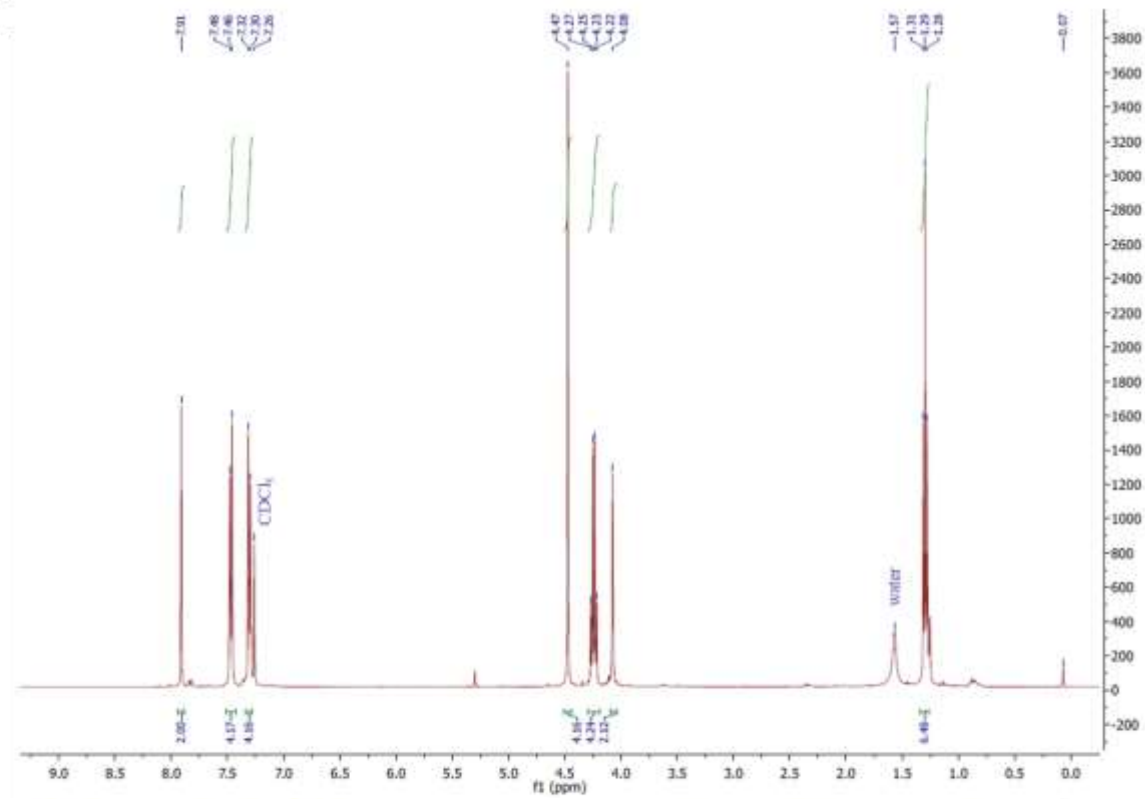

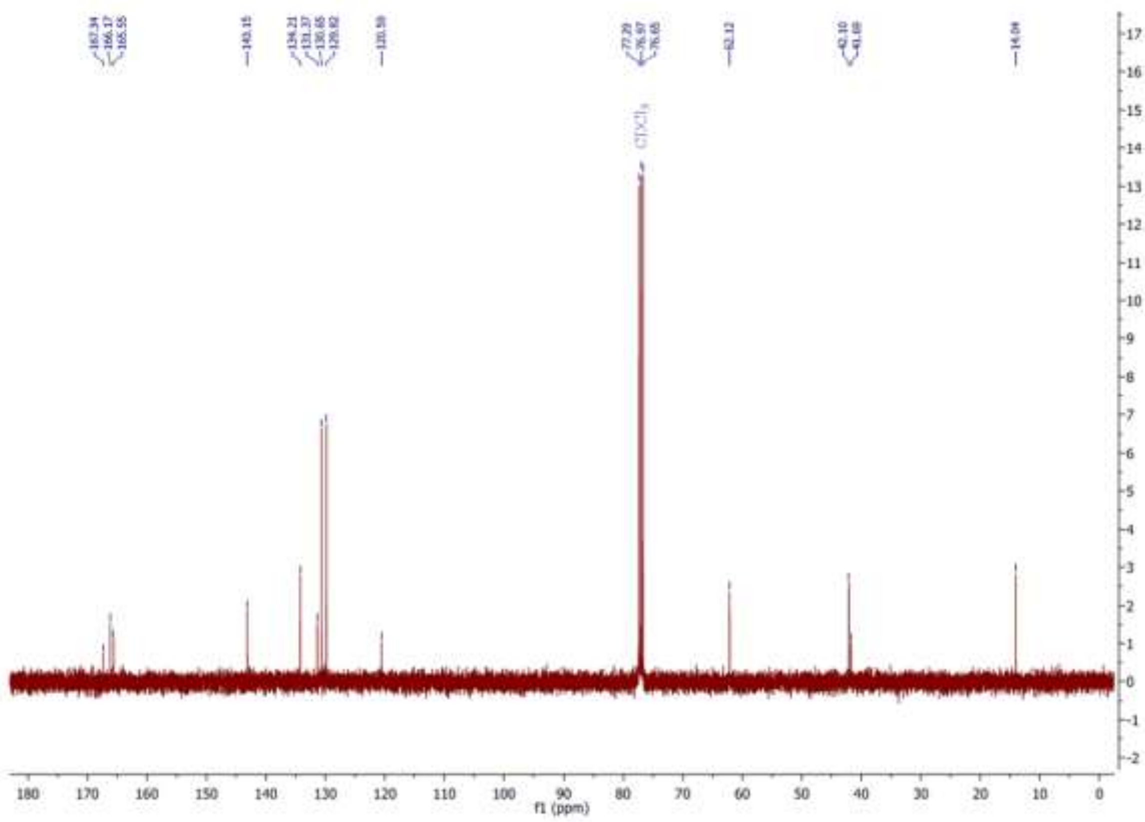

Compound **10**

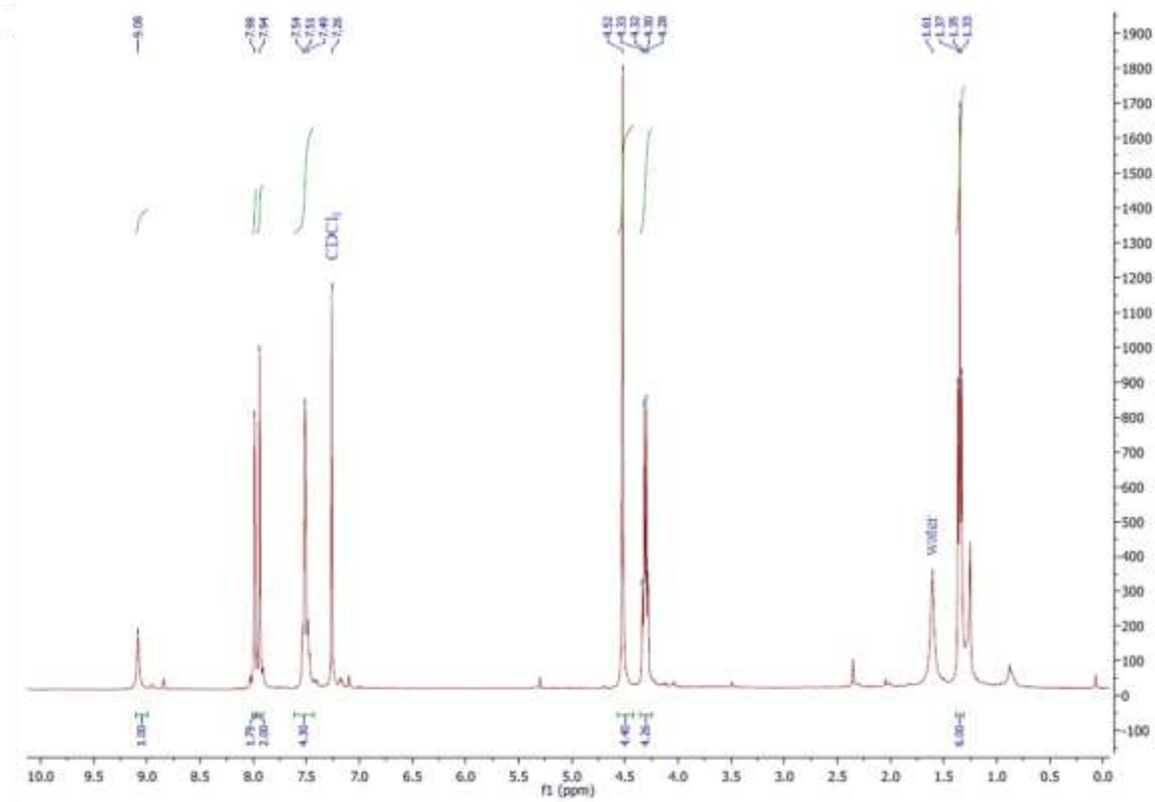
9.08
7.98
7.94
7.54
7.51
7.49
7.26
4.52
4.33
4.32
4.30
4.28
1.61
1.37
1.35
1.33
CDCl3
water
1.00
1.79
2.00
4.30
4.40
4.26
6.00
f1 (ppm)

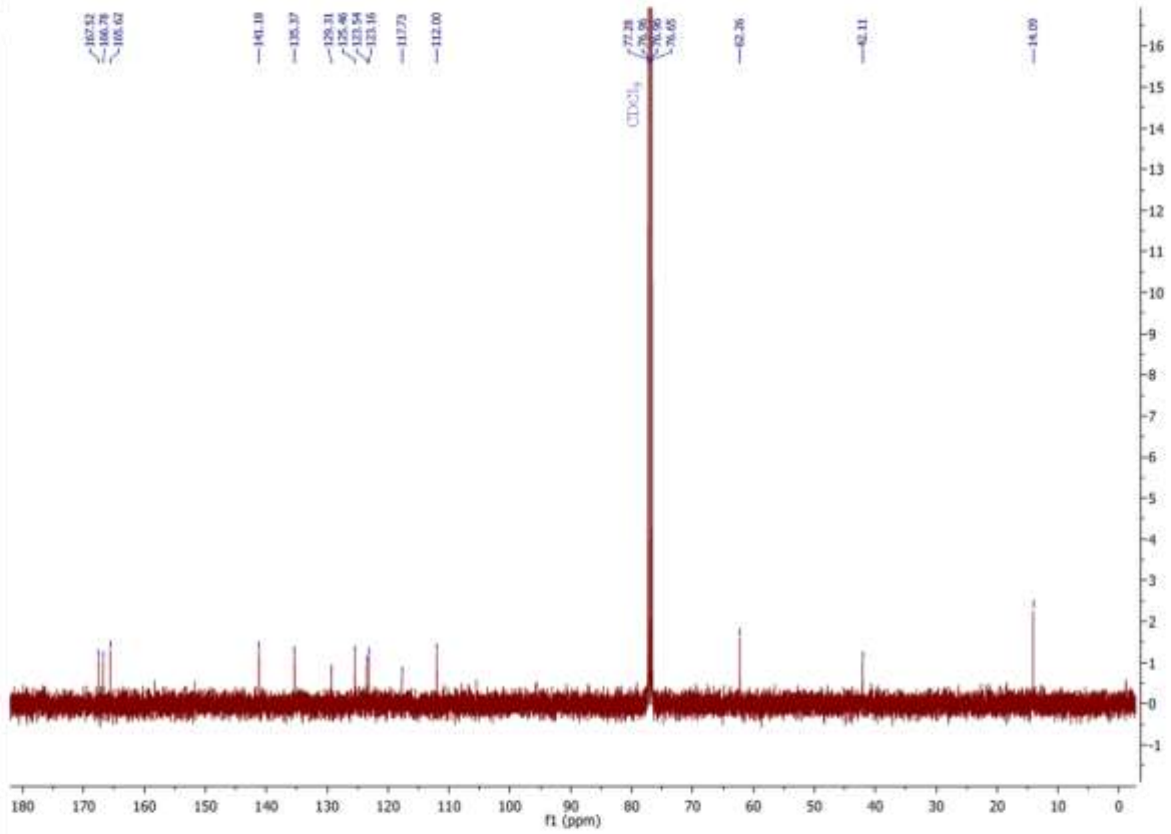
167.52
166.78
165.62
141.18
135.37
129.31
125.46
123.54
123.16
117.73
112.00
77.28
76.65
62.26
42.11
14.09
CDCl3
f1 (ppm)

Compound **11**

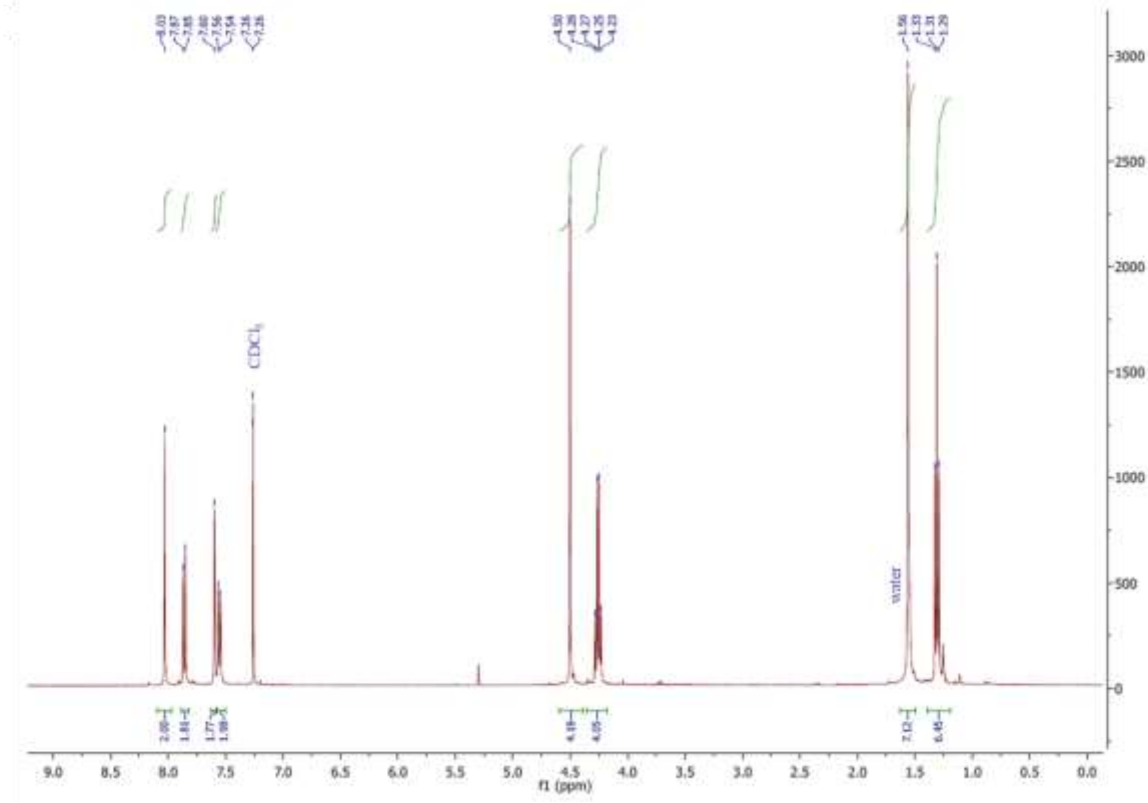

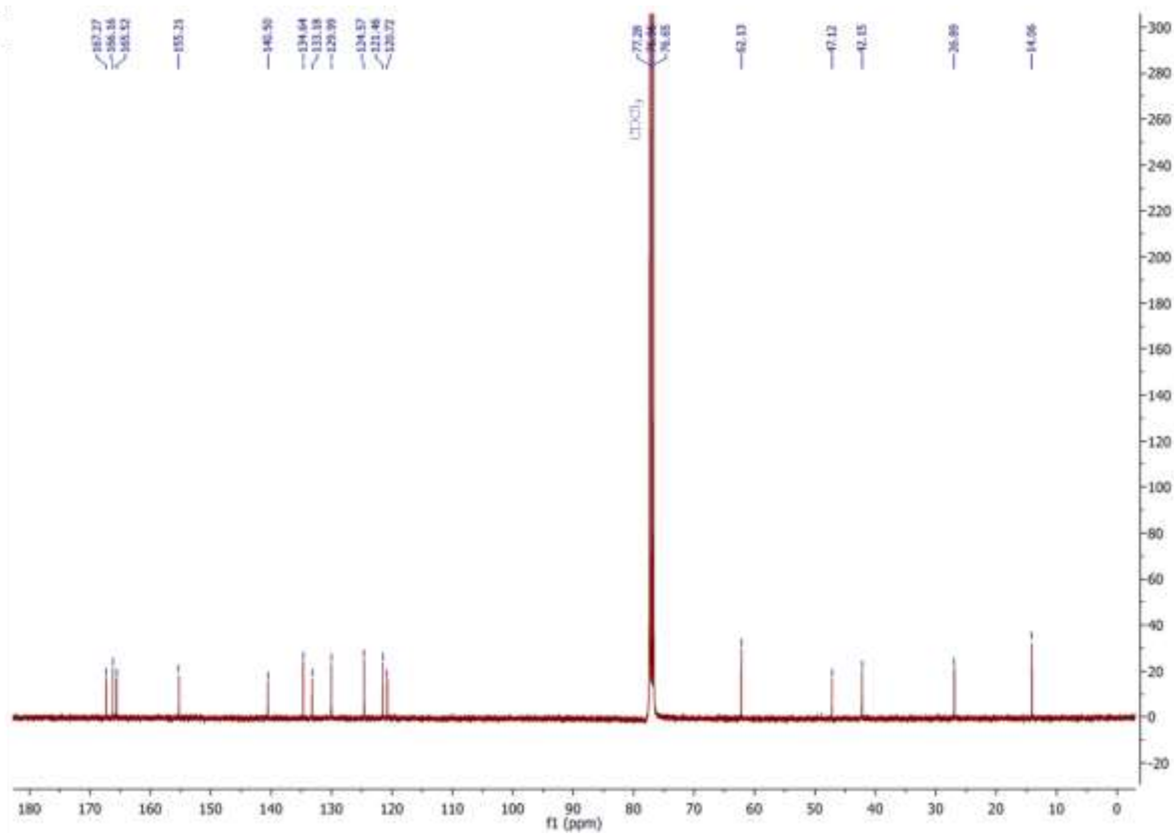

Compound **12**

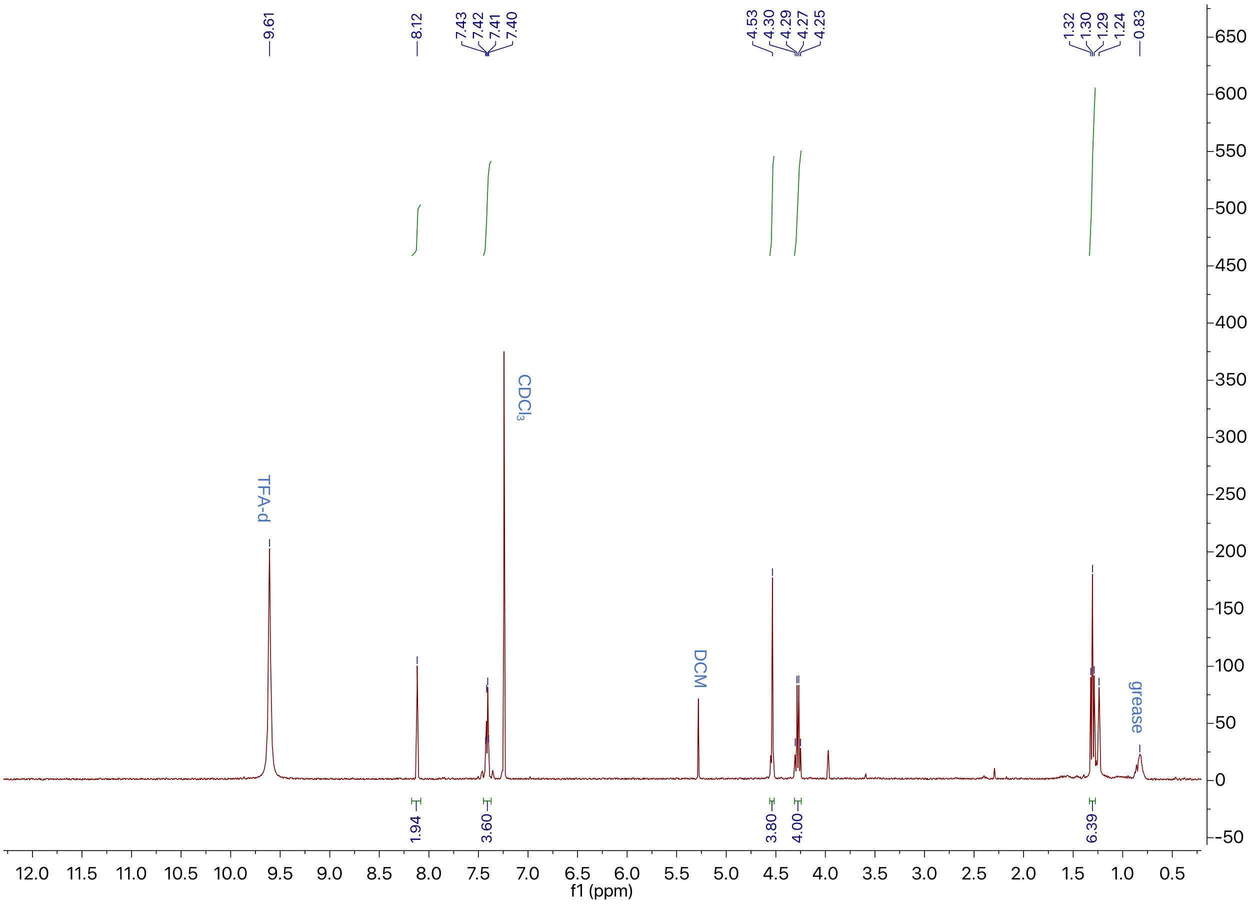
9.61
8.12
7.43
7.42
7.41
7.40
4.53
4.30
4.29
4.27
4.25
1.32
1.30
1.29
1.24
0.83
CDCl3
TFA-d
DCM
grease
1.94
3.60
3.80
4.00
6.39
f1 (ppm)

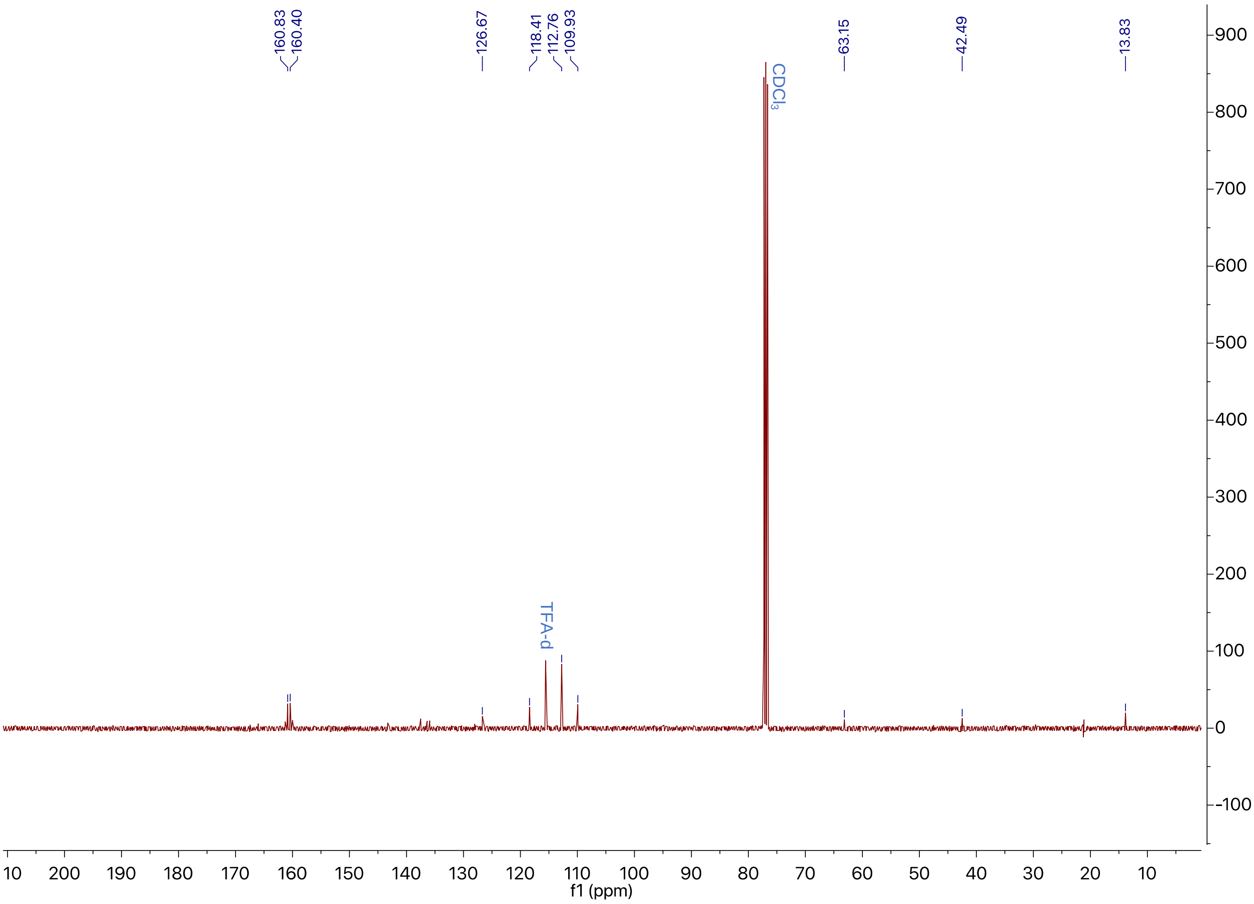
160.83
160.40
126.67
118.41
112.76
109.93
63.15
42.49
13.83
CDCl3
TFA-d
f1 (ppm)

Compound **13**

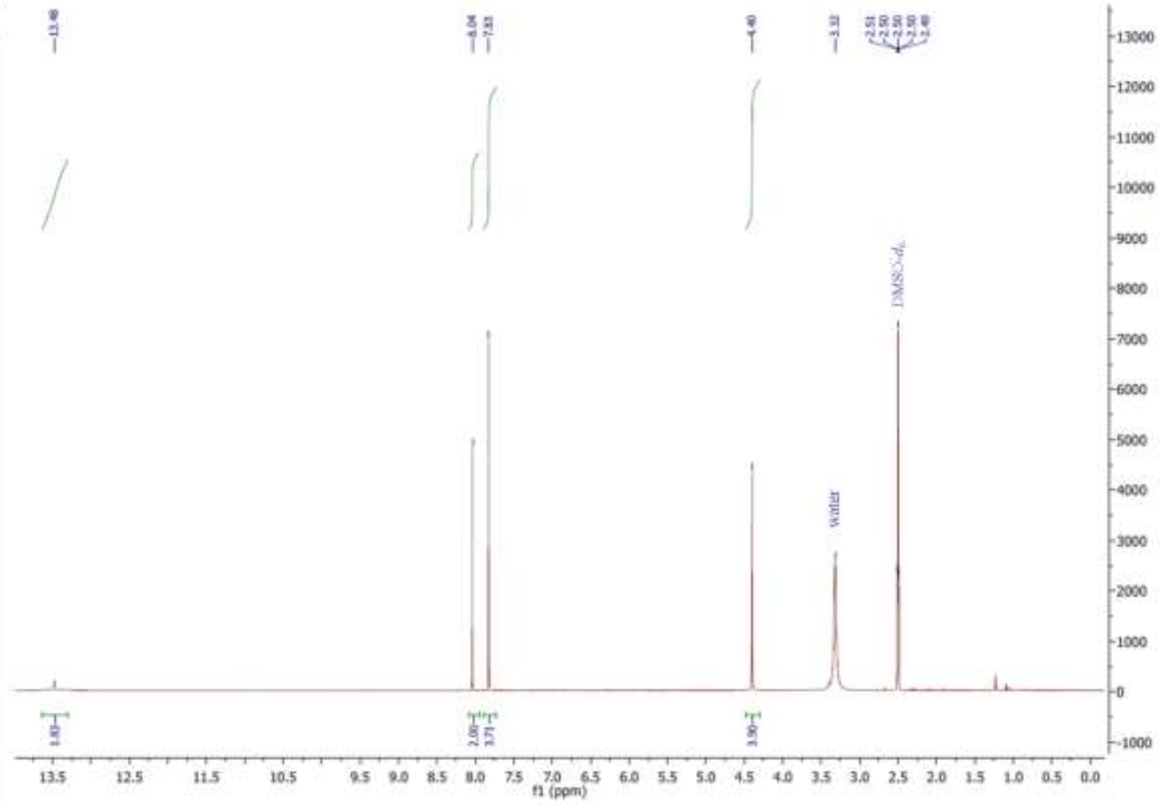

13.48
8.04
7.83
4.40
3.32
2.51
2.50
2.50
2.50
2.49
DMSO-d_6
water
1.83
2.00
3.71
3.90
f1 (ppm)

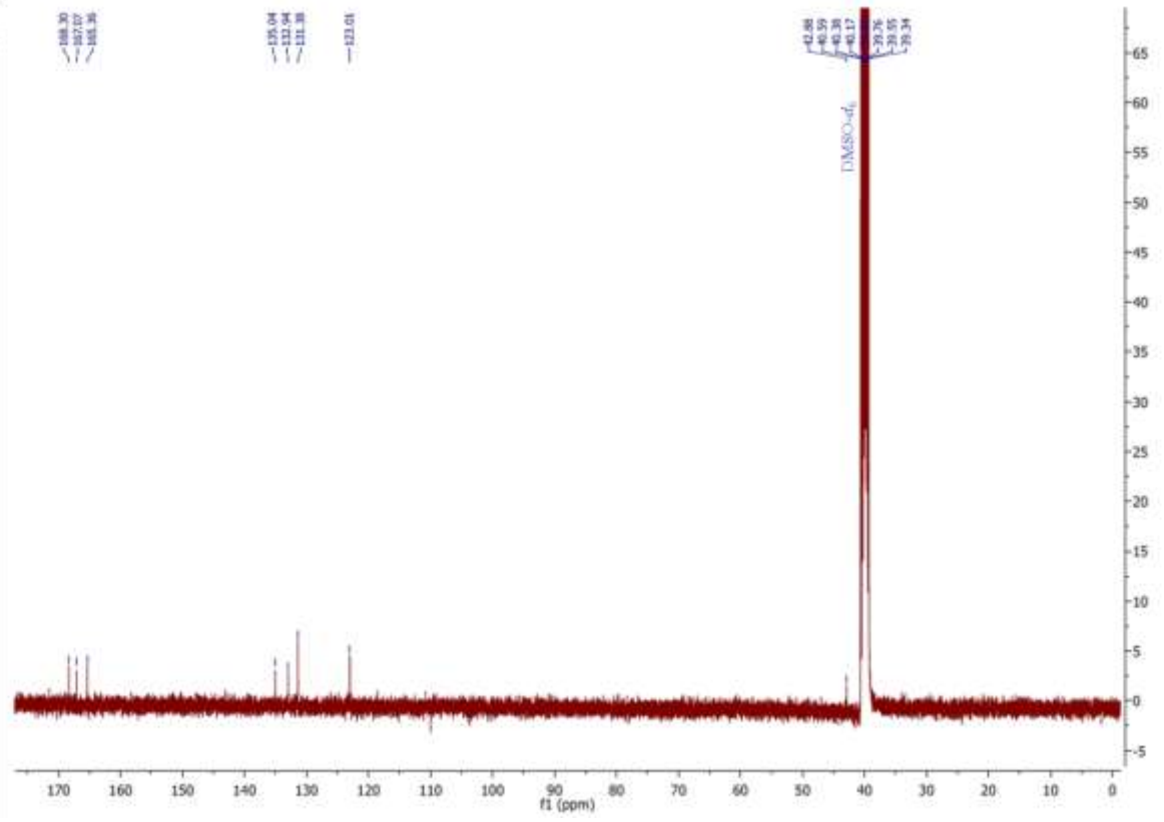

168.30
167.07
165.36
135.04
132.94
131.38
123.01
42.88
40.59
40.38
40.17
39.76
39.55
39.34
DMSO-d_6
f1 (ppm)

Compound **14**

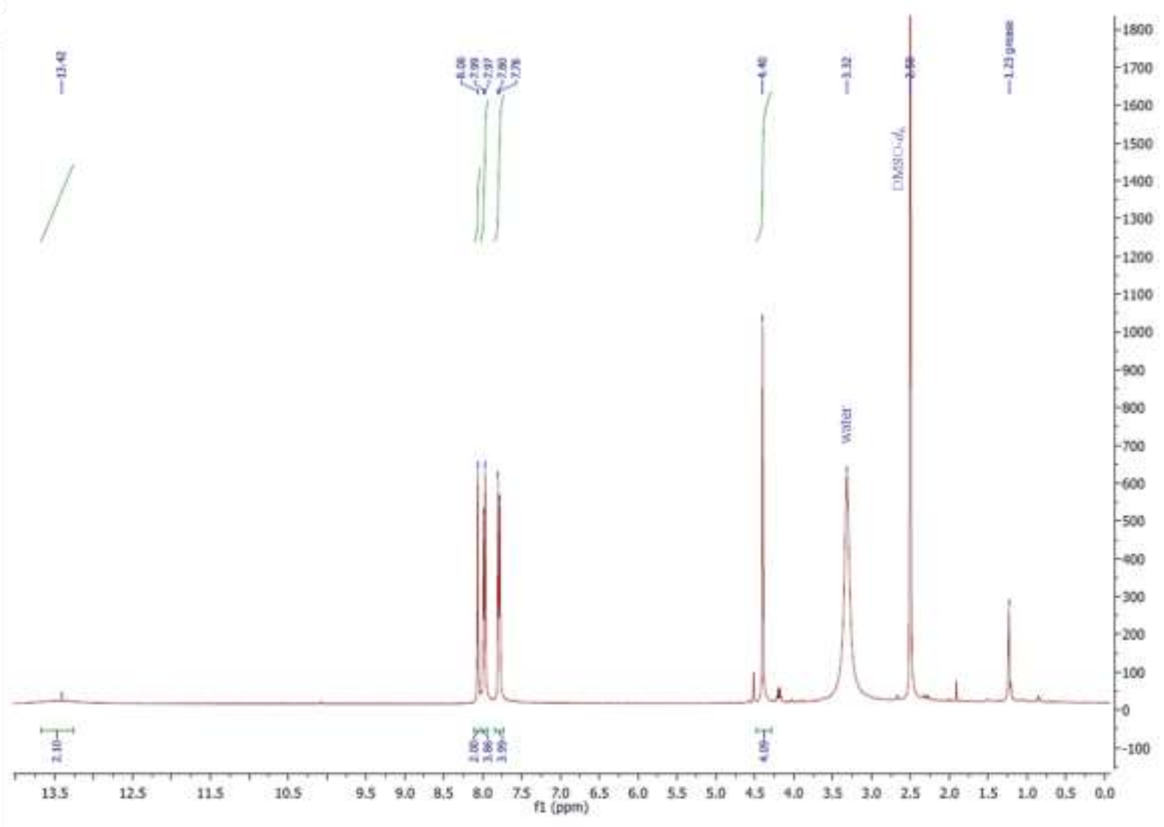

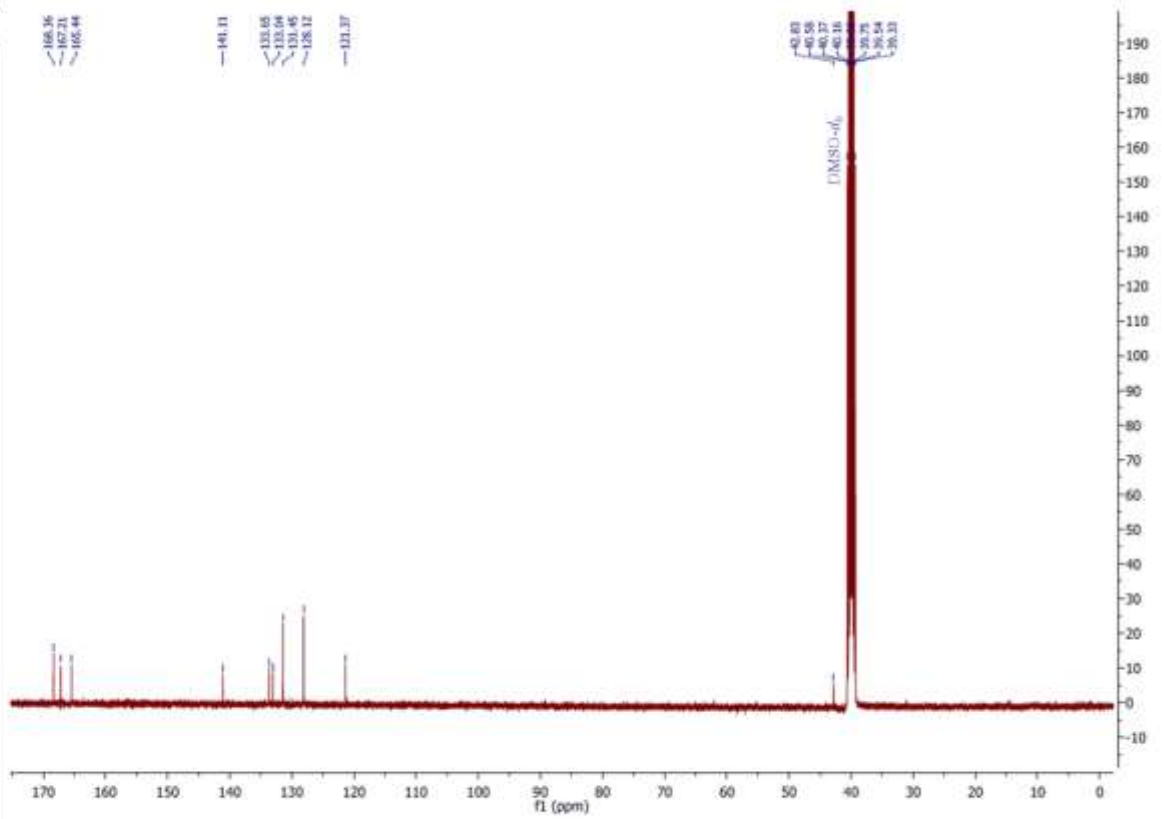

Compound **15**

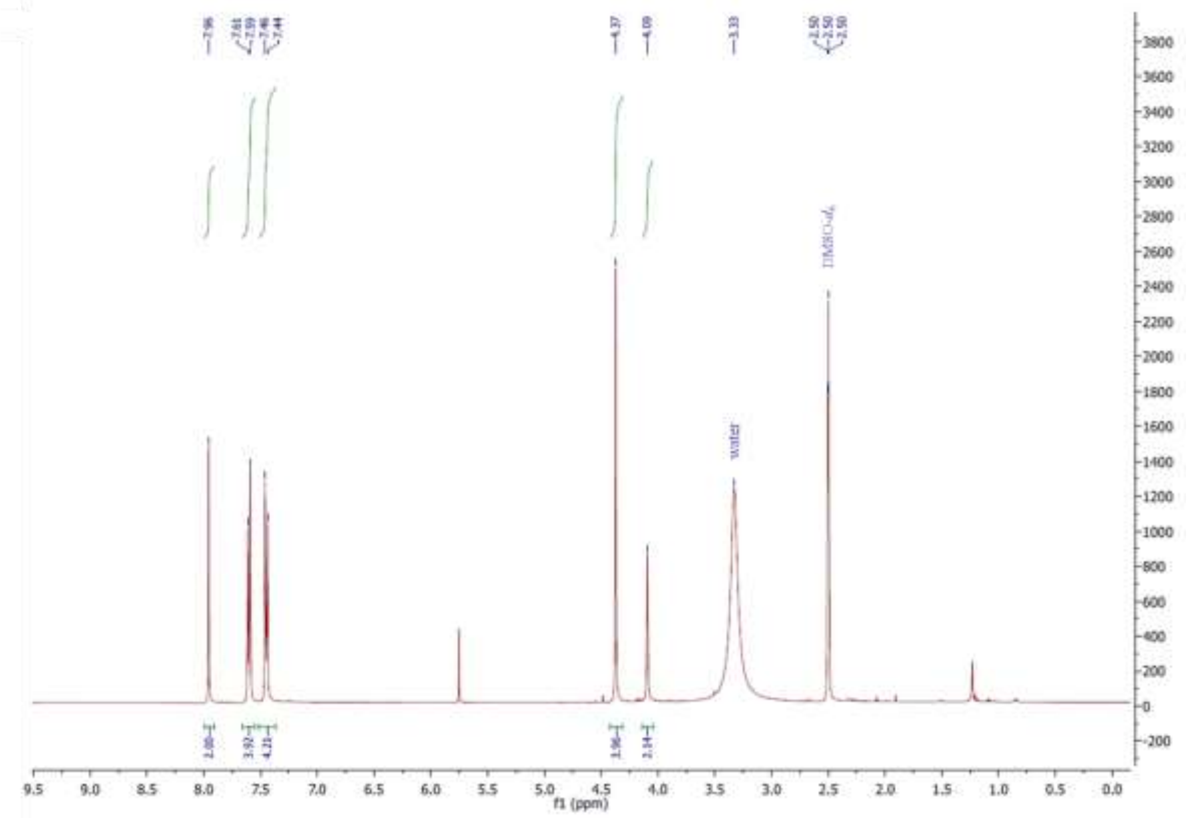

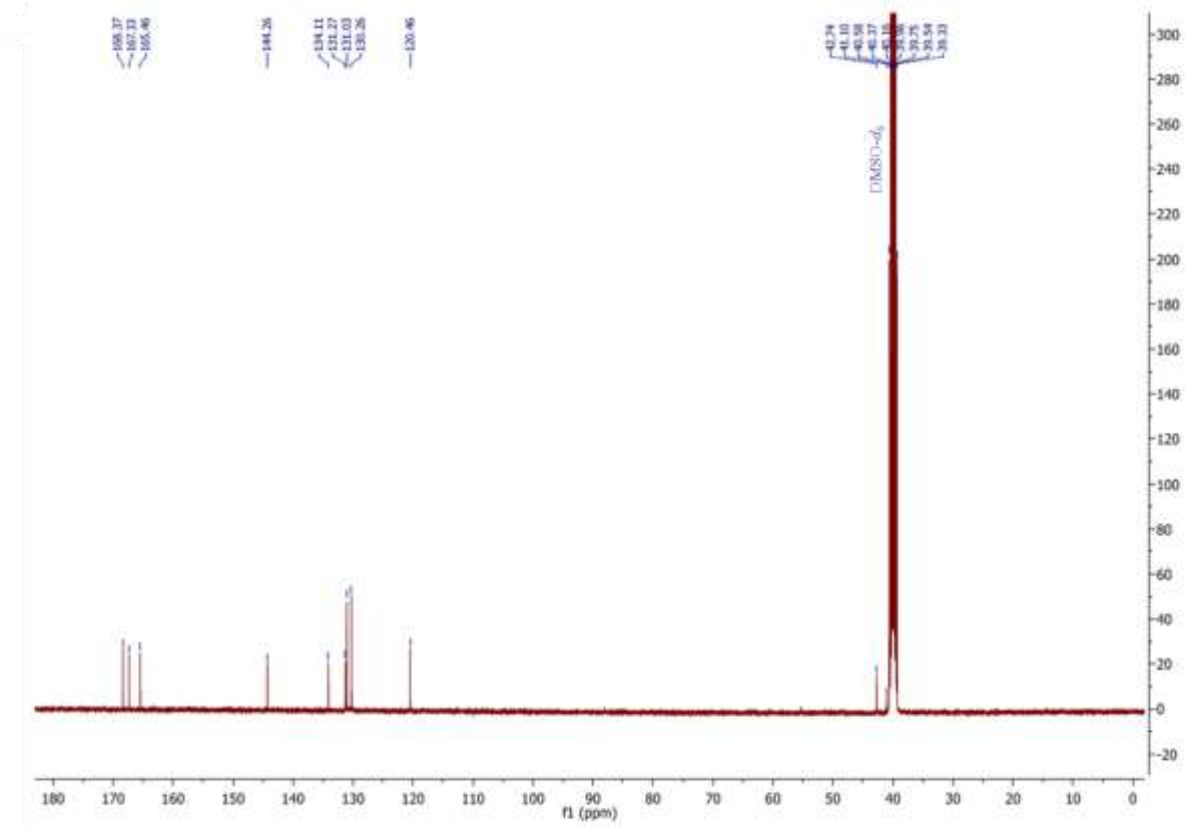

Compound **16**

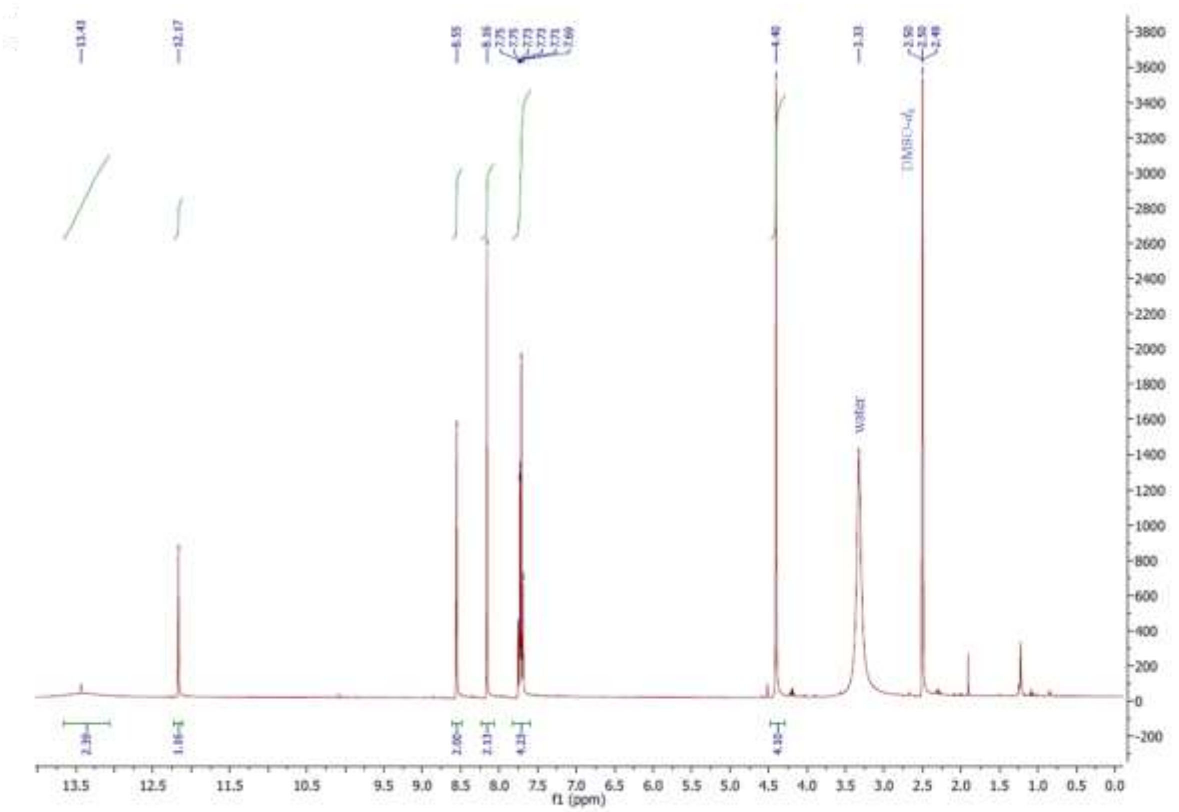
13.43
12.17
8.55
8.16
4.40
3.33
2.50
2.50
2.49
DMSO-d6
water
2.39
1.16
2.00
2.13
4.23
4.10
f1 (ppm)

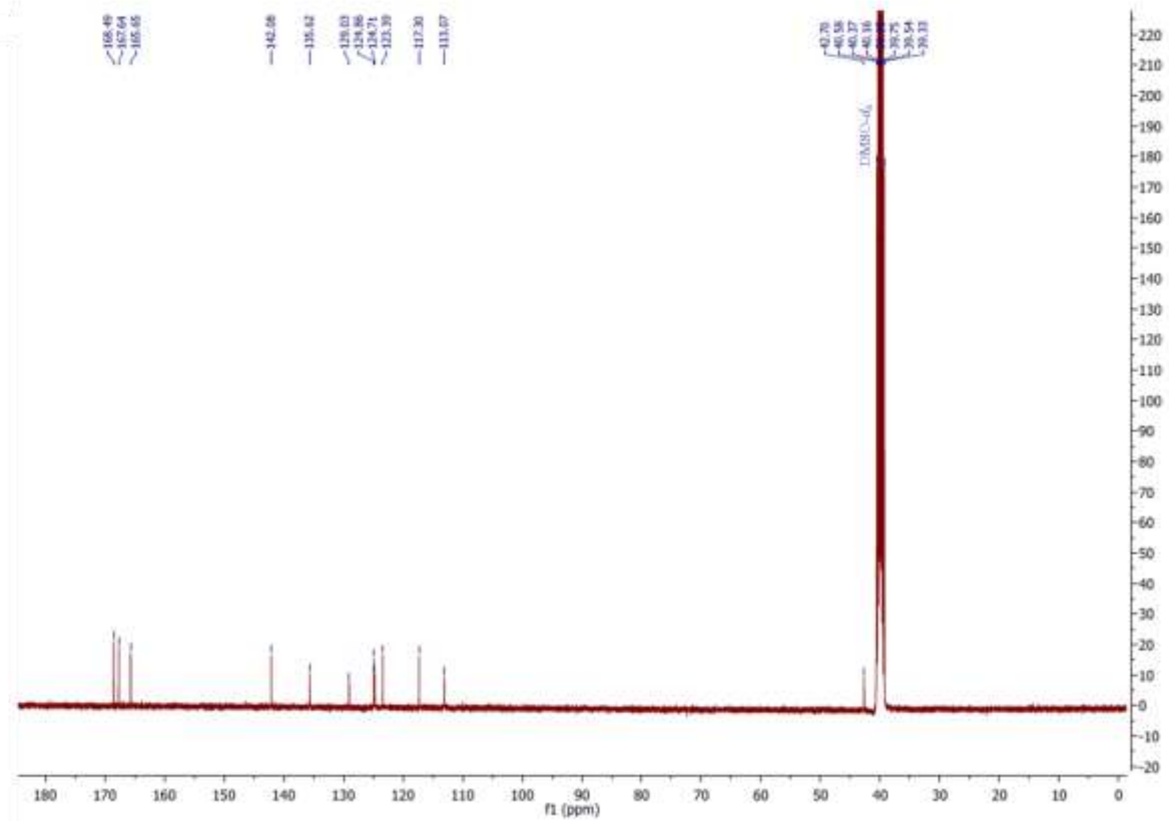
168.49
167.64
165.65
142.08
135.62
129.03
124.86
124.71
123.39
117.30
113.07
DMSO-d6
f1 (ppm)

Compound **17**

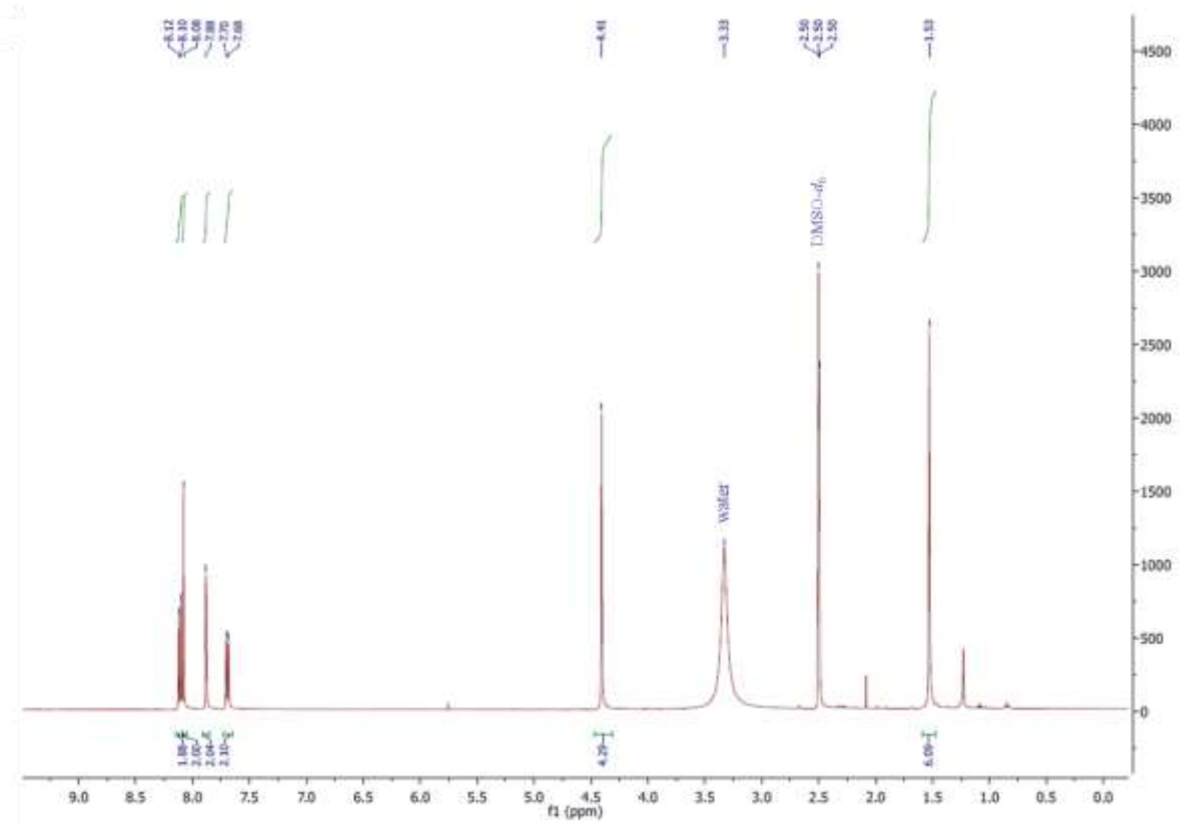

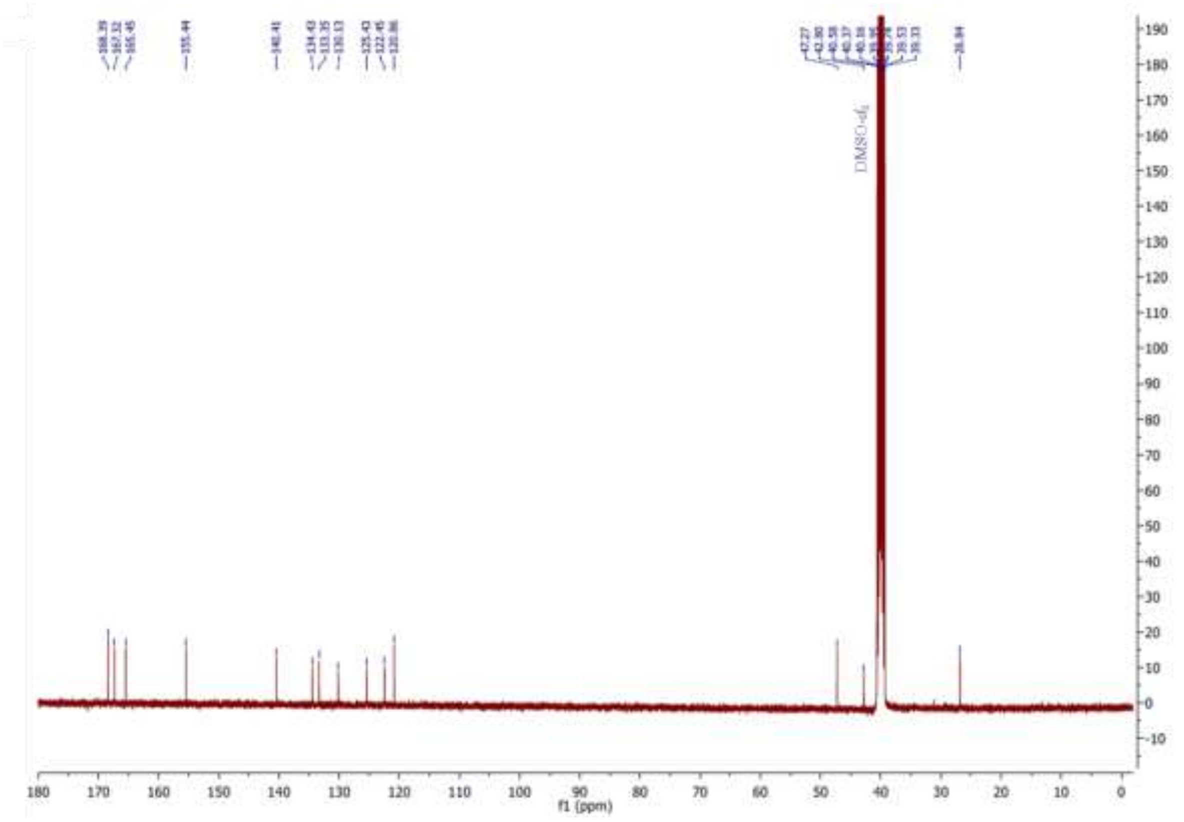

Compound **18**

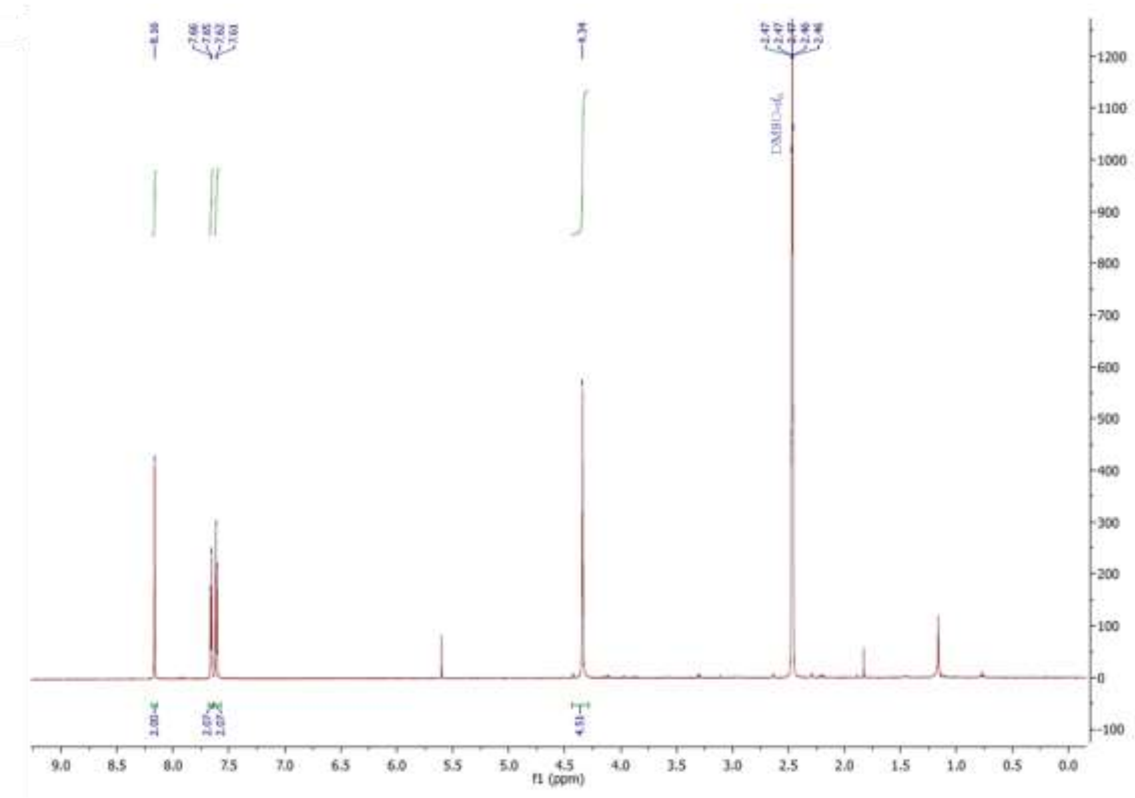

We were unable to acquire ^{13}C-NMR, due to the low solubility of the compound.

Compound **19**

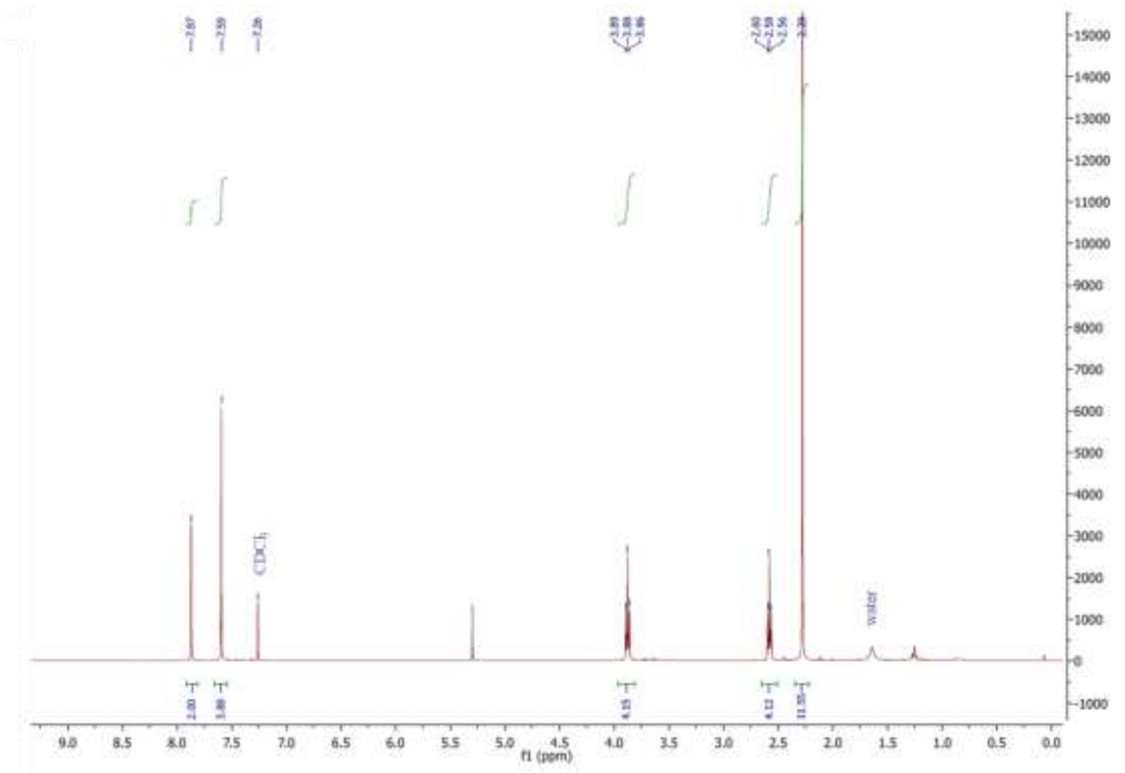

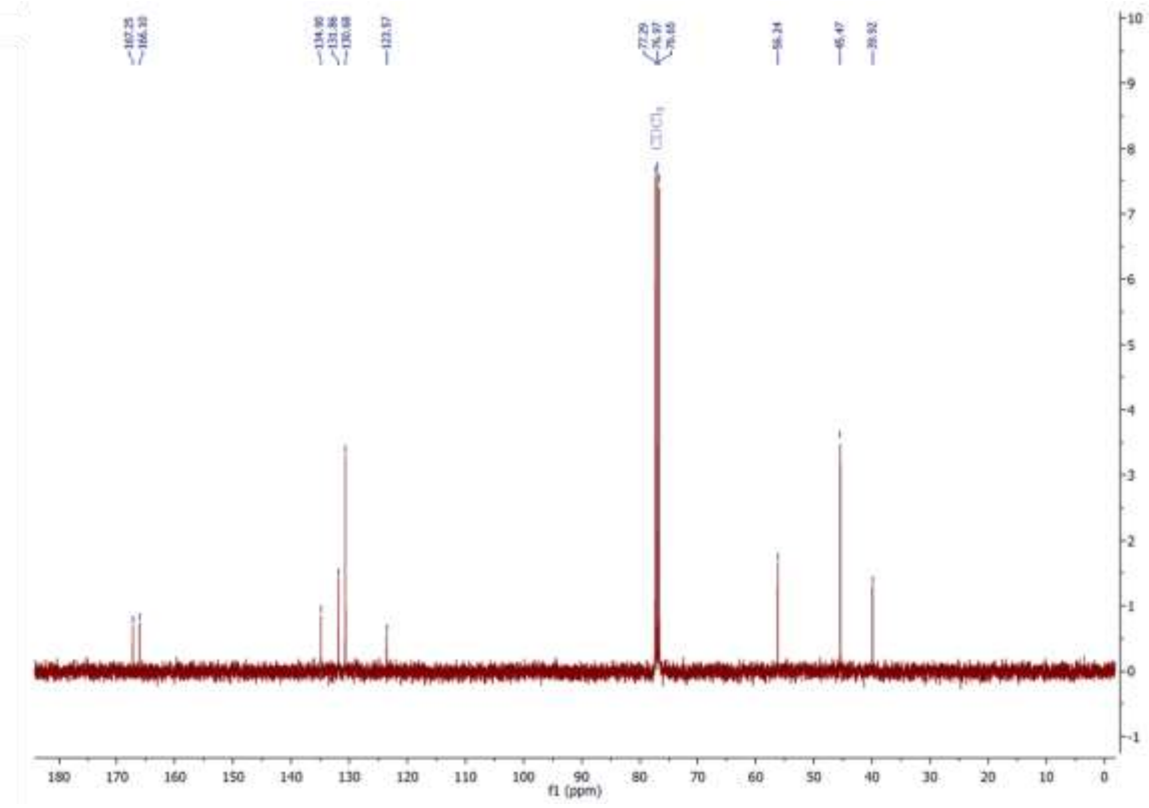

Compound **20**

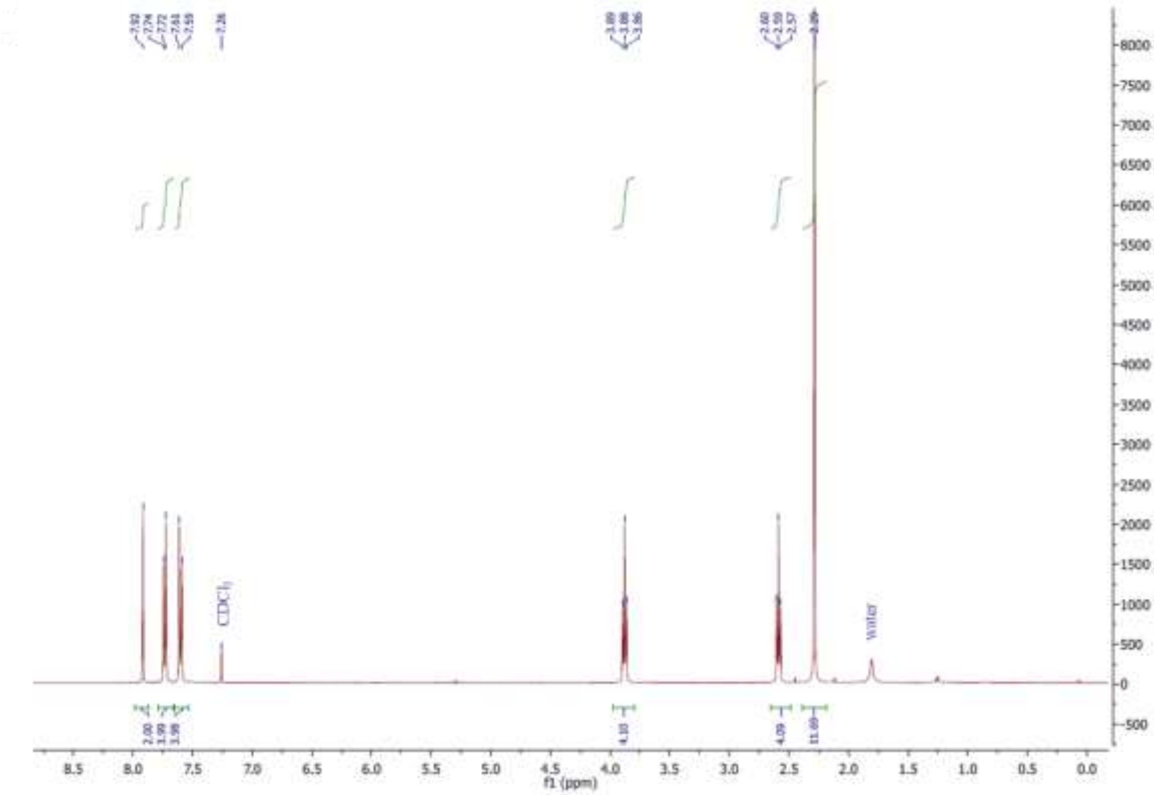

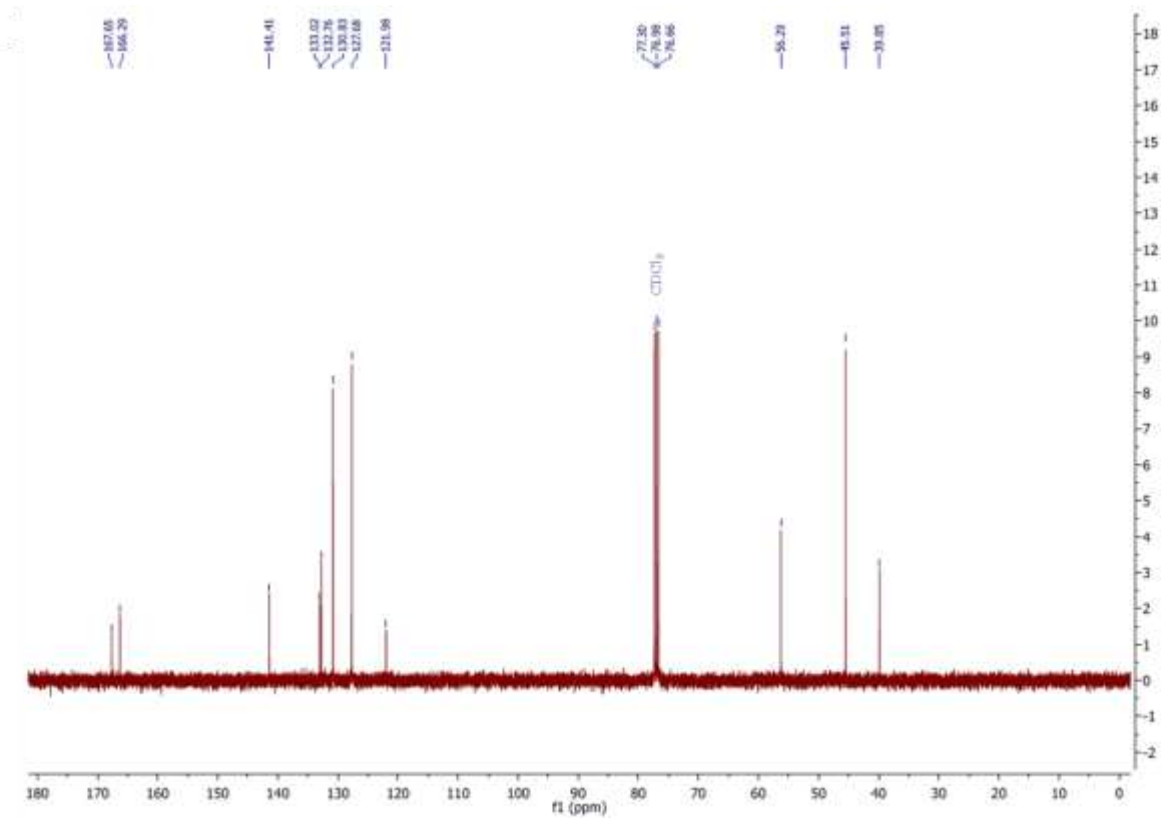

Compound **21**

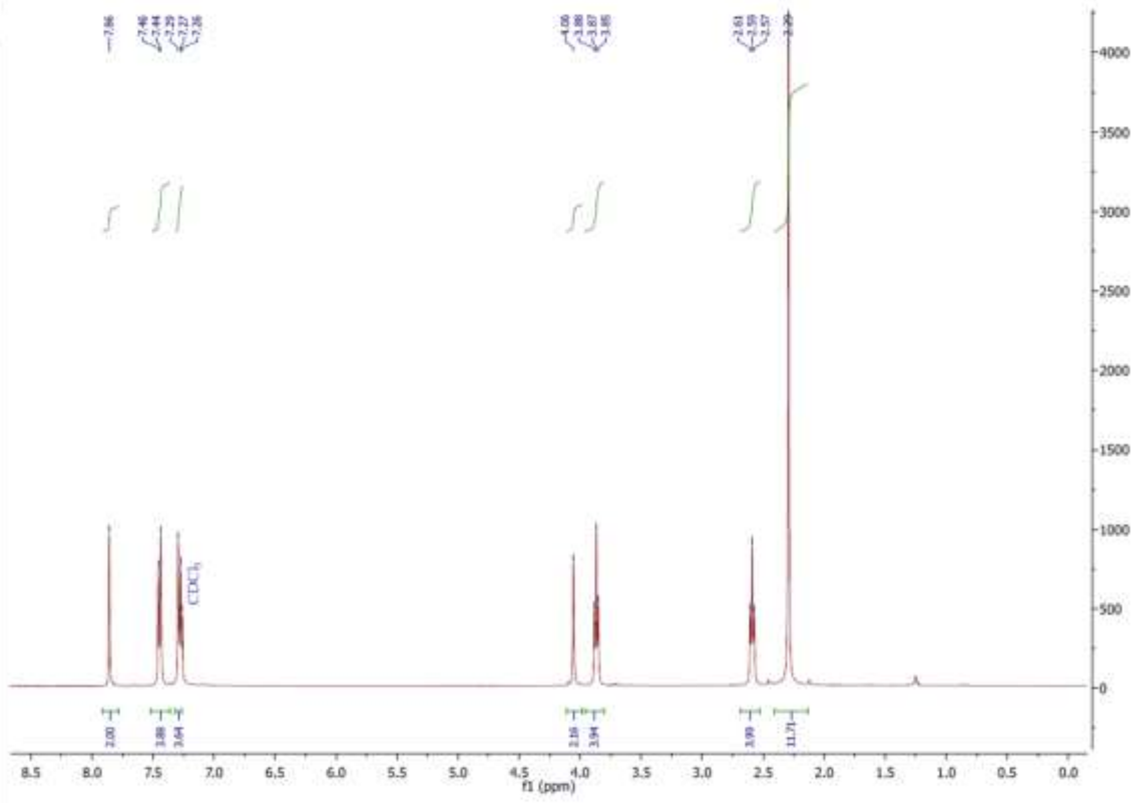

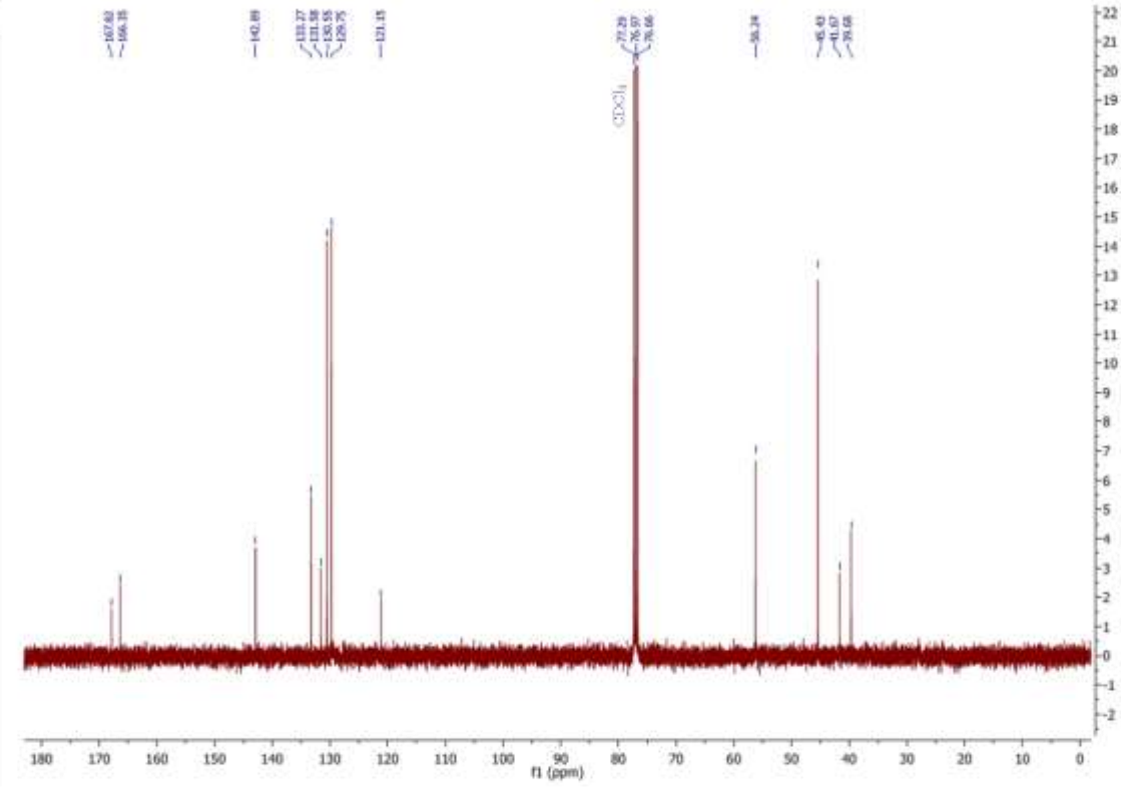

Compound **22**

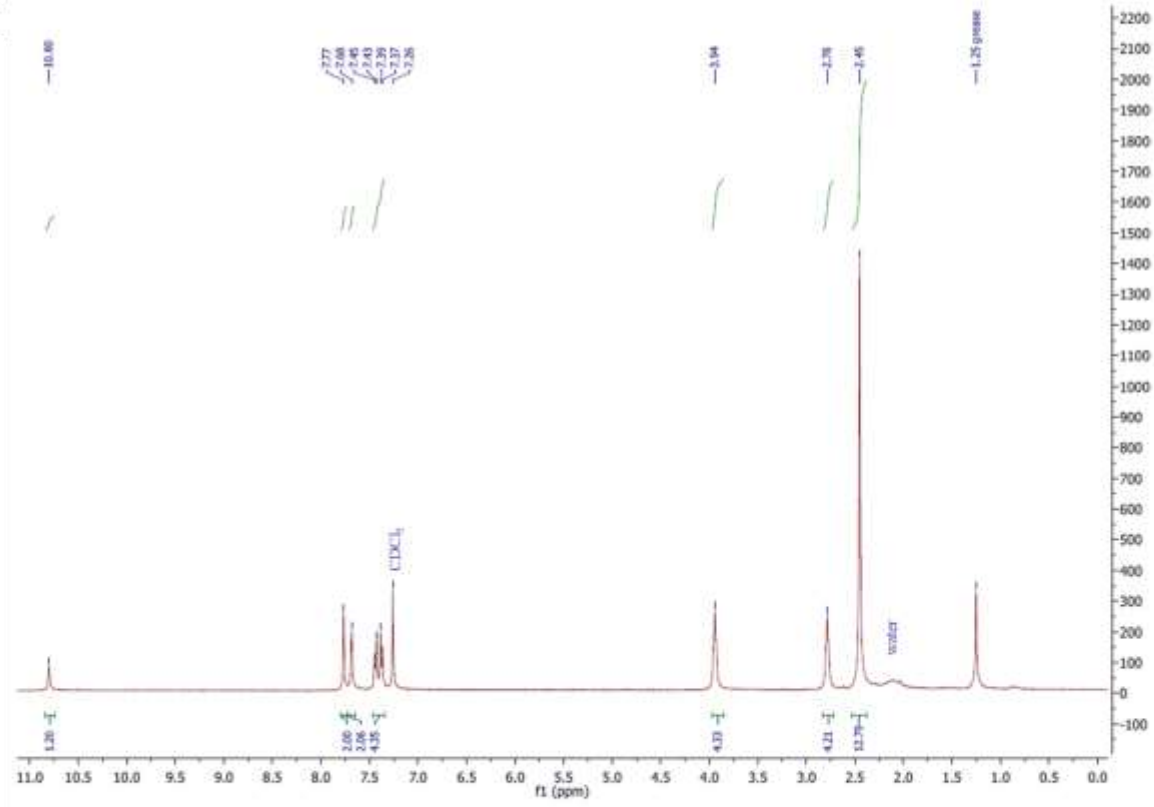

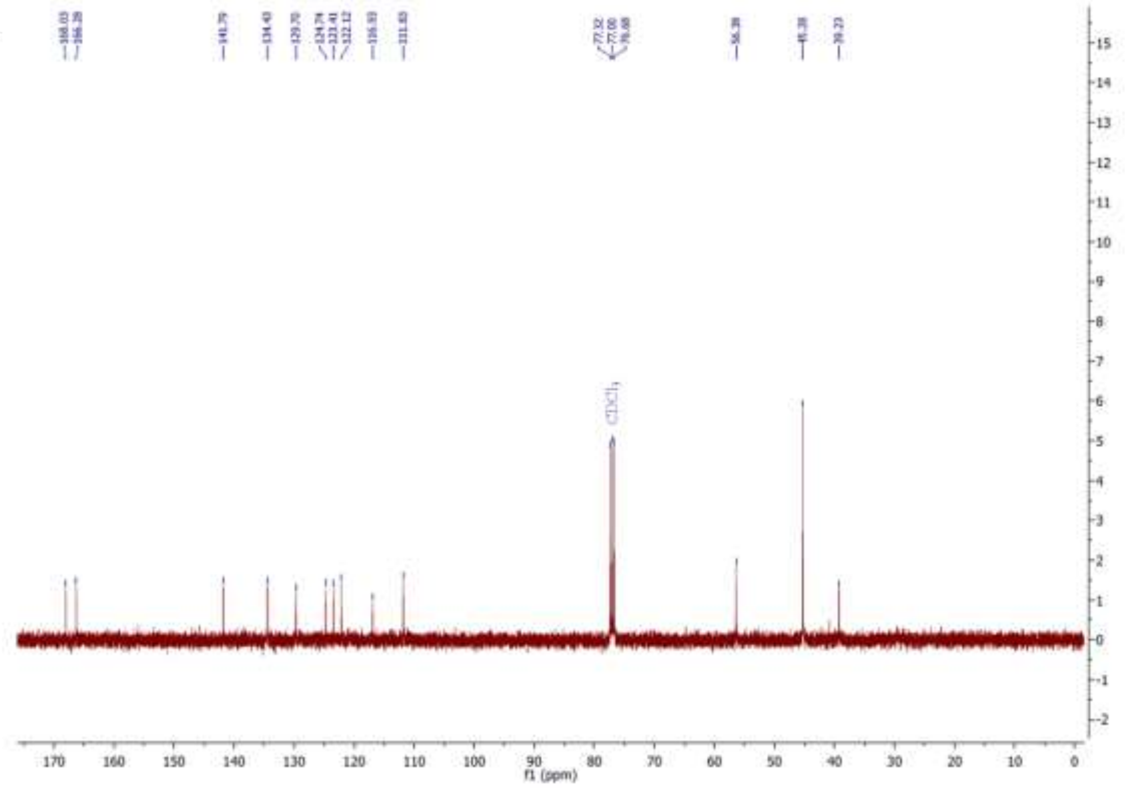

Compound **23**

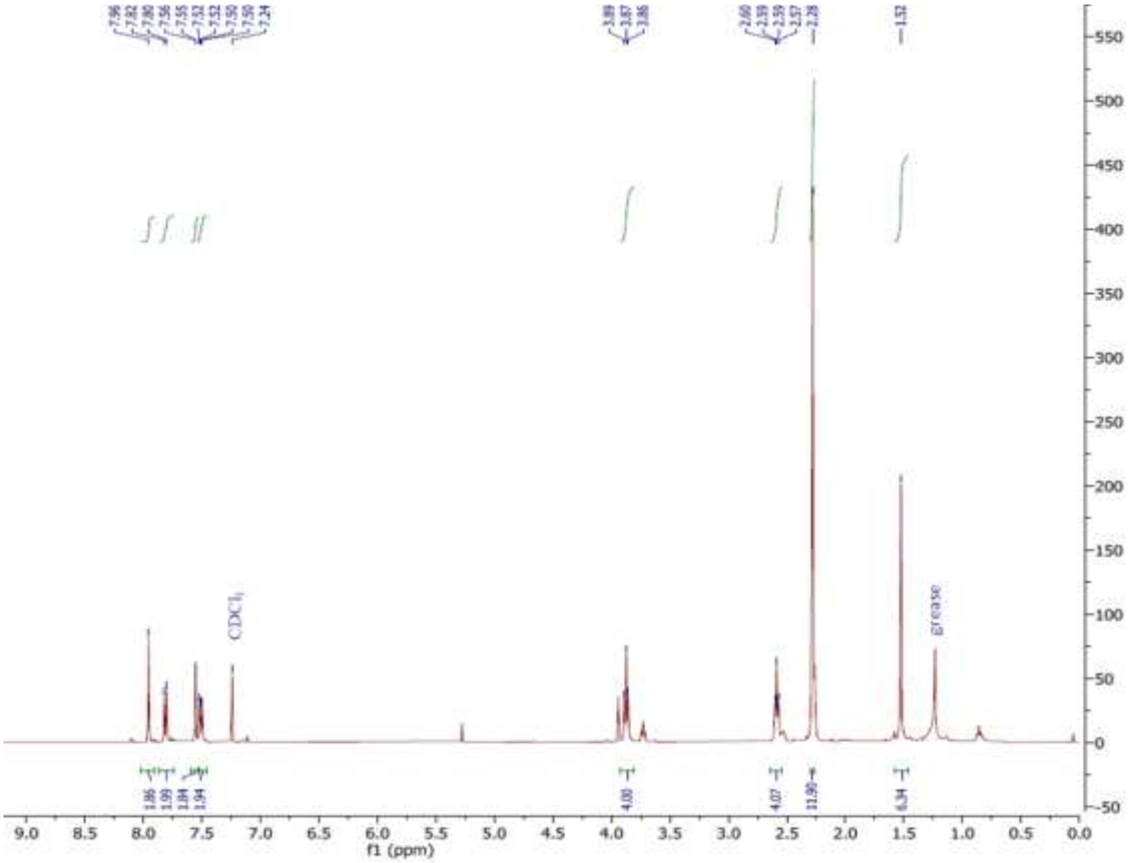

CDCl3
grease
f1 (ppm)

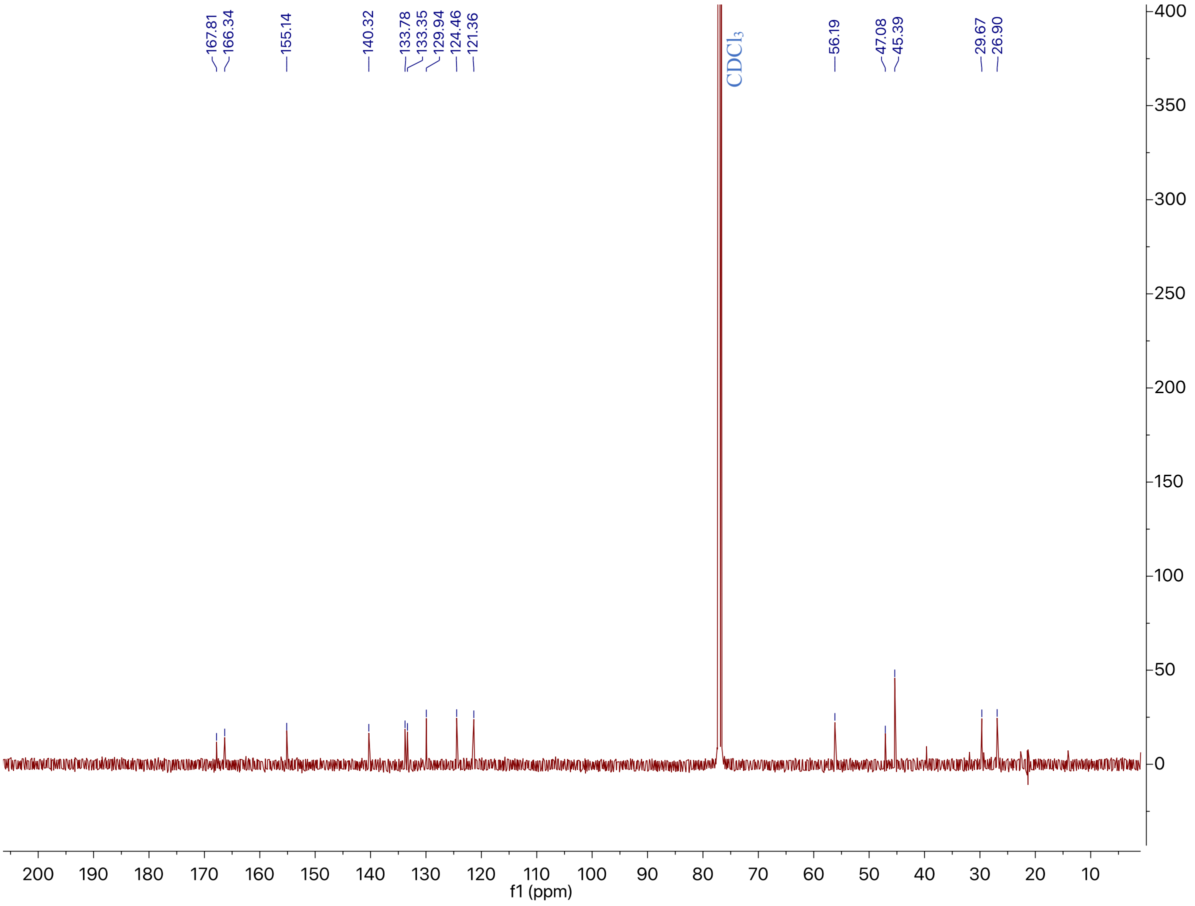

167.81
166.34
155.14
140.32
133.78
133.35
129.94
124.46
121.36
CDCl3
56.19
47.08
45.39
29.67
26.90
f1 (ppm)

Compound **24**

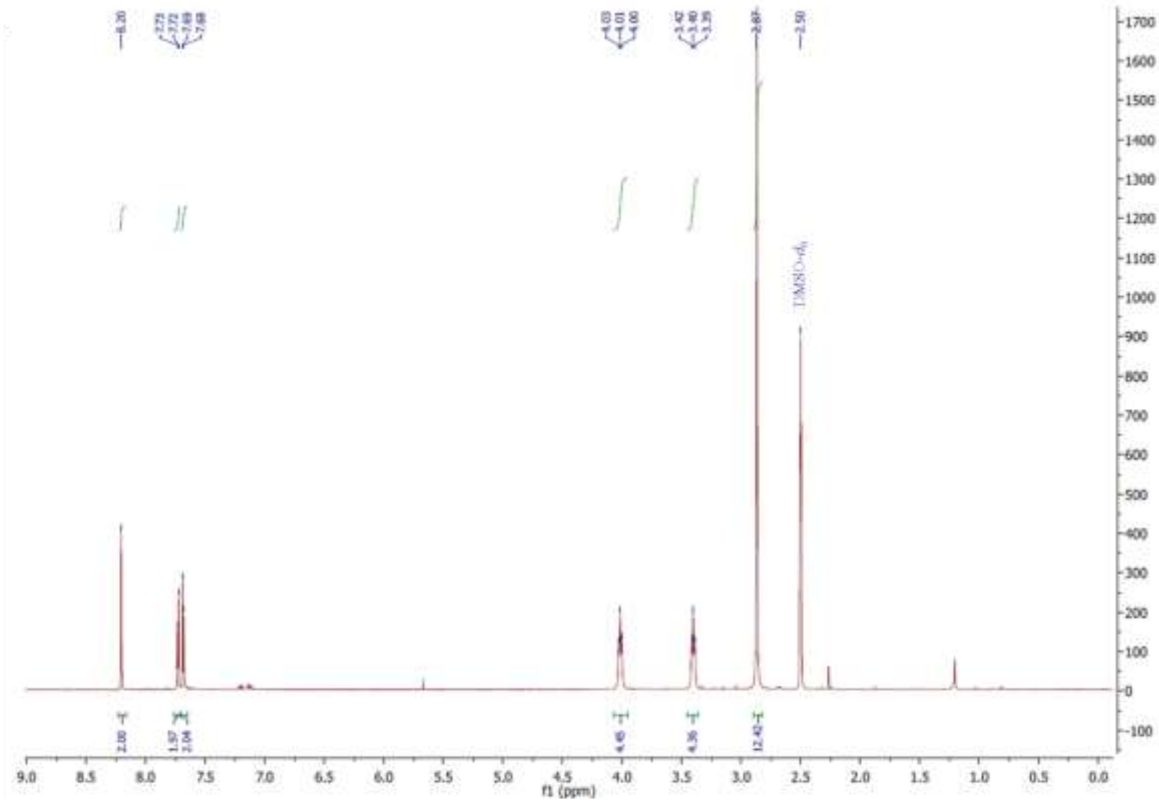

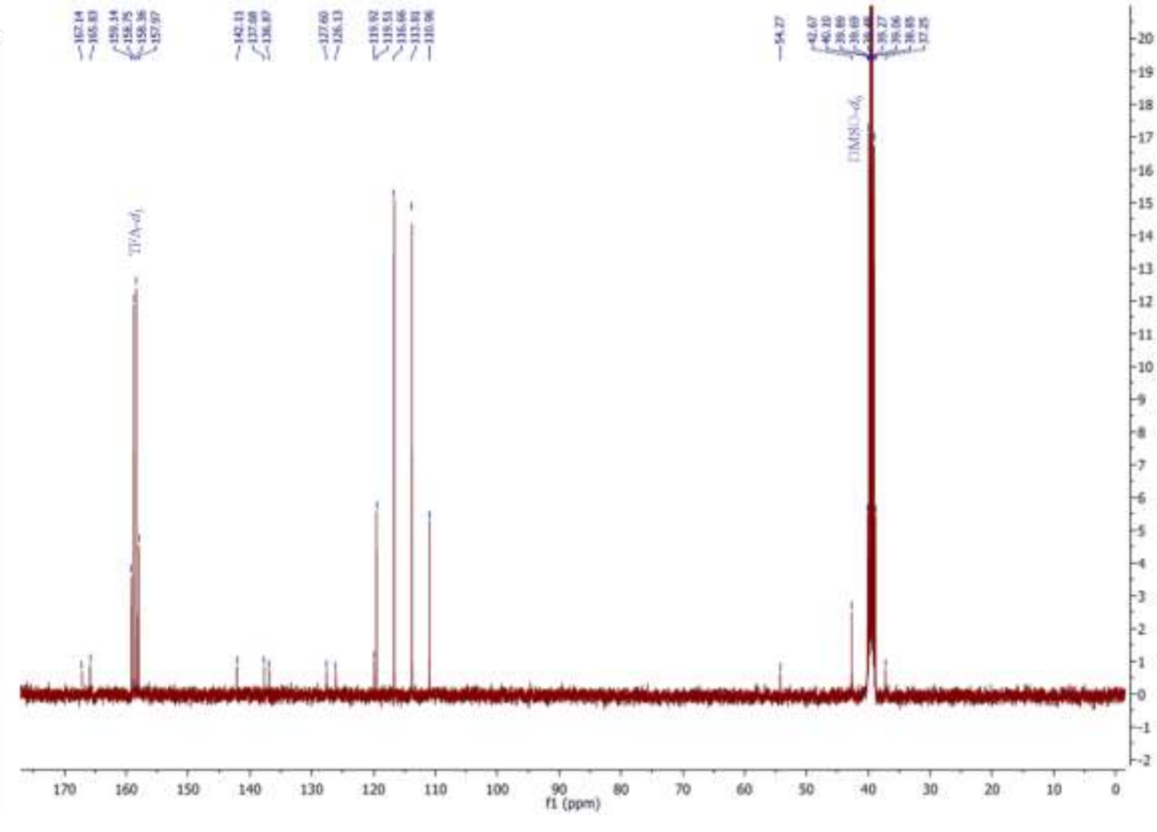

Supplement: Supplementary file 1 — cn2c00357_si_001.pdf [file cn2c00357_si_001.pdf]
